# Supplementary material for: Effect of perioperative dexmedetomidine on sleep quality in adult patients after noncardiac surgery: A systematic review and meta-analysis of randomized trials
Source: PLoS One. 2024 Dec 5;19(12):e0314814. doi: 10.1371/journal.pone.0314814 (PMC11620464; doi:10.1371/journal.pone.0314814)
Supplement: S2 File — (DOCX) [file pone.0314814.s002.docx]

**S1 File:** Search Strategies

**Search Strategy of Cochrane Library database.**

#1 MeSH descriptor: [Dexmedetomidine] explode all trees

#2 (dexmedetomidine):ti,ab,kw OR (Precedex):ti,ab,kw

#3 #1 OR #2

#4 MeSH descriptor: [Surgical Procedures, Operative] explode all trees

#5 (surger*):ti,ab,kw OR (surgical):ti,ab,kw OR (operat*):ti,ab,kw OR (perioperat*):ti,ab,kw OR (postoperat*):ti,ab,kw

#6 #4 OR #5

#7 MeSH descriptor: [Sleep] explode all trees

#8 (sleep*):ti,ab,kw OR (insomnia):ti,ab,kw OR (sleeplessness):ti,ab,kw OR (rest activity):ti,ab,kw

#9 #7 OR #8

#10 #3 AND #6 AND #9

**Search Strategy of PubMed database.**

Search: ((("Dexmedetomidine"[Mesh]) OR ((dexmedetomidine[Title/Abstract]) OR (precedex[Title/Abstract]))) AND (("Surgical Procedures, Operative"[Mesh]) OR (((((surger*[Title/Abstract]) OR (surgical[Title/Abstract])) OR (operat*[Title/Abstract])) OR (perioperat*[Title/Abstract])) OR (postoperat*[Title/Abstract])))) AND (("Sleep"[Mesh]) OR ((((sleep*[Title/Abstract]) OR (insomnia[Title/Abstract])) OR (sleeplessness[Title/Abstract])) OR (rest activity[Title/Abstract])))

**Search Strategy of EMBASE database.**

#1 ‘dexmedetomidine’/exp

#2 dexmedetomidine:ab,ti OR precedex:ab,ti

#3 #1 OR #2

#4 ‘surgery’/exp

#5 surger*:ab,ti OR surgical:ab,ti OR operat*:ab,ti OR perioperat*:ab,ti OR postoperat*:ab,ti

#6 #4 OR #5

#7 ‘sleep’/exp

#8 sleep*:ab,ti OR insomnia:ab,ti OR sleeplessness:ab,ti OR ‘rest activity’:ab,ti

#9 #7 OR #8

#10 #3 AND #6 AND #9
