# Supplementary material for: Effect of perioperative dexmedetomidine on sleep quality in adult patients after noncardiac surgery: A systematic review and meta-analysis of randomized trials
Source: PLoS One. 2024 Dec 5;19(12):e0314814. doi: 10.1371/journal.pone.0314814 (PMC11620464; doi:10.1371/journal.pone.0314814)
Supplement: S3 File — (PDF) [file pone.0314814.s003.pdf]

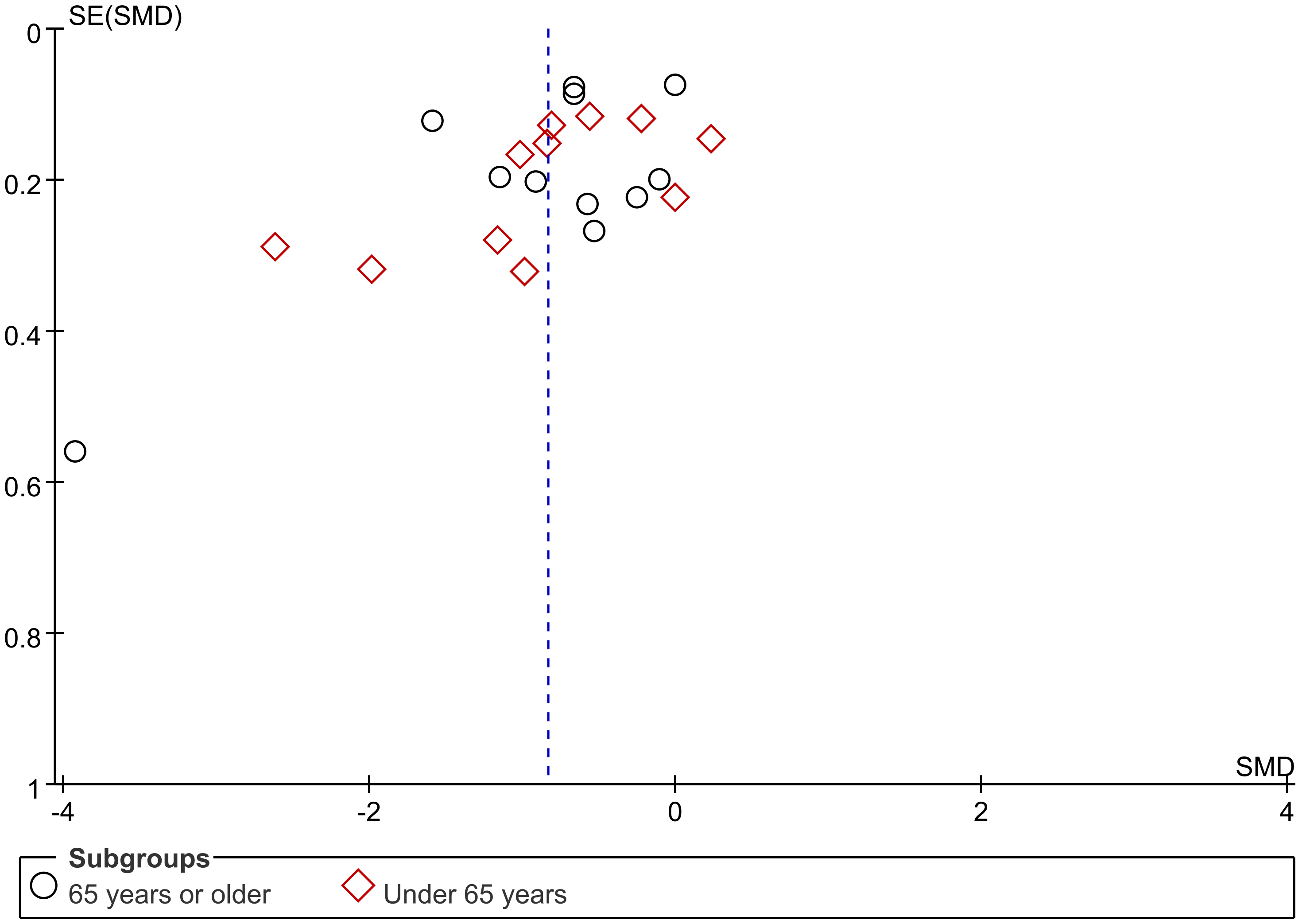

S1 Fig. Funnel plot for subjective sleep score on the first night after surgery.

(A)

Study or Subgroup	Dexmedetomidine Mean	SD	Total	Control Mean	SD	Total	Weight	Mean Difference IV, Random, 95% CI
Chen Z, 2017	65.0	5.0	30	42.0	5.0	29	15.3%	23.00 [20.45, 25.55]
Huang J, 2023	78.3	11.1	17	78.2	14.4	18	14.0%	0.10 [-8.39, 8.59]
Jiang Z, 2018	65.2	10.3	65	41.1	4.2	32	15.3%	24.10 [21.20, 27.00]
Sun YM, 2022	48.3	25.9	33	34.0	25.9	35	12.7%	14.30 [1.98, 26.62]
Wu XH, 2016	22.4	17.0	31	15.0	13.6	30	14.2%	7.40 [-0.31, 15.11]
Wu Y, 2022	66.7	3.3	48	65.2	3.7	48	15.4%	1.50 [0.10, 2.90]
Zhang ZF, 2022	53.0	15.6	44	37.0	33.3	41	13.1%	16.00 [4.81, 27.19]
**Total (95% CI)**			**268**			**233**	**100.0%**	**12.41 [1.96, 22.85]**

Heterogeneity: $Tau^2 = 184.11$; $Chi^2 = 336.45$, df = 6 ($P < 0.00001$); $I^2 = 98\%$
Test for overall effect: Z = 2.33 (P = 0.02)

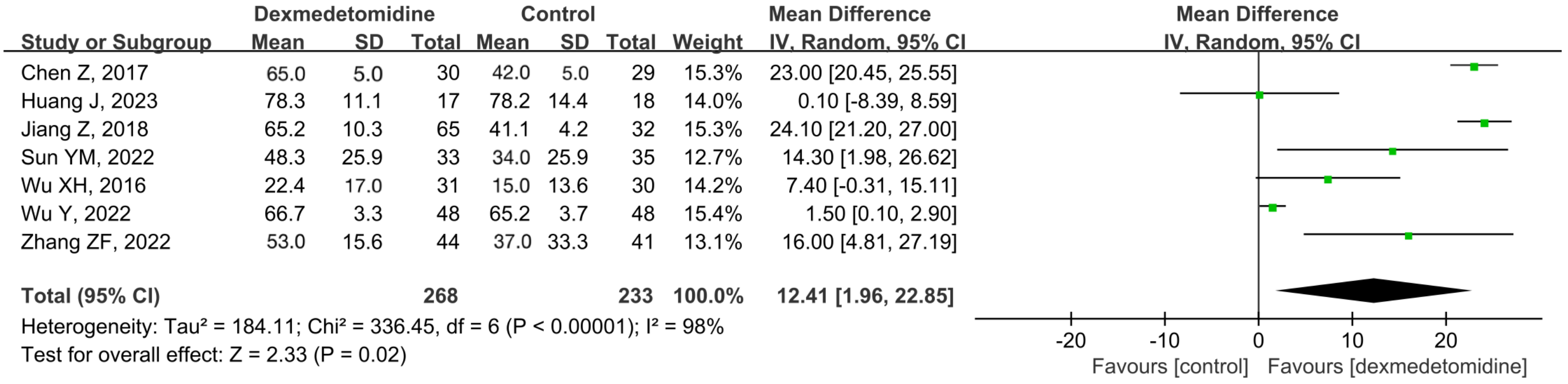

(B)

Study or Subgroup	Dexmedetomidine Mean	SD	Total	Control Mean	SD	Total	Weight	Mean Difference IV, Random, 95% CI
Chen Z, 2017	7.2	1.5	30	12.0	2.1	29	19.4%	-4.80 [-5.73, -3.87]
Huang J, 2023	9.7	7.1	17	14.8	10.0	18	7.1%	-5.10 [-10.82, 0.62]
Jiang Z, 2018	6.5	1.2	65	11.0	2.2	32	19.6%	-4.50 [-5.32, -3.68]
Sun YM, 2022	2.7	2.2	33	3.6	3.6	35	18.3%	-0.90 [-2.31, 0.51]
Wu Y, 2022	7.0	1.0	48	8.0	1.5	48	20.0%	-1.00 [-1.51, -0.49]
Zhang ZF, 2022	5.0	3.9	44	6.0	6.5	41	15.6%	-1.00 [-3.30, 1.30]
**Total (95% CI)**			**237**			**203**	**100.0%**	**-2.69 [-4.58, -0.81]**

Heterogeneity: $Tau^2 = 4.57$; $Chi^2 = 86.29$, df = 5 ($P < 0.00001$); $I^2 = 94\%$
Test for overall effect: Z = 2.80 (P = 0.005)

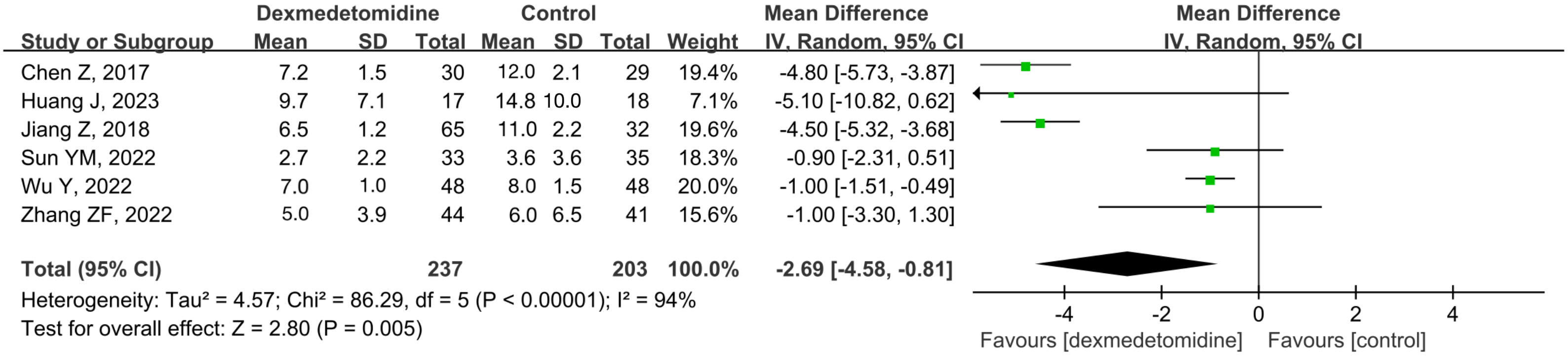

S2 Fig. Forest plot of the pooled analysis showing sleep efficiency index (SEI, %; A) and arousal index (AI, times/h; B) on the first night after surgery.

(A)

Study or Subgroup	Dexmedetomidine Mean	Dexmedetomidine SD	Dexmedetomidine Total	Control Mean	Control SD	Control Total	Weight	Mean Difference IV, Random, 95% CI
Chen Z, 2017	16.4	3.2	30	38.6	4.1	29	20.4%	-22.20 [-24.08, -20.32]
Huang J, 2023	4.1	3.3	17	5.4	5.0	18	20.1%	-1.30 [-4.09, 1.49]
Jiang Z, 2018	18.5	3.0	65	33.0	4.4	32	20.4%	-14.50 [-16.19, -12.81]
Sun YM, 2022	5.6	7.9	33	12.1	18.7	35	17.9%	-6.50 [-13.26, 0.26]
Wu XH, 2016	56.4	51.9	31	84.2	52.2	30	6.2%	-27.80 [-53.93, -1.67]
Zhang ZF, 2022	24.0	16.3	44	33.0	30.4	41	15.0%	-9.00 [-19.48, 1.48]
**Total (95% CI)**			**220**			**185**	**100.0%**	**-11.99 [-19.78, -4.19]**

Heterogeneity: Tau² = 76.70; Chi² = 157.30, df = 5 (P < 0.00001); I² = 97%
Test for overall effect: Z = 3.01 (P = 0.003)

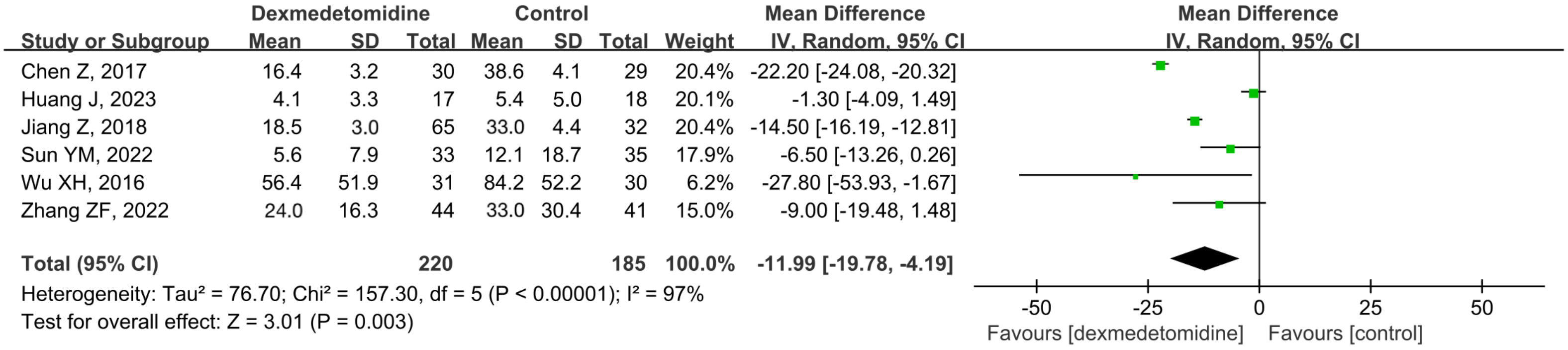

(B)

Study or Subgroup	Dexmedetomidine Mean	Dexmedetomidine SD	Dexmedetomidine Total	Control Mean	Control SD	Control Total	Weight	Mean Difference IV, Random, 95% CI
Chen Z, 2017	68.0	5.6	30	46.5	4.6	29	20.7%	21.50 [18.89, 24.11]
Huang J, 2023	76.8	8.5	17	75.3	7.0	18	19.4%	1.50 [-3.68, 6.68]
Jiang Z, 2018	60.0	3.5	65	35.0	3.7	32	21.0%	25.00 [23.46, 26.54]
Sun YM, 2022	89.9	17.9	33	84.5	20.5	35	16.4%	5.40 [-3.73, 14.53]
Wu XH, 2016	43.5	47.1	31	15.8	45.6	30	7.4%	27.70 [4.44, 50.96]
Zhang ZF, 2022	74.0	16.3	44	66.0	31.1	41	15.2%	8.00 [-2.67, 18.67]
**Total (95% CI)**			**220**			**185**	**100.0%**	**14.12 [6.29, 21.96]**

Heterogeneity: Tau² = 75.47; Chi² = 94.47, df = 5 (P < 0.00001); I² = 95%
Test for overall effect: Z = 3.54 (P = 0.0004)

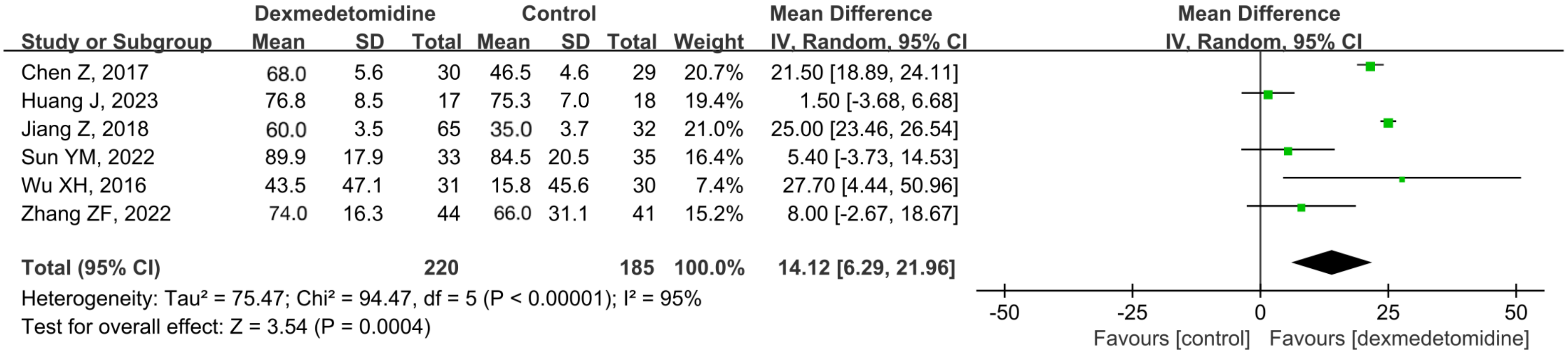

(C)

Study or Subgroup	Dexmedetomidine Mean	Dexmedetomidine SD	Dexmedetomidine Total	Control Mean	Control SD	Control Total	Weight	Mean Difference IV, Random, 95% CI
Chen Z, 2017	10.0	2.3	30	8.8	1.6	29	52.6%	1.20 [0.19, 2.21]
Jiang Z, 2018	10.0	3.6	65	12.0	4.4	32	47.4%	-2.00 [-3.76, -0.24]
**Total (95% CI)**			**95**			**61**	**100.0%**	**-0.32 [-3.45, 2.82]**

Heterogeneity: Tau² = 4.59; Chi² = 9.58, df = 1 (P = 0.002); I² = 90%
Test for overall effect: Z = 0.20 (P = 0.84)

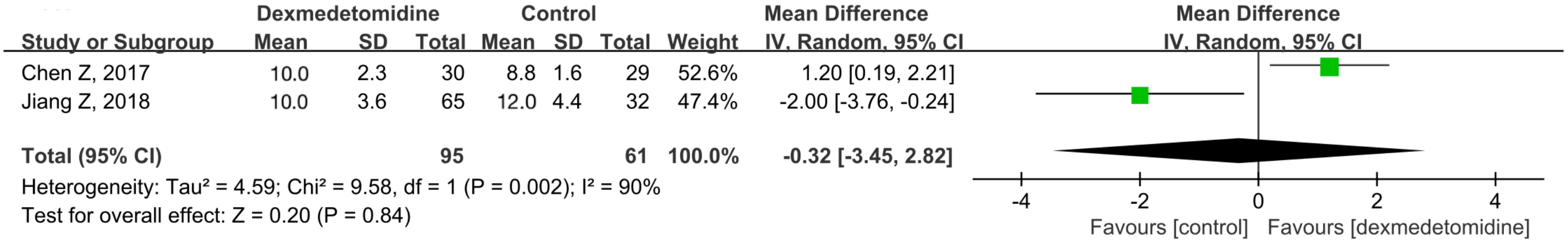

(D)

Study or Subgroup	Dexmedetomidine Mean	Dexmedetomidine SD	Dexmedetomidine Total	Control Mean	Control SD	Control Total	Weight	Mean Difference IV, Random, 95% CI
Chen Z, 2017	5.0	1.6	30	5.0	1.6	29	23.7%	0.00 [-0.82, 0.82]
Huang J, 2023	15.5	5.6	17	17.3	6.6	18	6.6%	-1.80 [-5.85, 2.25]
Jiang Z, 2018	10.5	3.1	65	15.0	3.7	32	18.9%	-4.50 [-5.99, -3.01]
Sun YM, 2022	0.0	0.1	33	0.0	0.1	35	26.7%	0.00 [-0.05, 0.05]
Wu Y, 2022	13.3	1.5	48	12.2	2.2	48	24.1%	1.10 [0.35, 1.85]
**Total (95% CI)**			**193**			**162**	**100.0%**	**-0.70 [-1.90, 0.49]**

Heterogeneity: Tau² = 1.39; Chi² = 44.13, df = 4 (P < 0.00001); I² = 91%
Test for overall effect: Z = 1.15 (P = 0.25)

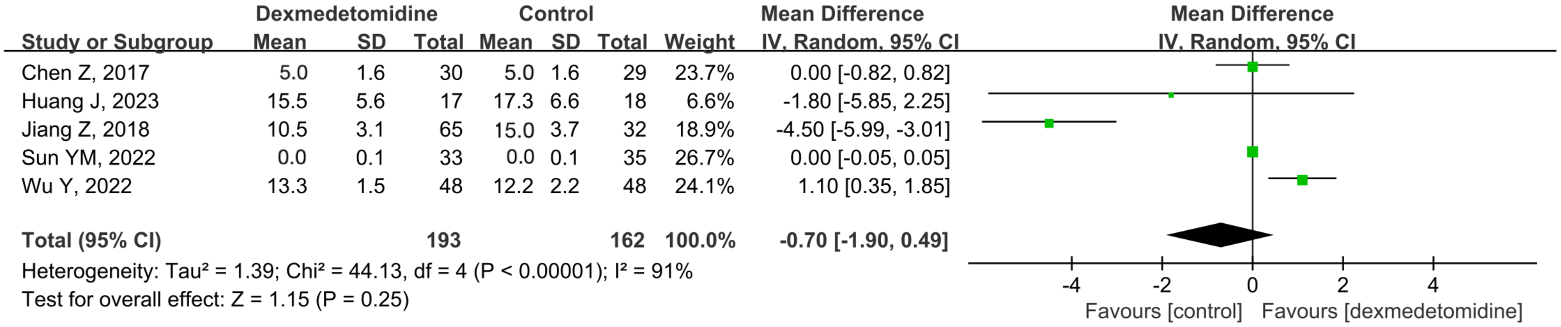

S3 Fig. Forest plot of the pooled analysis showing percentages of stage 1 of non-rapid eye movement sleep (A), stage 2 of non-rapid eye movement sleep (B), stage 3 of non-rapid eye movement sleep (C), and rapid eye movement sleep (D) on the first night after surgery.

Study or Subgroup	Dexmedetomidine Mean	SD	Total	Control Mean	SD	Total	Weight	Std. Mean Difference IV, Random, 95% CI
Chen Z, 2017	2.5	1.2	30	5.0	1.5	30	5.4%	-1.82 [-2.42, -1.21]
Jiang Z, 2018	2.7	1.0	65	5.0	0.6	32	5.7%	-2.57 [-3.13, -2.00]
Li HJ, 2018	2.0	1.5	28	3.0	0.7	29	5.8%	-0.85 [-1.39, -0.30]
Li S, 2023	22.0	10.4	130	30.0	11.9	130	7.6%	-0.71 [-0.96, -0.46]
Lu Y, 2021	1.0	2.2	344	1.0	0.7	331	8.0%	0.00 [-0.15, 0.15]
Shi J, 2022	8.6	2.0	142	9.7	2.1	143	7.7%	-0.53 [-0.77, -0.30]
Sui X, 2022	3.2	2.4	140	4.3	2.9	70	7.4%	-0.43 [-0.72, -0.14]
Sun Y, 2019	36.1	19.0	281	45.2	18.2	276	7.9%	-0.49 [-0.66, -0.32]
Su X, 2016	2.0	3.0	350	4.0	3.0	350	8.0%	-0.67 [-0.82, -0.51]
Ting H, 2019	2.0	2.2	173	4.0	1.5	173	7.7%	-1.06 [-1.29, -0.83]
Wu XH, 2016	3.0	4.4	38	4.0	3.7	38	6.4%	-0.24 [-0.69, 0.21]
Yu HY, 2019	5.5	3.2	281	7.7	2.8	276	7.9%	-0.73 [-0.90, -0.56]
Yu Y, 2023	2.0	1.5	156	2.0	1.5	154	7.7%	0.00 [-0.22, 0.22]
Zhang ZF, 2022	3.3	1.6	50	3.6	1.9	51	6.8%	-0.17 [-0.56, 0.22]
**Total (95% CI)**			**2208**			**2083**	**100.0%**	**-0.68 [-0.92, -0.44]**

Heterogeneity: Tau² = 0.18; Chi² = 175.24, df = 13 (P < 0.00001); I² = 93%
Test for overall effect: Z = 5.54 (P < 0.00001)

Std. Mean Difference
IV, Random, 95% CI

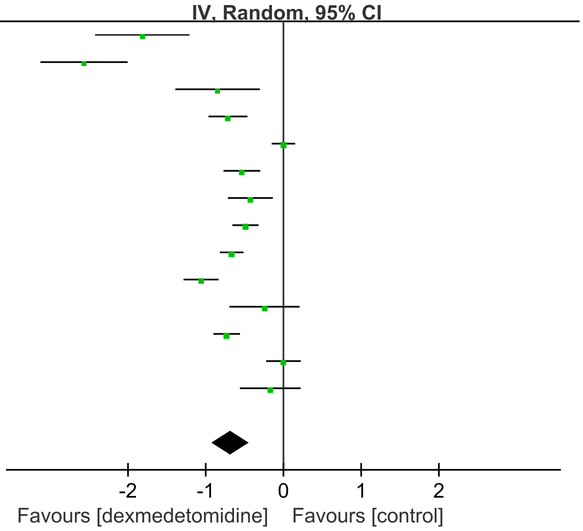

S4 Fig. Forest plot of the pooled analysis showing subjective sleep score on the second night after surgery.

Study or Subgroup	Dexmedetomidine Mean	SD	Total	Control Mean	SD	Total	Weight	Std. Mean Difference IV, Random, 95% CI
Dong YS, 2024	4.5	1.9	94	4.5	2.6	94	9.8%	0.00 [-0.29, 0.29]
Li S, 2023	18.0	10.4	130	24.0	8.9	130	10.2%	-0.62 [-0.87, -0.37]
Lu Y, 2021	1.0	1.5	344	1.0	0.7	331	11.0%	0.00 [-0.15, 0.15]
Sui X, 2022	3.3	2.6	140	5.2	3.7	70	9.7%	-0.63 [-0.92, -0.34]
Sun Y, 2019	31.6	16.3	281	41.5	17.9	276	10.9%	-0.58 [-0.75, -0.41]
Su X, 2016	2.0	3.0	350	4.0	2.2	350	11.0%	-0.76 [-0.91, -0.61]
Ting H, 2019	3.0	1.5	173	3.0	1.5	173	10.5%	0.00 [-0.21, 0.21]
Wu XH, 2016	3.0	3.0	38	4.0	3.0	38	7.9%	-0.33 [-0.78, 0.12]
Yu Y, 2023	2.0	0.7	156	2.0	1.5	154	10.4%	0.00 [-0.22, 0.22]
Zhang ZF, 2022	2.8	1.6	50	2.9	1.6	51	8.6%	-0.06 [-0.45, 0.33]
**Total (95% CI)**			**1756**			**1667**	**100.0%**	**-0.30 [-0.53, -0.08]**

Heterogeneity: Tau² = 0.11; Chi² = 90.59, df = 9 (P < 0.00001); I² = 90%

Test for overall effect: Z = 2.62 (P = 0.009)

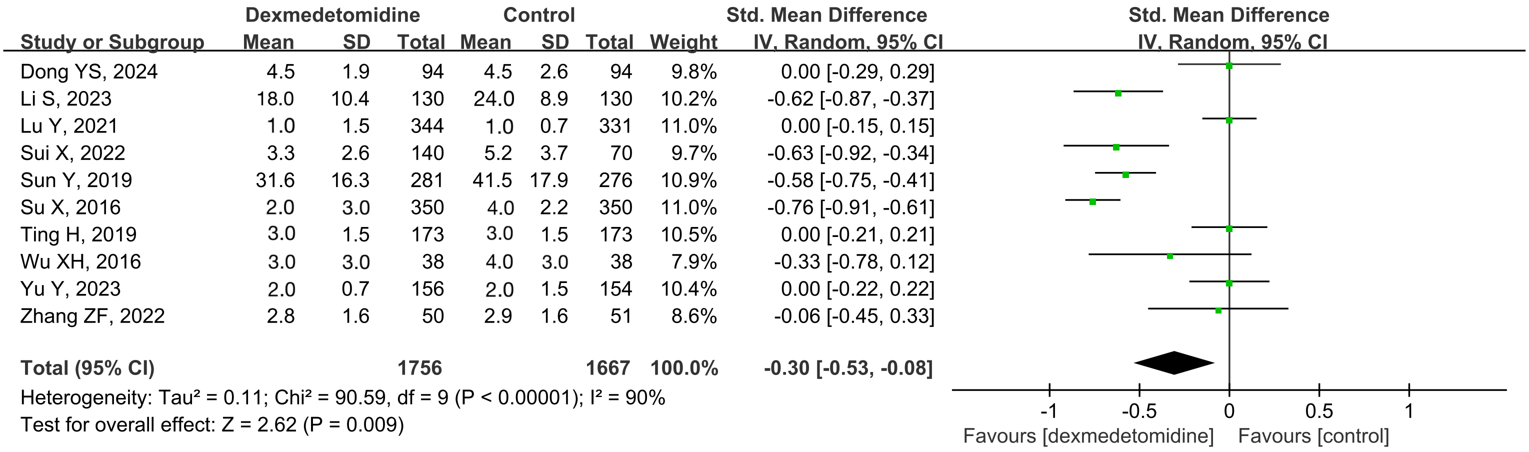

S5 Fig. Forest plot of the pooled analysis showing subjective sleep score on the third night after surgery.

Study or Subgroup	Dexmedetomidine Mean	SD	Total	Control Mean	SD	Total	Weight	Std. Mean Difference IV, Random, 95% CI
Dong YS, 2024	5.5	1.2	94	5.0	1.5	94	14.5%	0.37 [0.08, 0.65]
Liu X, 2020	6.0	1.5	38	7.8	1.5	37	12.8%	-1.19 [-1.68, -0.69]
Lu Y, 2021	0.0	0.7	344	0.0	0.7	331	15.2%	0.00 [-0.15, 0.15]
Shi J, 2022	4.7	1.3	142	6.4	1.6	143	14.7%	-1.16 [-1.41, -0.91]
Sui X, 2022	2.0	1.9	140	3.5	2.9	70	14.4%	-0.66 [-0.95, -0.36]
Sun YM, 2022	8.0	5.2	40	7.0	5.2	40	13.3%	0.19 [-0.25, 0.63]
Yu HY, 2019	4.2	2.6	281	3.9	2.2	276	15.1%	0.12 [-0.04, 0.29]
**Total (95% CI)**			**1079**			**991**	**100.0%**	**-0.32 [-0.74, 0.10]**

Heterogeneity: $Tau^2 = 0.30$; $Chi^2 = 119.57$, df = 6 ($P < 0.00001$); $I^2 = 95\%$

Test for overall effect: Z = 1.49 (P = 0.14)

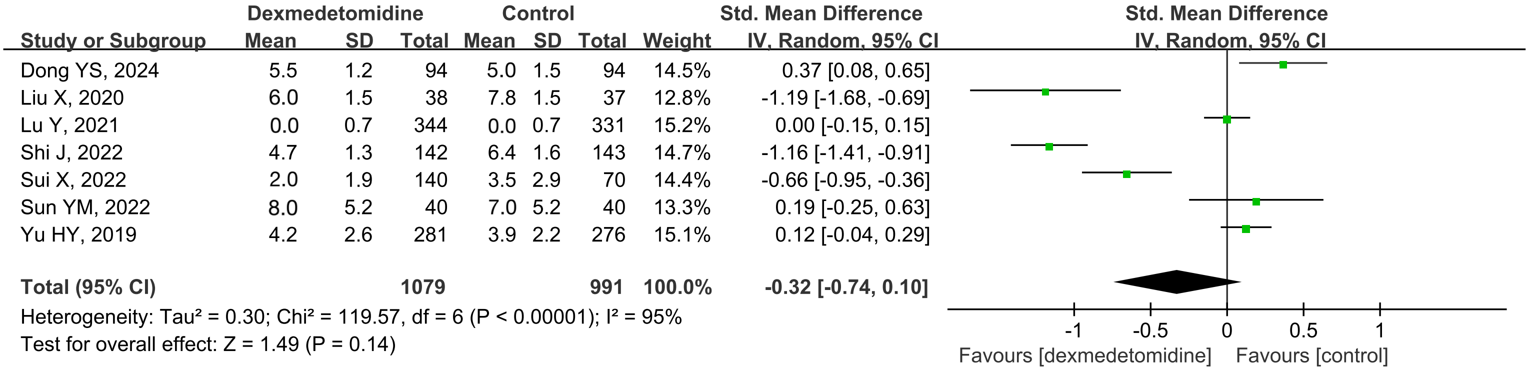

S6 Fig. Forest plot of the pooled analysis showing subjective sleep score at one week or later after surgery.

Study or Subgroup	Dexmedetomidine Mean	Dexmedetomidine SD	Dexmedetomidine Total	Control Mean	Control SD	Control Total	Weight	Mean Difference IV, Random, 95% CI
Chen C, 2016	2.8	0.3	30	3.2	0.3	30	5.7%	-0.40 [-0.55, -0.25]
Chen Z, 2017	1.3	1.3	30	3.0	2.2	29	2.7%	-1.70 [-2.63, -0.77]
Huang J, 2023	1.0	0.0	20	2.0	0.0	20		Not estimable
Li HJ, 2018	2.0	2.2	28	4.0	1.5	29	2.5%	-2.00 [-2.98, -1.02]
Li S, 2023	2.0	0.7	130	3.0	1.5	130	5.3%	-1.00 [-1.28, -0.72]
Liu T, 2022	1.2	0.4	60	1.5	0.5	60	5.6%	-0.30 [-0.46, -0.14]
Liu X, 2020	5.1	0.1	38	6.3	0.3	37	5.8%	-1.20 [-1.30, -1.10]
Lu Y, 2021	3.0	0.7	344	3.0	0.7	331	5.8%	0.00 [-0.11, 0.11]
Mao Y, 2020	2.0	1.5	29	2.0	0.7	29	3.9%	0.00 [-0.60, 0.60]
Qin M, 2017	1.0	0.4	29	2.0	0.7	29	5.2%	-1.00 [-1.29, -0.71]
Shi H, 2020	0.0	0.1	53	0.1	0.3	53	5.8%	-0.10 [-0.19, -0.01]
Sui X, 2022	1.0	2.6	140	1.0	3.0	70	3.0%	0.00 [-0.82, 0.82]
Sun Y, 2019	1.0	0.7	281	2.0	1.5	276	5.6%	-1.00 [-1.19, -0.81]
Su X, 2016	1.0	1.5	350	1.0	2.2	350	5.3%	0.00 [-0.28, 0.28]
Tan W(2), 2016	4.0	2.2	55	4.5	0.7	53	3.9%	-0.50 [-1.11, 0.11]
Ting H, 2019	3.0	2.2	173	4.0	2.2	173	4.5%	-1.00 [-1.46, -0.54]
Wu Y, 2022	2.6	0.2	48	2.8	0.2	48	5.8%	-0.20 [-0.28, -0.12]
Xu S, 2023	1.0	0.7	80	1.5	0.7	80	5.5%	-0.50 [-0.72, -0.28]
Yang X, 2015	4.0	2.2	39	4.0	1.5	40	3.0%	0.00 [-0.83, 0.83]
Yu HY, 2019	1.8	0.8	281	2.3	0.6	276	5.7%	-0.50 [-0.62, -0.38]
Yu Y, 2023	2.9	1.4	156	3.2	1.3	154	5.2%	-0.30 [-0.60, 0.00]
Zhang ZF, 2022	2.0	1.5	58	1.0	1.5	59	4.2%	1.00 [0.46, 1.54]
**Total (95% CI)**			**2452**			**2356**	**100.0%**	**-0.48 [-0.69, -0.27]**

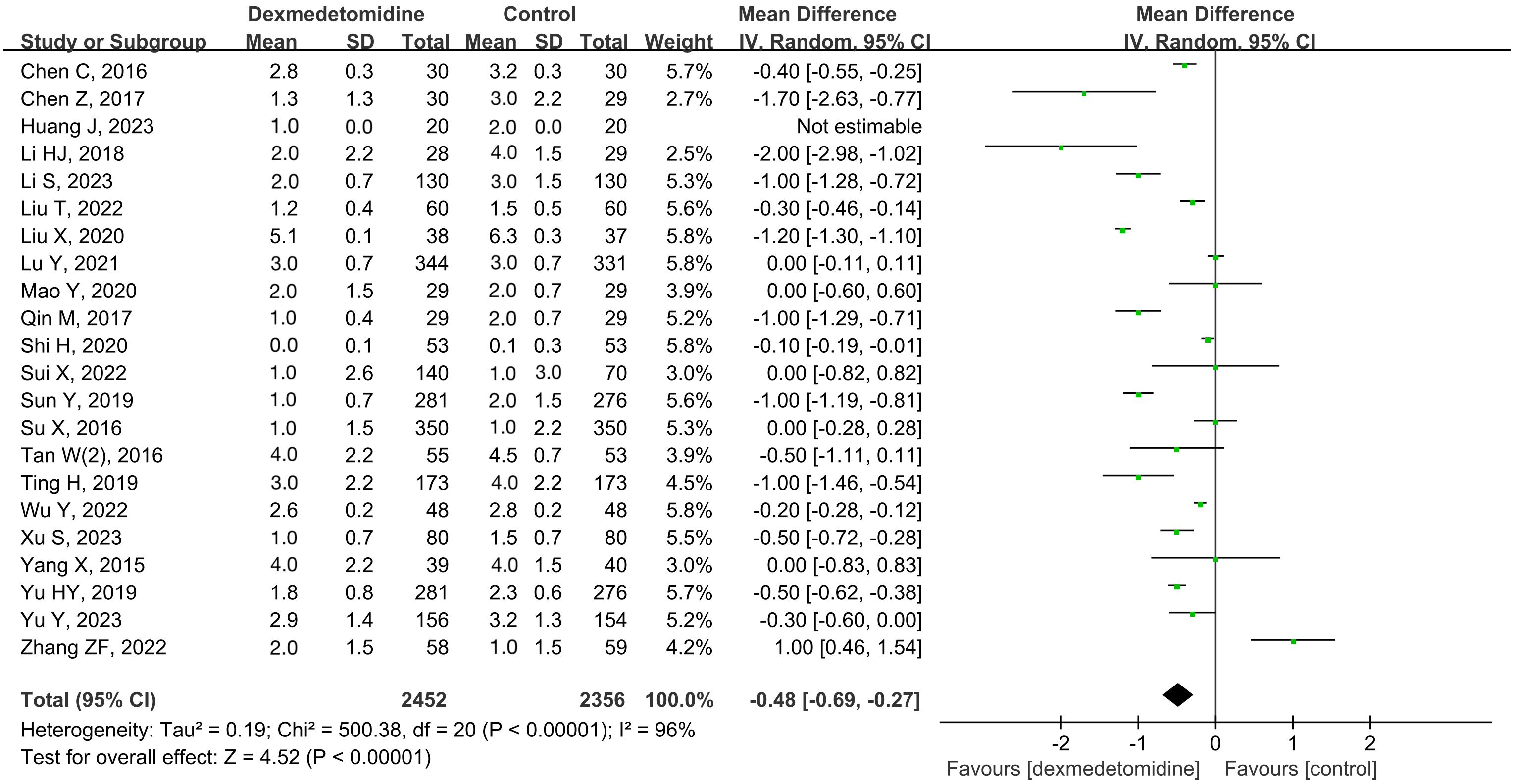

Heterogeneity: $Tau^2 = 0.19$; $Chi^2 = 500.38$, df = 20 ($P < 0.00001$); $I^2 = 96\%$
Test for overall effect: Z = 4.52 ($P < 0.00001$)

S7 Fig. Forest plot of the pooled analysis showing numeric rating scale of pain at 24 h after surgery.

Study or Subgroup	Dexmedetomidine Mean	SD	Total	Control Mean	SD	Total	Weight	Mean Difference IV, Random, 95% CI
Chen C, 2016	103.4	4.6	30	121.6	8.7	30	8.1%	-18.20 [-21.72, -14.68]
Chen Z, 2017	68.0	6.5	30	87.5	7.5	29	8.0%	-19.50 [-23.09, -15.91]
Huang J, 2023	36.8	1.2	20	41.7	1.0	20	9.1%	-4.90 [-5.58, -4.22]
Kang RA. 2019	18.9	10.2	22	27.1	12.5	22	6.1%	-8.20 [-14.94, -1.46]
Li HJ, 2018	39.0	2.7	28	49.0	3.3	29	8.9%	-10.00 [-11.56, -8.44]
Mao Y, 2020	1.3	3.8	29	1.1	2.6	29	8.9%	0.20 [-1.48, 1.88]
Qin M, 2017	38.0	1.8	29	47.8	4.7	29	8.8%	-9.80 [-11.63, -7.97]
Sui X, 2022	94.4	16.3	140	99.6	19.8	70	7.0%	-5.20 [-10.57, 0.17]
Su X, 2016	4.0	3.7	350	3.5	3.0	350	9.1%	0.50 [0.00, 1.00]
Wu XH, 2016	8.1	4.5	38	8.0	3.1	38	8.8%	0.10 [-1.64, 1.84]
Yang X, 2015	5.1	6.3	39	6.5	6.8	40	8.4%	-1.40 [-4.29, 1.49]
Zhang ZF, 2022	37.0	5.2	58	37.0	5.2	59	8.8%	0.00 [-1.88, 1.88]
**Total (95% CI)**			**813**			**745**	**100.0%**	**-6.14 [-9.05, -3.23]**

Heterogeneity: Tau² = 24.17; Chi² = 526.69, df = 11 (P < 0.00001); I² = 98%
Test for overall effect: Z = 4.13 (P < 0.0001)

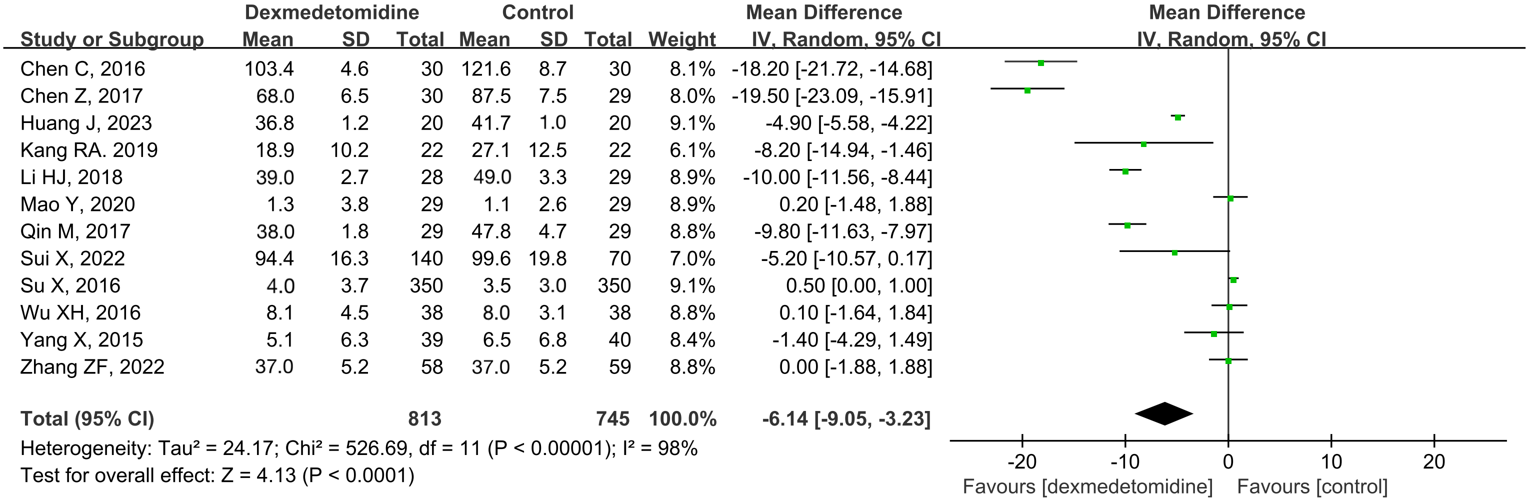

S8 Fig. Forest plot of the pooled analysis showing morphine equivalent consumption within 7 days after surgery.

Study or Subgroup	Dexmedetomidine Events	Dexmedetomidine Total	Control Events	Control Total	Weight	Risk Ratio M-H, Random, 95% CI
Li HJ, 2018	0	28	2	29	0.7%	0.21 [0.01, 4.13]
Li S, 2023	28	130	60	130	18.1%	0.47 [0.32, 0.68]
Lu Y, 2021	41	344	43	331	17.3%	0.92 [0.61, 1.37]
Mao Y, 2020	0	29	1	29	0.7%	0.33 [0.01, 7.86]
Shi H, 2020	4	53	6	53	4.1%	0.67 [0.20, 2.23]
Sun Y, 2019	33	281	38	276	16.1%	0.85 [0.55, 1.32]
Sun YM, 2022	6	40	5	40	4.7%	1.20 [0.40, 3.62]
Su X, 2016	32	350	79	350	17.9%	0.41 [0.28, 0.59]
Wu XH, 2016	2	38	3	38	2.1%	0.67 [0.12, 3.77]
Wu Y, 2022	2	48	4	48	2.3%	0.50 [0.10, 2.60]
Yang X, 2015	2	39	5	40	2.5%	0.41 [0.08, 1.99]
Yu Y, 2023	20	156	23	154	12.6%	0.86 [0.49, 1.50]
Zhang ZF, 2022	2	58	0	59	0.7%	5.08 [0.25, 103.68]
**Total (95% CI)**		**1594**		**1577**	**100.0%**	**0.66 [0.50, 0.85]**
Total events	172		269			

Heterogeneity: Tau² = 0.06; Chi² = 18.18, df = 12 (P = 0.11); I² = 34%
Test for overall effect: Z = 3.15 (P = 0.002)

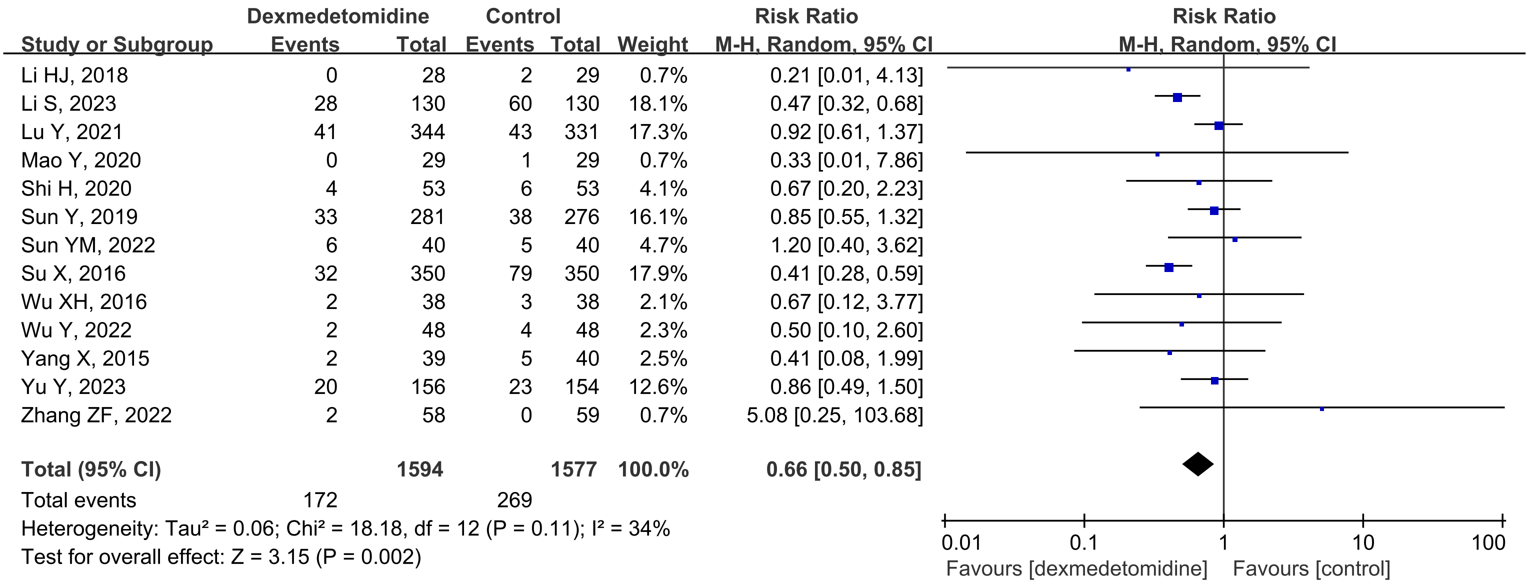

S9 Fig. Forest plot of the pooled analysis showing incidence of delirium after surgery.

(A)

Study or Subgroup	Dexmedetomidine Events	Dexmedetomidine Total	Control Events	Control Total	Weight	Risk Ratio M-H, Random, 95% CI
Jiang Z, 2018	9	65	4	32	5.3%	1.11 [0.37, 3.32]
Li S, 2023	5	130	3	130	3.5%	1.67 [0.41, 6.83]
Liu T, 2022	8	60	4	60	5.0%	2.00 [0.64, 6.29]
Shi H, 2020	26	53	13	53	13.0%	2.00 [1.16, 3.45]
Shi J, 2022	5	142	9	143	5.5%	0.56 [0.19, 1.63]
Sun Y, 2019	26	281	14	276	11.3%	1.82 [0.97, 3.42]
Sun YM, 2022	4	33	0	35	0.9%	9.53 [0.53, 170.42]
Su X, 2016	59	350	46	350	17.9%	1.28 [0.90, 1.83]
Wu XH, 2016	12	38	6	38	7.5%	2.00 [0.84, 4.78]
Xu S, 2023	58	80	18	80	15.9%	3.22 [2.10, 4.95]
Yang X, 2015	1	39	0	40	0.8%	3.08 [0.13, 73.27]
Yu Y, 2023	28	156	20	154	13.4%	1.38 [0.81, 2.35]
**Total (95% CI)**		**1427**		**1391**	**100.0%**	**1.72 [1.29, 2.28]**
Total events	241		137			

Heterogeneity: Tau² = 0.08; Chi² = 18.38, df = 11 (P = 0.07); I² = 40%
Test for overall effect: Z = 3.71 (P = 0.0002)

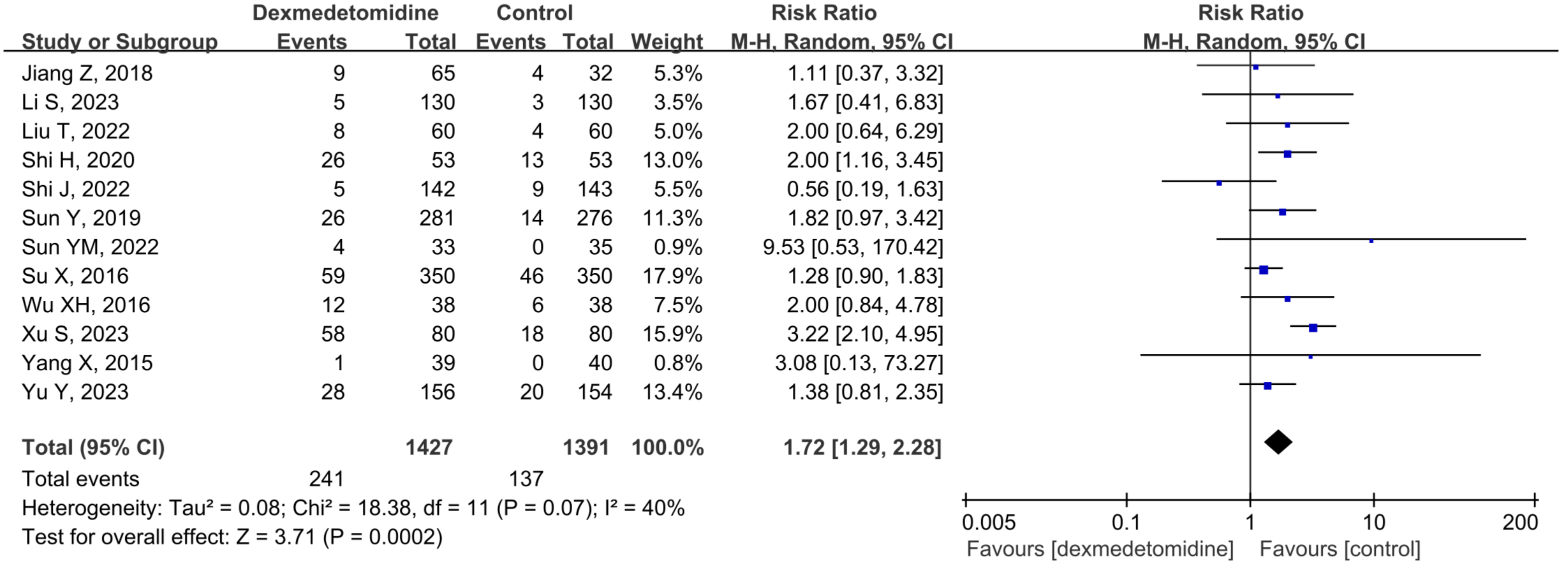

(B)

Study or Subgroup	Dexmedetomidine Events	Dexmedetomidine Total	Control Events	Control Total	Weight	Risk Ratio M-H, Random, 95% CI
Jiang Z, 2018	10	65	2	32	2.0%	2.46 [0.57, 10.58]
Li HJ, 2018	1	28	2	29	0.8%	0.52 [0.05, 5.40]
Li S, 2023	11	130	9	130	5.8%	1.22 [0.52, 2.85]
Liu T, 2022	3	60	1	60	0.9%	3.00 [0.32, 28.03]
Shi H, 2020	6	53	4	53	3.0%	1.50 [0.45, 5.01]
Shi J, 2022	2	142	11	143	2.0%	0.18 [0.04, 0.81]
Sun Y, 2019	4	281	2	276	1.5%	1.96 [0.36, 10.64]
Sun YM, 2022	1	33	2	35	0.8%	0.53 [0.05, 5.58]
Su X, 2016	114	350	92	350	46.0%	1.24 [0.98, 1.56]
Wu XH, 2016	15	38	5	38	5.1%	3.00 [1.21, 7.43]
Xu S, 2023	19	80	13	80	10.0%	1.46 [0.78, 2.75]
Yang X, 2015	0	39	1	40	0.4%	0.34 [0.01, 8.14]
Yu Y, 2023	39	156	32	154	20.9%	1.20 [0.80, 1.81]
Zhang ZF, 2022	2	58	1	59	0.8%	2.03 [0.19, 21.83]
**Total (95% CI)**		**1513**		**1479**	**100.0%**	**1.28 [1.04, 1.59]**
Total events	227		177			

Heterogeneity: Tau² = 0.01; Chi² = 13.87, df = 13 (P = 0.38); I² = 6%
Test for overall effect: Z = 2.33 (P = 0.02)

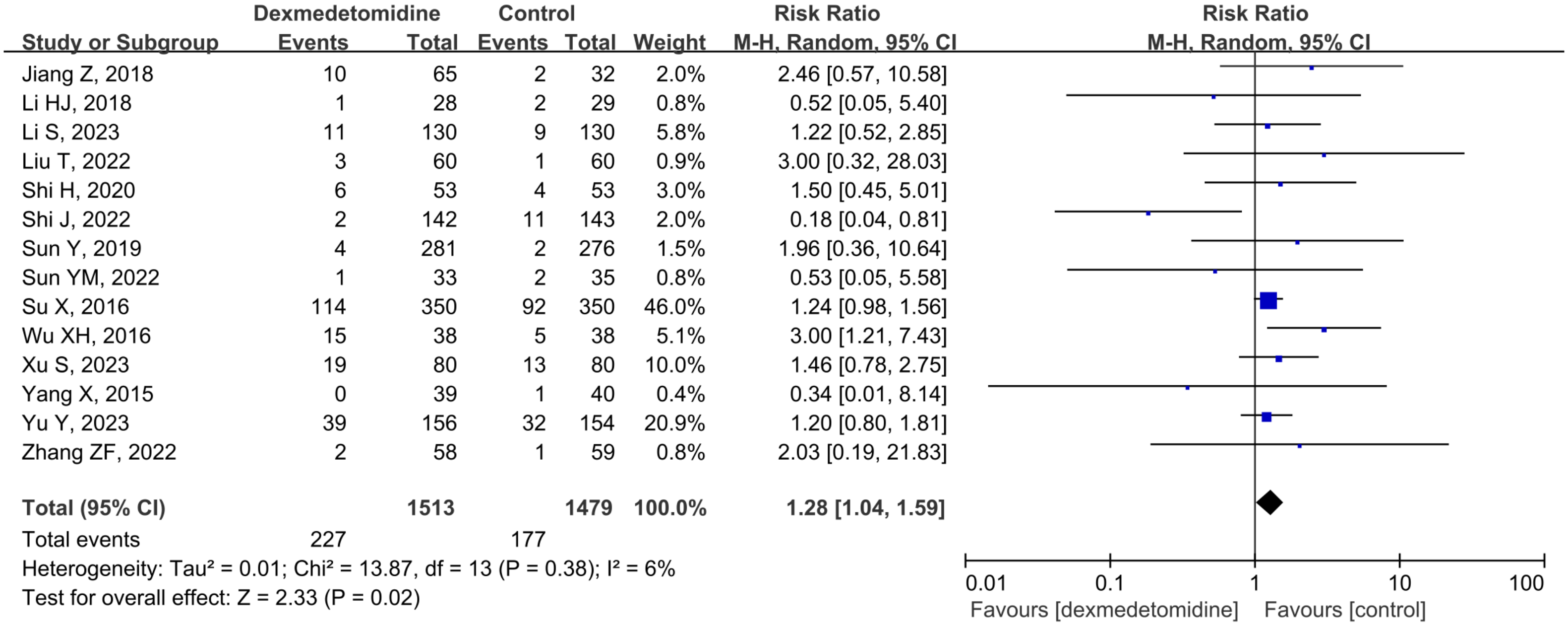

S10 Fig. Forest plot of the pooled analysis showing incidences of bradycardia (A) and hypotension (B).
