# Supplementary material for: Effect of perioperative dexmedetomidine on sleep quality in adult patients after noncardiac surgery: A systematic review and meta-analysis of randomized trials
Source: PLoS One. 2024 Dec 5;19(12):e0314814. doi: 10.1371/journal.pone.0314814 (PMC11620464; doi:10.1371/journal.pone.0314814)
Supplement: S1 Table — (DOCX) [file pone.0314814.s004.docx]

**S1 Table.** All studies identified in the literature search.

| **No.** | **Author, year** | **Title** | **Journal** | **Inclusion/ exclusion** | **Reason for exclusion** |
| --- | --- | --- | --- | --- | --- |
| 1 | Chen C, 2016 | Dexmedetomidine improves gastrointestinal motility after laparoscopic resection of colorectal cancer: A randomized clinical trial | Medicine (Baltimore). 2016; 95:4295 | Included (ref 23) | --- |
| 2 | Chen Z, 2017 | Effects of dexmedetomidine administered for postoperative analgesia on sleep quality in patients undergoing abdominal hysterectomy | J Clin Anesth. 2017; 36:118-122. | Included (ref 24) | --- |
| 3 | Dong YS, 2024 | Effect of dexmedetomidine infusion on post-operative sleep disturbances in women with breast cancer: A monocentric randomized-controlled double-blind trial | Anaesth Crit Care Pain Med. 2024:101358 | Included (ref 25) | --- |
| 4 | Huang J, 2023 | Dexmedetomidine improved sleep quality in the intensive care unit after laryngectomy | Drug Des Devel Ther. 2023; 17:1631-1640 | Included (ref 26) | --- |
| 5 | Jiang Z, 2018 | Effect of intravenous oxycodone in combination with different doses of dexmedetomidine on sleep quality and visceral pain in patients after abdominal surgery a randomized study | Clin J Pain. 2018; 34(12):1126-1132. | Included (ref 28) | --- |
| 6 | Kang RA, 2019 | Improvement in postoperative pain control by combined use of intravenous dexamethasone with intravenous dexmedetomidine after interscalene brachial plexus block for arthroscopic shoulder surgery | Eur J Anaesthesiol. 2019; 36(5):360-368. | Included (ref 29) | --- |
| 7 | Li HJ, 2018 | Dexmedetomidine in combination with morphine improves postoperative analgesia and sleep quality in elderly patients after open abdominal surgery: A pilot randomized control trial | PLoS One. 2018; 13(8):0202008. | Included (ref 30) | --- |
| 8 | Li S, 2023 | Dexmedetomidine administration during brain tumour resection for prevention of postoperative delirium: a randomised trial | Br J Anaesth. 2023; 130(2):307-e316 | Included (ref 31) | --- |
| 9 | Liu T, 2022 | Effect of perioperative dexmedetomidine infusion on postoperative delirium in elderly patients undergoing oral and maxillofacial surgery a randomized controlled clinical trial | Int J Gen Med. 2022; 15:6105-6113. | Included (ref 32) | --- |
| 10 | Liu X, 2020 | Combination of post-fascia iliaca compartment block and dexmedetomidine in pain and inflammation control after total hip arthroplasty for elder patients: a randomized control study | J Orthop Surg Res. 2020; 15(1):42. | Included (ref 33) | --- |
| 11 | Lu Y, 2021 | effect of intraoperative dexmedetomidine on recovery of gastrointestinal function after abdominal surgery in older adults a randomized clinical trial | JAMA Netw Open. 2021; 4(10):e2128886. | Included (ref 34) | --- |
| 12 | Mao Y, 2020 | Perioperative dexmedetomidine fails to improve postoperative analgesic consumption and postoperative recovery in patients undergoing lateral thoracotomy for thoracic esophageal cancer | Pain Res Manag. 2020; 2020:4145893. | Included (ref 35) | --- |
| 13 | Qin M, 2017 | Dexmedetomidine in combination with sufentanil for postoperative analgesia after partial laryngectomy | BMC Anesthesiol. 2017; 17(1):66. | Included (ref 36) | --- |
| 14 | Shi H, 2020 | Dexmedetomidine improves early postoperative neurocognitive disorder in elderly male patients undergoing thoracoscopic lobectomy | Exp Ther Med. 2020; 20(4):3868-3877. | Included (ref 37) | --- |
| 15 | Shi J, 2022 | Effects of dexmedetomidine combined with intravenous anesthesia on oxidative stress index, postoperative sleep quality, and brain function in HICH patients | J Healthc Eng. 2022; 2022:5463986. | Included (ref 38) | --- |
| 16 | Su X, 2016 | Dexmedetomidine for prevention of delirium in elderly patients after non-cardiac surgery: a randomised, double-blind, placebo-controlled trial | J Neur Anesth. 2017; 29(2):178‐179. | Included (ref 14) | --- |
| 17 | Sui X, 2022 | The effects of dexmedetomidine for patient-controlled analgesia on postoperative sleep quality and gastrointestinal motility function after surgery_ A prospective, randomized, double-blind, and controlled trial | Front Pharmacol. 2022; 13:990358. | Included (ref 39) | --- |
| 18 | Sun Y, 2019 | Impact of postoperative dexmedetomidine infusion on incidence of delirium in elderly patients undergoing major elective noncardiac surgery_ a randomized clinical trial | Drug Des Devel Ther. 2019; 13:2911-2922. | Included (ref 40) | --- |
| 19 | Sun YM, 2022 | Effect of low-dose dexmedetomidine on sleep quality in postoperative patients with mechanical ventilation in the intensive care unit: A pilot randomized trial | Front Med (Lausanne). 2022; 9:931084. | Included (ref 41) | --- |
| 20 | Tan W, 2016 | Changes in first postoperative night bispectral index after daytime sedation induced by dexmedetomidine or midazolam under regional anesthesia: a randomized controlled trial | Clin Exp Pharmacol Physiol. 2016; 43(3):304-311. | Included (ref 17) | --- |
| 21 | Tan W, 2016 (2) | Changes in postoperative night bispectral index of patients undergoing thoracic surgery with different types of anaesthesia management: a randomized controlled trial | Reg Anesth Pain Med. 2016; 41(3):380-386. | Included (ref 42) | --- |
| 22 | Ting H, 2019 | Perioperative dexmedetomidine reduces delirium in elderly patients after lung cancer surgery | Psychiatr Danub. 2019; 31(1):95-101. | Included (ref 27) | --- |
| 23 | Wu XH, 2016 | Low-dose dexmedetomidine improves sleep quality pattern in elderly patients after noncardiac surgery in the intensive care unit a pilot randomized controlled trial | Anesthesiol. 2016; 125(5):979-991. | Included (ref 15) | --- |
| 24 | Wu Y, 2022 | A randomized placebo-controlled double-blind study of dexmedetomidine on postoperative sleep quality in patients with endoscopic sinus surgery | BMC Anesthesiol. 2022; 22:172 | Included (ref 43) | --- |
| 25 | Xu S, 2023 | Effect of co-administration of intravenous lidocaine and dexmedetomidine on the recovery from laparoscopic hysterectomy: a randomized controlled trial | Minerva Anestesiol. 2023; 89(1-2):10-21 | Included (ref 44) | --- |
| 26 | Yang X, 2015 | Effect of dexmedetomidine on preventing agitation and delirium after microvascular free flap surgery: a randomized, double-blind, control study | J Oral Maxillofac Surg. 2015; 73(6):1065-1072. | Included (ref 45) | --- |
| 27 | Yu HY, 2019 | Dexmedetomidine alleviates postpartum depressive symptoms following cesarean section in Chinese women: a randomized placebo-controlled study | Pharmacotherapy. 2019; 39(10):994-1004. | Included (ref 46) | --- |
| 28 | Yu Y, 2023 | Effect of dexmedetomidine on posttraumatic stress disorder in patients undergoing emergency trauma surgery a randomized clinical trial | JAMA Netw Open. 2023;6(6):2318611 | Included (ref 47) | --- |
| 29 | Zhang ZF, 2022 | Effect of mini-dose dexmedetomidine supplemented intravenous analgesia on sleep structure in older patients after major noncardiac surgery: A randomized trial | Sleep Med. 2023; 102:9-18 | Included (ref 48) | --- |
| 30 | Alexandra P, 2020 | The effect of regional anaesthesia and sedation using dexmedetomidine on the effects of the anaesthesia after surgery | https://trialsearch.who.int/Trial2.aspx?TrialID=ISRCTN13642102. 2020. | Excluded | Trial protocol |
| 31 | Alexandros M, 2019 | Dexmedetomidine Sedation in Orthopedic Surgery | https://trialsearch.who.int/Trial2.aspx?TrialID=ChiCTR1800016750. 2018. | Excluded | Trial protocol |
| 32 | Azarfarin R, 2021 | Comparison of the effect of dexmedetomidine and propofol on how patients sleep after cardiac surgery | https://trialsearch.who.int/Trial2.aspx?TrialID=ChiCTR-IPR-15006597. 2015. | Excluded | Trial protocol |
| 33 | Bagheri B, 2016 | Comparison of sedative and analgesic effect of Gabapentin and Dexmedetomidine | https://trialsearch.who.int/Trial2.aspx?TrialID=TCTR20210322007. 2021. | Excluded | Trial protocol |
| 34 | Chen F, 2019 | Effect of dexmedetomidine combined with melatonin on postoperative sleep disturbance in patients undergoing laparoscopic hysterectomy | http://www.who.int/trialsearch/Trial2.aspx?TrialID=ChiCTR1900027552. 2019. | Excluded | Trial protocol |
| 35 | Cheng W, 2021 | Effect of dexmedetomidine combined with transcutaneous acupoint electrical stimulation on postoperative sleep quality of patients undergoing laparoscopic gynecological surgery | http://www.who.int/trialsearch/Trial2.aspx?TrialID=ChiCTR1900026989. 2019. | Excluded | Trial protocol |
| 36 | Cheng W, 2021 | Effect of dexmedetomidine combined with sufentanil for intravenous patient-controlled analgesia on sleep quality in elderly patients after knee replacement | https://trialsearch.who.int/Trial2.aspx?TrialID=IRCT2016011822794N2. 2016. | Excluded | Trial protocol |
| 37 | Fang PP, 2018 | Effect of dexmedetomidine on the postoperative gastrointestinal function in the elderly abdominal surgery | https://trialsearch.who.int/Trial2.aspx?TrialID=IRCT20161127031131N3. 2022. | Excluded | Trial protocol |
| 38 | Gao T, 2017 | The Addition of Dexmedetomidine on Postoperative Sleep for Middle-aged and Elderly Patients With Gastric Cancer | https://clinicaltrials.gov/show/NCT03477344. 2018. | Excluded | Trial protocol |
| 39 | Gargadennec T, 2018 | Dexmedetomidine After Cardiac Surgery for Prevention of Delirium | BMJ Open. 2022; 12(4):e058968 | Excluded | Trial protocol |
| 40 | Gargadennec T, 2022 | Dexmedetomidine after Cardiac Surgery for Prevention of Delirium (EXACTUM) trial protocol: a multicentre randomised, double-blind, placebo-controlled trial | https://trialsearch.who.int/Trial2.aspx?TrialID=ChiCTR2100051438. 2021. | Excluded | Trial protocol |
| 41 | Gu XP, 2018 | The Effect on Perioperative Infusion of Dexmedetomidine for Postoperative Cognitive Dysfunction in Elderly Patients undergoing Major Surgery | https://clinicaltrials.gov/show/NCT04149626. 2019. | Excluded | Trial protocol |
| 42 | Han F, 2020 | Effects of different doses of dexmedetomidine on postoperative analgesia, sleep and gastrointestinal function in patients with colorectal cancer | http://www.who.int/trialsearch/Trial2.aspx?TrialID=ChiCTR1900026205. 2019. | Excluded | Trial protocol |
| 43 | Jiang ZM, 2016 | Effects of oxycodone hydrochloride combined dexmedetomidine for postoperative analgesia on sleep quality in patients after major gastrointestinal surgery | https://trialsearch.who.int/Trial2.aspx?TrialID=ChiCTR2100050152. 2021. | Excluded | Trial protocol |
| 44 | Kim JM, 2018 | Effect of Dexmedetomidine on Postoperative Delirium Inflammasome Activation Inhibition | https://trialsearch.who.int/Trial2.aspx?TrialID=ChiCTR2100046894. 2021. | Excluded | Trial protocol |
| 45 | Lertkovit S, 2021 | Analgesic Effect and sleep quality of Intravenous, low-dose, dexmedetomidine infusion in cardiac patients after ultra-fast track endotracheal extubation, A randomized placebo-controlled study | https://clinicaltrials.gov/show/NCT03588988. 2018. | Excluded | Trial protocol |
| 46 | Li Y, 2018 | A prospective, open clinical study for dexmedetomidine combined with butorphanol in the prevention of patient awareness for general anesthesia resuscitation | http://www.who.int/trialsearch/Trial2.aspx?TrialID=ChiCTR2100043594. 2021. | Excluded | Trial protocol |
| 47 | Liu HQ, 2017 | The effects of different doses of intravenous dexmedetomidine continously at night on the circadian rhythm in the elderly gastrointestinal carcinoma patients | https://trialsearch.who.int/Trial2.aspx?TrialID=ChiCTR1800017232. 2018. | Excluded | Trial protocol |
| 48 | Liu Y, 2020 | Effects of Dexmedetomidine on postoperative sleep and cognitive function during abdominal surgery in the elderly: a randomized double-blind controlled trial | https://clinicaltrials.gov/show/NCT00333632. 2006. | Excluded | Trial protocol |
| 49 | Liu Y, 2020 | Effects of dexmedetomidine on postoperative sleep quality and cognitive function: a randomized controlled trial | https://trialsearch.who.int/Trial2.aspx?TrialID=ChiCTR2000033432. 2020. | Excluded | Trial protocol |
| 50 | Lu X, 2021 | Effect of dexmedetomidine on postoperative sleep rhythm in elderly patients under regional anesthesia | http://www.who.int/trialsearch/Trial2.aspx?TrialID=ChiCTR2000028954. 2020. | Excluded | Trial protocol |
| 51 | Ma H, 2012 | Postoperative Sleep Quality in Patients Undergoing Thoracic Surgery With Different Types of Anesthesia Management | https://clinicaltrials.gov/show/NCT05325762. 2022. | Excluded | Trial protocol |
| 52 | Ma H, 2014 | Postoperative Sleep Quality of Patients Sedation With i.v. Dexmedetomidine or Midazolam Undergoing TURP | [https://www.cochranelibrary.com/central/doi/10.1002/central/CN-01707455/full. 2019](https://www.cochranelibrary.com/central/doi/10.1002/central/CN-01707455/full.%202019) | Excluded | Trial protocol |
| 53 | Macleod DB, 2006 | Effect of Dexmedetomidine Upon Sleep Postoperatively | https://trialsearch.who.int/Trial2.aspx?TrialID=ChiCTR2000040698. 2020. | Excluded | Trial protocol |
| 54 | Mao Y, 2018 | the impact of dexmedetomidine in patients undergoing radical resection of esophageal carcinoma | http://www.who.int/trialsearch/Trial2.aspx?TrialID=ChiCTR2100043641. 2021. | Excluded | Trial protocol |
| 55 | Qin M, 2021 | Dexmedetomidine improves sleep quality pattern in elder patients after head and neck tumor surgery: a randomized, double blind, controlled trial | https://trialsearch.who.int/Trial2.aspx?TrialID=ChiCTR2000032601. 2020. | Excluded | Trial protocol |
| 56 | Sun Y, 2018 | Postoperative infusion of dexmedetomidine for prevention of postoperative delirium in elderly patients after non-cardiac surgery | https://trialsearch.who.int/Trial2.aspx?TrialID=ChiCTR-INR-17010315. 2017. | Excluded | Trial protocol |
| 57 | Tan W, 2019 | Application of new serratus anterior muscle block combined with dexmedetomidine and dexamethasone in modified radical mastectomy under ultrasound guidance | https://trialsearch.who.int/Trial2.aspx?TrialID=ChiCTR-TRC-12002567. 2012. | Excluded | Trial protocol |
| 58 | Wang BJ, 2018 | Impact of dexmedetomidine infusion during general anaesthesia on incidence of postoperative delirium in elderly patients after major non-cardiac surgery: study protocol of a randomised, double-blinded and placebo-controlled trial | BMJ Open. 2018; 8(4):e019549 | Excluded | Trial protocol |
| 59 | Wang DX, 2015 | The effect of intraoperative dexmedetomidine on occurrence of postoperative delirium in old patients after non-cardiac major surgery | https://clinicaltrials.gov/show/NCT03117790. 2017. | Excluded | Trial protocol |
| 60 | Wang DX, 2017 | Impact of Dexmedetomidine on Sleep Quality | https://clinicaltrials.gov/show/NCT03335527. 2017. | Excluded | Trial protocol |
| 61 | Wang DX, 2017 | Impacts of Low-Dose Dexmedetomidine on Sleep Quality in Mechanically Ventilated ICU Patients | https://clinicaltrials.gov/show/NCT03624595. 2018. | Excluded | Trial protocol |
| 62 | Wang DX, 2018 | Low-dose Dexmedetomidine and Postoperative Delirium After Cardiac Surgery | https://clinicaltrials.gov/show/NCT05246007. 2022. | Excluded | Trial protocol |
| 63 | Wang DX, 2022 | Nocturnal Low-dose Dexmedetomidine Infusion and Perioperative Sleep Quality | https://trialsearch.who.int/Trial2.aspx?TrialID=ChiCTR1800016788. 2018. | Excluded | Trial protocol |
| 64 | Wang E, 2021 | Effects of dexmedetomidine on sleep quality in elderly patients with sleep disorders after general anesthesia | https://clinicaltrials.gov/show/NCT01725607. 2012. | Excluded | Trial protocol |
| 65 | Wang MD, 2022 | Effect of Propofol on Postoperative Sleep Quality in Elderly Patients With Sleep Disorders | https://clinicaltrials.gov/show/NCT02142595. 2014. | Excluded | Trial protocol |
| 66 | Wu Q, 2020 | Effect of intravertebral anesthesia assisted dexmedetomidine sedation on circadian rhythm in patients undergoing lower limb orthopedic surgery | https://clinicaltrials.gov/show/NCT03117023. 2017. | Excluded | Trial protocol |
| 67 | Wu XH, 2012 | Effects of preventively subsedative dose of dexmedetomidine on the quality of postoperative sleep of elderly critically patients: prospective random controlled trial | https://trialsearch.who.int/Trial2.aspx?TrialID=ChiCTR-IPR-15007654. 2015. | Excluded | Trial protocol |
| 68 | Xu G, 2019 | Application of dexmedetomidine combined with femoral nerve block in knee replacement surgery and their effects on postoperative recovery | https://trialsearch.who.int/Trial2.aspx?TrialID=ChiCTR1800017454. 2018. | Excluded | Trial protocol |
| 69 | Zhong P, 2020 | Effect of dexmedetomidine-assisted sedation on postoperative sleep quality in patients undergoing spinal anesthesia: a randomized controlled trial | https://trialsearch.who.int/Trial2.aspx?TrialID=ChiCTR-IOR-17013478. 2017. | Excluded | Trial protocol |
| 70 | Zhou JC, 2015 | A study of effect of scheduled sedation with dexmedetomidine for new extubated postcardiac surgery patients on their sleep and early mobilization | https://trialsearch.who.int/Trial2.aspx?TrialID=ChiCTR1800015054. 2018. | Excluded | Trial protocol |
| 71 | Chen K, 2019 | Effect of dexmedetomidine nasal administration on the sleep disorders of the patients under general anesthesia on the first day after plastic surgery | https://trialsearch.who.int/Trial2.aspx?TrialID=ChiCTR1900020822. 2019. | Excluded | Not i.v. administration |
| 72 | Fang J, 2021 | Intranasal Premedication Effect of Dexmedetomidine on postoperative delirium in patients with sleep-disordered heart disease: a prospective study | http://www.who.int/trialsearch/Trial2.aspx?TrialID=ChiCTR2100041597. 2021. | Excluded | Not i.v. administration |
| 73 | Ghali AM, 2015 | The Effect of Low-Dose Dexmedetomidine as an Adjuvant to Levobupivacaine in Patients Undergoing Vitreoretinal Surgery Under Sub-Tenon's Block Anesthesia | National Med J China. 2018; 98(10):728-732. | Excluded | Not i.v. administration |
| 74 | Jin X, 2019 | Effects of different administration methods of dexmedetomidine on rapid recovery after knee replacement | https://trialsearch.who.int/Trial2.aspx?TrialID=ChiCTR2100044747. 2021. | Excluded | Not i.v. administration |
| 75 | Sane S, 2019 | Evaluation the effects of Bupivacaine and dexmedetomidine on postoperative functional and pain | [https://trialsearch.who.int/Trial2.aspx?TrialID=ChiCTR2100046976. 2021.](https://trialsearch.who.int/Trial2.aspx?TrialID=ChiCTR2100046976.%202021.) | Excluded | Not i.v. administration |
| 76 | Wang XL, 2018 | Dexmedetomidine combined with ropivacaine for continuous femoral nerve block improved postoperative sleep quality in elderly patients after total knee arthroplasty | http://www.who.int/trialsearch/Trial2.aspx?TrialID=ChiCTR1900025808. 2019. | Excluded | Not i.v. administration |
| 77 | Wang Y, 2021 | Clinical observation of dexmedetomidine nasal spray in the treatment of postoperative sleep disorders | J Robot Surg. 2024; 18(1):35 | Excluded | Not i.v. administration |
| 78 | Ye ZH, 2024 | Efficacy of dexmedetomidine combined with ropivacaine on postoperative analgesia and delirium in elderly patients with total knee arthroplasty | https://trialsearch.who.int/Trial2.aspx?TrialID=IRCT20160430027677N16. 2019. | Excluded | Not i.v. administration |
| 79 | Yu J, 2021 | Effect of different administration methods of dexmedetomidine on recovery of elderly patients undergoing spinal surgery after tracheal intubation under general anesthesia | Front Psychiatry. 2022; 13:816893. | Excluded | Not i.v. administration |
| 80 | Yu S, 2019 | The efficacy and safety of nasal sustained release administration of dextrometadiazine in nasal endoscopic surgery | http://www.who.int/trialsearch/Trial2.aspx?TrialID=ChiCTR2100043968. 2021. | Excluded | Not i.v. administration |
| 81 | Zeng W, 2021 | Dexmedetomidine nasal drip induced sleep in the treatment of anxiety insomnia before operation: a randomized controlled trial | Anesth Analg. 2015; 121(5):1378-1382. | Excluded | Not i.v. administration |
| 82 | Zeng W, 2022 | Intranasal Dexmedetomidine for the Treatment of Pre-operative Anxiety and Insomnia: A Prospective, Randomized, Controlled, and Clinical Trial | http://www.who.int/trialsearch/Trial2.aspx?TrialID=ChiCTR1900025692. 2019. | Excluded | Not i.v. administration |
| 83 | Alexopoulou C, 2013 | Effects of dexmedetomidine on sleep quality in mechanically ventilated critically ill patients | Intensive Care Medicine. 2013; 39:S419-S420. | Excluded | Not a randomized trial |
| 84 | Alexopoulou C, 2014 | Effects of dexmedetomidine on sleep quality in critically ill patients: A pilot study | Anesthesiology. 2014. | Excluded | Not a randomized trial |
| 85 | Cai J, 2020 | Effect of Intraoperative Dexmedetomidine Dose on Postoperative First Night Sleep Quality in Elderly Surgery Patients: A Retrospective Study With Propensity Score-Matched Analysis | Front Med (Lausanne). 2020; 7:528. | Excluded | Not a randomized trial |
| 86 | Duan G, 2020 | The Effects of Intraoperative Dexmedetomidine Use and Its Different Dose on Postoperative Sleep Disturbance in Patients Who Have Undergone Non-Cardiac Major Surgery: A Real-World Cohort Study | Nat Sci Sleep. 2020; 12:209-219. | Excluded | Not a randomized trial |
| 87 | Karren EA, 2016 | Dexmedetomidine for prevention of delirium in elderly patients after non-cardiac surgery | Journal of Thoracic Disease. 2016; 8(12):E1759-E1762. | Excluded | Not a randomized trial |
| 88 | Lu W, 2017 | Effects of dexmedetomidine on sleep quality of patients after surgery without mechanical ventilation in ICU | Medicine (Baltimore). 2017; 96(23):e7081. | Excluded | Not a randomized trial |
| 89 | Su X, 2014 | Prophylactic low-dose dexmedetomidine decreases the incidence of delirium in critically ill elderly patients after noncardiac surgery: a randomized controlled trial | Intensive care medicine. 2014; 40(1):S245‐. | Excluded | Not a randomized trial |
| 90 | Corbett SM, 2005 | Dexmedetomidine does not improve patient satisfaction when compared with propofol during mechanical ventilation | Crit Care Med. 2005; 33(5):940-945 | Excluded | Not saline in control group |
| 91 | Song B, 2019 | The effect of intraoperative use of dexmedetomidine during the daytime operation vs the nighttime operation on postoperative sleep quality and pain under general anesthesia | Nat Sci Sleep. 2019; 11:207-215 | Excluded | Not saline in control group |
| 92 | Shao XC, 2019 | Effect of dexmedetomidine and sufentanil on sleep quality and incidence of nausea and vomiting in patients with analgesia after laparoscopic surgery | World chinese journal of digestology. 2019; 27(20):1290‐1294 | Excluded | Unable to access full article |
| 93 | Xu S, 2022 | Effect of co-administration of intravenous lidocaine and dexmedetomidine on the recovery from laparoscopic hysterectomy: a randomized controlled trial | Minerva Anestesiol. 2023; 89(1-2):10-21 | Excluded | Unable to access full article |
| 94 | Shi CX, 2017 | Intraoperative use of dexmedetomidine promotes postoperative sleep and recovery following radical mastectomy under general anesthesia | Oncotarget. 2017; 8(45):79397-79403 | Excluded | No listed outcome |
| 95 | Aantaa R, 2009 | Sedation in PICU | Acta Anaesth Scand, Supp. 2009; 53(119):3-5. | Excluded | Based on title and abstract |
| 96 | Aantaa R, 2015 | Sedation options for the morbidly obese intensive care unit patient: A concise survey and an agenda for development | Multidisciplinary Respiratory Medicine. 2015; 10(1). | Excluded | Based on title and abstract |
| 97 | Abad GA, 2022 | Dexmedetomidine and postoperative ileus: when sparing opioids is the key | Minerva Anestesiologica. 2022; 88(1-2):2-5. | Excluded | Based on title and abstract |
| 98 | Abd AN, 2011 | Efficacy and safety of dexmedetomidine versus morphine in post-operative cardiac surgery patients | Int J Clin Pharm. 2011; 33(2):150-154. | Excluded | Based on title and abstract |
| 99 | Abdelmageed WM, 2011 | Analgesic properties of a dexmedetomidine infusion after uvulopalatopharyngoplasty in patients with obstructive sleep apnea | Saudi J Anaesth. 2011; 5(2):150-156. | Excluded | Based on title and abstract |
| 100 | Actrn, 2009 | Optimising post-operative sedation for Cardiac Intensive Care in patients undergoing elective cardiac surgery involving cardiopulmonary bypass | http://www.who.int/trialsearch/Trial2.aspx?TrialID=ACTRN12609000199213. 2009. | Excluded | Based on title and abstract |
| 101 | Actrn, 2009 | The use of dexmedetomidine as an analgesic after uvulopalatopharyngoplasty for obstructive sleep apnea | http://www.who.int/trialsearch/Trial2.aspx?TrialID=ACTRN12609000731291. 2009. | Excluded | Based on title and abstract |
| 102 | Actrn, 2013 | Controlled randomized double blind study: comparison of Dexmedetomidine versus Propofol for Maintenance of Anesthesia during invasive procedures In Pediatric Oncology Patients | https://trialsearch.who.int/Trial2.aspx?TrialID=ACTRN12613000149763. 2013. | Excluded | Based on title and abstract |
| 103 | Actrn, 2015 | Sedation Practice in Paediatric Intensive Care Cardiac Patients: cardiac Baby SPICE -pilot randomised trial | http://www.who.int/trialsearch/Trial2.aspx?TrialID=ACTRN12615001304527. 2015. | Excluded | Based on title and abstract |
| 104 | Actrn, 2016 | Postoperative analgesia for elective total knee arthroplasty | http://www.who.int/trialsearch/Trial2.aspx?TrialID=ACTRN12616000912482. 2016. | Excluded | Based on title and abstract |
| 105 | Actrn, 2020 | Improving the ways we put patients to sleep (anaesthesia) for weight loss surgery | https://trialsearch.who.int/Trial2.aspx?TrialID=ACTRN12620000198921. 2020. | Excluded | Based on title and abstract |
| 106 | Adler AC, 2021 | The association of dexmedetomidine on perioperative opioid consumption in children undergoing adenotonsillectomy with and oithout obstructive sleep apnea | Anesth Analg. 2021; 133(5):1260-1268. | Excluded | Based on title and abstract |
| 107 | Agarwal R, 2022 | To assess the efficacy of bupivacaine against levobupivacaine in supraclavicular brachial plexus block | Eur J of Mole Clin Med. 2022; 9(3):5855-5860. | Excluded | Based on title and abstract |
| 108 | Ahmed ASS, 2022 | Comparison between dexmedetomidine and midazolam for pediatric sedation | NeuroQuantology. 2022; 20(6):7762-7774. | Excluded | Based on title and abstract |
| 109 | Ahmed U, 2020 | Anaesthetic management of patients undergoing deep brain simulation: A retrospective review of 8 cases from a tertiary care center of Pakistan | Pak J Med Sci. 2020; 36(7):1737-1741. | Excluded | Based on title and abstract |
| 110 | Akeju O, 2016 | Spatiotemporal dynamics of dexmedetomidine-induced electroencephalogram oscillations | PLoS One. 2016; 11(10):e0163431. | Excluded | Based on title and abstract |
| 111 | Aksu R, 2011 | The comparison of the effects of dexmedetomidine and midazolam sedation on electroencephalography in pediatric patients with febrile convulsion | Paediatric Anaesthesia. 2011; 21(4):373-378. | Excluded | Based on title and abstract |
| 112 | Ala KT, 2022 | Monitoring of nighttime EEG slow-wave activity during dexmedetomidine infusion in patients with hyperactive ICU delirium: An observational pilot study | Acta Anaesth Scand. 2022; 66(10):1211-1218. | Excluded | Based on title and abstract |
| 113 | Alcaraz G, 2020 | Feasibility of awake craniotomy in the pediatric population | Paediatric Anaesthesia. 2020; 30(7):842-843. | Excluded | Based on title and abstract |
| 114 | Aldakhil S, 2020 | The prevalence of emergence delirium and its associated factors among children at a postoperative unit: a retrospective cohort at a middle eastern hospital | Saudi Journal of Anaesthesia. 2020; 14(2):169-176. | Excluded | Based on title and abstract |
| 115 | Algarra NN, 2017 | Does dexmedetomidine offer brain protection? | Minerva Anestesiologica. 2017; 83(12):1224-1226. | Excluded | Based on title and abstract |
| 116 | Alghamdi F, 2020 | opioid-sparing anesthetic technique for pediatric patients undergoing adenoidectomy: a pilot study | J Pain Res. 2020; 13:2997-3004. | Excluded | Based on title and abstract |
| 117 | Almarakbi W, 2014 | Addition of dexmedetomidine to bupivacaine in transversus abdominis plane block potentiates post-operative pain relief among abdominal hysterectomy patients: a prospective randomized controlled trial | Saudi journal of anaesthesia. 2014; 8(2):161‐166. | Excluded | Based on title and abstract |
| 118 | Alnagger N, 2022 | Information processing differences in deep sedation may indicate subjective differences in anaesthetic states | European Journal of Neurology. 2022; 29:805-806. | Excluded | Based on title and abstract |
| 119 | Alvarez A, 2014 | Postoperative analgesia in morbid obesity | Obesity Surgery. 2014; 24(4):652-659. | Excluded | Based on title and abstract |
| 120 | Alyamani OA, 2021 | Perioperative pain management in COVID-19 patients: Considerations and recommendations by the Saudi Anesthesia Society (SAS) and Saudi Society of Pain Medicine (SSPM) | Saudi Journal of Anaesthesia. 2021; 15(1):59-69. | Excluded | Based on title and abstract |
| 121 | Ammar AS, 2012 | Ultrasound-guided single injection infraclavicular brachial plexus block using bupivacaine alone or combined with dexmedetomidine for pain control in upper limb surgery: A prospective randomized controlled trial | Saudi Journal of Anaesthesia. 2012; 6(2):109-114. | Excluded | Based on title and abstract |
| 122 | An B, 2022 | Effects of multimodal analgesia of flurbiprofen axetil, nalbuphine and patient controlled intravenous analgesia on inflammatory factor levels and stress response in patients after laparoscopic radical gynecological malignancy surgery | Pakistan J of Pharm Sci. 2022; 35(2):641-647. | Excluded | Based on title and abstract |
| 123 | Anand VG, 2011 | Effects of dexmedetomidine added to caudal ropivacaine in paediatric lower abdominal surgeries | Indian J Anaesth. 2011; 55(4):340-346. | Excluded | Based on title and abstract |
| 124 | Andersen LPH, 2014 | A systematic review of peri-operative melatonin | Anaesthesia. 2014; 69(10):1163-1171. | Excluded | Based on title and abstract |
| 125 | Andreolio C, 2016 | Prolonged infusion of dexmedetomidine in critically-ill children | Indian Pediatrics. 2016; 53(11):987-989. | Excluded | Based on title and abstract |
| 126 | Ankichetty S, 2011 | A systematic review of the effects of sedatives and anesthetics in patients with obstructive sleep apnea | J Anaesthesiol Clin Pharmacol. 2011; 27(4):447-458. | Excluded | Based on title and abstract |
| 127 | Antonelli M, 2013 | Year in review in Intensive Care Medicine 2012: III. Noninvasive ventilation, monitoring and patient-ventilator interactions, acute respiratory distress syndrome, sedation, paediatrics and miscellanea | Intensive Care Medicine. 2013; 39(4):543-557. | Excluded | Based on title and abstract |
| 128 | Ard J, 2003 | Awake craniotomy with dexmedetomidine in pediatric patients | J Neurosurg Anesthesiol. 2003; 15(3):263-266. | Excluded | Based on title and abstract |
| 129 | Arlachov Y, 2012 | Sedation/anaesthesia in paediatric radiology | British J Radi. 2012; 85(1019):e1018-e1031. | Excluded | Based on title and abstract |
| 130 | Arthurs OJ, 2013 | Anaesthesia or sedation for paediatric MRI: Advantages and disadvantages | Current Opinion in Anaesthesiology. 2013; 26(4):489-494. | Excluded | Based on title and abstract |
| 131 | Atkins JH, 2010 | Anesthetic considerations and surgical caveats for awake airway surgery | Anesthesiology Clinics. 2010; 28(3):555-575. | Excluded | Based on title and abstract |
| 132 | Atkins JH, 2016 | Sleep-disordered breathing and the patient undergoing endoscopy: Considerations for optimization of periprocedural care | Techniques in Gastrointestinal Endoscopy. 2016; 18(1):7-12. | Excluded | Based on title and abstract |
| 133 | Atkins JH, 2018 | Drug-induced sleep endoscopy: from obscure technique to diagnostic tool for assessment of obstructive sleep apnea for surgical interventions | Curr Opin Anaesthesiol. 2018; 31(1):120-126. | Excluded | Based on title and abstract |
| 134 | Atri A, 2022 | Efficacy of dexmedetomidine as an adjuvant to levobupivacaine in caudal anaesthesia in paediatric patients | International journal of pharmaceutical and clinical research. 2022; 14(7):254‐261. | Excluded | Based on title and abstract |
| 135 | Aubanel S, 2020 | Therapeutic options for agitation in the intensive care unit | Anaesthesia Critical Care and Pain Medicine. 2020; 39(5):639-646. | Excluded | Based on title and abstract |
| 136 | Auckley D, 2007 | Perioperative considerations in patients with obstructive sleep apnea | Current Respiratory Medicine Reviews. 2007; 3(4):245-253. | Excluded | Based on title and abstract |
| 137 | Auckley D, 2012 | Preoperative screening and perioperative care of the patient with sleep-disordered breathing | Current Opinion in Pulmonary Medicine. 2012; 18(6):588-595. | Excluded | Based on title and abstract |
| 138 | Awad M, 2019 | Drug-induced sleep endoscopy | Atlas Oral Maxillofac Surg Clin North Am. 2019; 27(1):7-10. | Excluded | Based on title and abstract |
| 139 | Azizkhani R, 2021 | Comparison of the effects of dexmedetomidine and propofol in reducing recovery agitation in pediatric patients after ketamine procedural sedation in emergency department | Journal of Research in Medical Sciences. 2021; 26(1). | Excluded | Based on title and abstract |
| 140 | Baier NM,2016 | Intranasal dexmedetomidine: An effective sedative agent for electroencephalogram and auditory brain response testing | Paediatric Anaesthesia. 2016; 26(3):280-285. | Excluded | Based on title and abstract |
| 141 | Baker R, 2014 | Altered activity in the central medial thalamus precedes changes in the neocortex during transitions into both sleep and propofol anesthesia | Journal of Neuroscience. 2014; 34(40):13326-13335. | Excluded | Based on title and abstract |
| 142 | Balavenkataraman A, 2022 | Dexmedetomidine as a otential cause of central sleep apnea | American Journal of Respiratory and Critical Care Medicine. 2022; 205(1). | Excluded | Based on title and abstract |
| 143 | Bamgbade OA, 2009 | Dexmedetomidine anaesthesia for patients with obstructive sleep apnoea undergoing bariatric surgery | Eur J Anaesthesiol. 2009; 26(2):176-177. | Excluded | Based on title and abstract |
| 144 | Banh HL, 2012 | Management of delirium in adult critically ill patients: An overview | Journal of Pharmacy and Pharmaceutical Sciences. 2012; 15(4):499-509. | Excluded | Based on title and abstract |
| 145 | Banik S, 2019 | Awake craniotomy in a super obese patient using high flow nasal cannula oxygen therapy (HFNC) | Anaesthesist. 2019; 68(11):780-783. | Excluded | Based on title and abstract |
| 146 | Banks MI, 2017 | Correlational studies of unconsciousness under anaesthesia: How far can preclinical studies take us? | British Journal of Anaesthesia. 2017; 119(6):1079-1081. | Excluded | Based on title and abstract |
| 147 | Barnes SS, 2016 | Propofol infusion in children | Anaesthesia. 2016; 71(8):986-987. | Excluded | Based on title and abstract |
| 148 | Barnes SS, 2016 | Sedative choice and ventilator-associated patient outcomes: Don't sleep on delirium | Ann Transl Med. 2016; 4(2):34. | Excluded | Based on title and abstract |
| 149 | Barr J, 2013 | Clinical practice guidelines for the management of pain, agitation, and delirium in adult patients in the intensive care unit | Critical Care Medicine. 2013; 41(1):263-306. | Excluded | Based on title and abstract |
| 150 | Barr J, 2013 | The pain, agitation, and delirium care bundle: Synergistic benefits of implementing the 2013 pain, agitation, and delirium guidelines in an integrated and interdisciplinary fashion | Critical Care Medicine. 2013; 41(9 SUPPL.1):S99-S115. | Excluded | Based on title and abstract |
| 151 | Barra ME, 2019 | Multidisciplinary approach to sedation and early rehabilitation of intubated patients admitted to the neuroscience intensive care unit | Neurocritical Care. 2019; 31(1):S142. | Excluded | Based on title and abstract |
| 152 | Barry LA, 2006 | Intravenous regional anesthesia (Bier block) | Techniques in Regional Anesthesia and Pain Management. 2006; 10(3):123-131. | Excluded | Based on title and abstract |
| 153 | Bartholomew KJ, 2021 | Ultrasound-guided erector spinae plane block as part of a multimodal analgesic approach in a dog with acute pancreatitis | Veterinary Anaesthesia and Analgesia. 2021; 48(4):629-632. | Excluded | Based on title and abstract |
| 154 | Bartoletta KM, 2022 | Novel use of intranasal dexmedetomidine for refractory irritability in pediatric home care | J palliat med. 2023; 26(4):596-598 | Excluded | Based on title and abstract |
| 155 | Bates AJ, 2017 | Combining computational fluid dynamics and 3D-cine mri to determine the relationship between upper airway motion and breathing effort in pediatric obstructive sleep apnea | American Journal of Respiratory and Critical Care Medicine. 2017; 195. | Excluded | Based on title and abstract |
| 156 | Batistaki C, 2017 | Special precaution for the elderly patient | CardioVascular and Interventional Radiology. 2017; 40(2):S56-S57. | Excluded | Based on title and abstract |
| 157 | Baughman VL,2005 | Dexmedetomidine in awake craniotomy: A technical note - Commentary | Surgical Neurology. 2005; 63(2):116-117. | Excluded | Based on title and abstract |
| 158 | Baumgartner L, 2018 | Melatonin for the prevention of intensive care unit (ICU) delirium | Critical Care Medicine. 2018; 46:454. | Excluded | Based on title and abstract |
| 159 | Baumgartner L, 2019 | Effectiveness of melatonin for the prevention of intensive care unit delirium | Pharmacotherapy. 2019; 39(3):280-287. | Excluded | Based on title and abstract |
| 160 | Bawazeer M, 2020 | Adjunct low-dose ketamine infusion vs standard of care in mechanically ventilated critically ill patients at a Tertiary Saudi Hospital (ATTAINMENT Trial): Study protocol for a randomized, prospective, pilot, feasibility trial | Trials. 2020; 21(1):288. | Excluded | Based on title and abstract |
| 161 | Bayat A, 2010 | Analgesia and sedation for children undergoing burn wound care | Expert Review of Neurotherapeutics. 2010; 10(11):1747-1759. | Excluded | Based on title and abstract |
| 162 | Belleville JP, 1992 | Effects of intravenous dexmedetomidine in humans: I. Sedation, ventilation, and metabolic rate | Anesthesiology. 1992; 77(6):1125-1133. | Excluded | Based on title and abstract |
| 163 | Belur NS, 2020 | Predicting deep hypnotic state from sleep brain rhythms using deep learning: A data-repurposing approach | Anesth Analg. 2020; 130(5):1211-1221. | Excluded | Based on title and abstract |
| 164 | Bennitz J, 2018 | Effect of dexmedetomidine on intracranial eeg recordings from the human hippocampus and neocortex | Canadian Journal of Anesthesia. 2018; 65(1):S72-S73. | Excluded | Based on title and abstract |
| 165 | Bergendahl H, 2006 | Clonidine in paediatric anaesthesia: Review of the literature and comparison with benzodiazepines for premedication | Acta Anaesthesiologica Scandinavica. 2006; 50(2):135-143. | Excluded | Based on title and abstract |
| 166 | Bergese SD, 2019 | ATHENA: A phase 3, open-label study of the safety and effectiveness of oliceridine (TRV130), a g-protein selective agonist at the μ-opioid receptor, in patients with moderate to severe acute pain requiring parenteral opioid therapy | Journal of Pain Research. 2019; 12:3113-3126. | Excluded | Based on title and abstract |
| 167 | Berkenbosch JW, 2014 | Long-term dexmedetomidine use for pediatric critical care sedation: No need to sleep on it | Pediatric Critical Care Medicine. 2014; 15(8):771-773. | Excluded | Based on title and abstract |
| 168 | Bhalotra AR, 2019 | Opioid-free anaesthesia in children with severe mandibular hypoplasia and TMJ ankylosis with sleep apnoea for mandibular distraction osteogenesis | Indian Journal of Anaesthesia. 2019; 63(5):412-414. | Excluded | Based on title and abstract |
| 169 | Bhana N, 2000 | Sedative/analgesic-dexmedetomidine | Manufacturing Chemist. 2000; 71(10):39. | Excluded | Based on title and abstract |
| 170 | Bhavani S, 2016 | Non-operating room anesthesia in the endoscopy unit | Gastrointestinal Endoscopy Clinics of North America. 2016; 26(3):471-483. | Excluded | Based on title and abstract |
| 171 | Bily B, 2017 | Dexmedetomidine and its use in anaesthesia and intensive care | Anesteziologie a Intenzivni Medicina. 2017; 28(2):114-126. | Excluded | Based on title and abstract |
| 172 | Bohman JK, 2013 | Awake tracheal intubation in an 8-year-old girl with McCune-Albright syndrome | A and A Case Reports. 2013; 1(1):23-25. | Excluded | Based on title and abstract |
| 173 | Bohringer C, 2018 | Is it time for an expanded role of dexmedetomidine in contemporary anesthesia practice? - A clinician's perspective | Transl Perioper Pain Med. 2018; 5(3):55-62. | Excluded | Based on title and abstract |
| 174 | Bojaxhi E, 2021 | Reduced pain and opioid use in the early postoperative period in patients undergoing a frontotemporal craniotomy under regional vs general anesthesia | World Neurosurgery. 2021; 150:e31-e37. | Excluded | Based on title and abstract |
| 175 | Bol C, 1997 | Pharmacokinetic-pharmacodynamic characterization of the cardiovascular, hypnotic, EEG and ventilatory responses to dexmedetomidine in the rat | Journal of Pharmacology and Experimental Therapeutics. 1997; 283(3):1051-1058. | Excluded | Based on title and abstract |
| 176 | Bong CL, 2019 | Randomised controlled trial of dexmedetomidine sedation vs general anaesthesia for inguinal hernia surgery on perioperative outcomes in infants | British Journal of Anaesthesia. 2019; 122(5):662-670. | Excluded | Based on title and abstract |
| 177 | Bonhomme V, 2019 | General anesthesia: A probe to explore consciousness | Front Syst Neurosci. 2019; 13:36. | Excluded | Based on title and abstract |
| 178 | Bosch OG, 2022 | Dexmedetomidine in psychiatry: repurposing of its fast-acting anxiolytic, analgesic and sleep modulating properties | Pharmacopsychiatry. 2023; 56(2):44-50 | Excluded | Based on title and abstract |
| 179 | Boswinkel JP, 2005 | The pharmacology of sedation | Pediatric Annals. 2005; 34(8):607-613. | Excluded | Based on title and abstract |
| 180 | Bourne RS, 2008 | Drug treatment of delirium: Past, present and future | Journal of Psychosomatic Research. 2008; 65(3):273-282. | Excluded | Based on title and abstract |
| 181 | Bowton DL, 2004 | Delirium - The cost of inattention | Critical Care Medicine. 2004; 32(4):1080-1081. | Excluded | Based on title and abstract |
| 182 | Boyce-Rustay JM, 2008 | Desipramine potentiation of the acute depressant effects of ethanol: Modulation by α2-adrenoreceptors and stress | Neuropharmacology. 2008; 55(5):803-811. | Excluded | Based on title and abstract |
| 183 | Brier LM, 2019 | Separability of calcium slow waves and functional connectivity during wake, sleep, and anesthesia | Journal of Cerebral Blood Flow and Metabolism. 2019; 39(1):474-475. | Excluded | Based on title and abstract |
| 184 | Broese M, 2012 | α-Adrenergic receptor function, arousal and sleep: Mechanisms and therapeutic implications | Pharmacopsychiatry. 2012; 45(6):209-216. | Excluded | Based on title and abstract |
| 185 | Brooks PM, 2022 | Anesthetic management and outcomes of patients with epidermolysis bullosa: experience at a tertiary referral center | Anesthesia and Analgesia. 2022; 134(4):810-821. | Excluded | Based on title and abstract |
| 186 | Brown EN, 2011 | General anesthesia and altered states of arousal: A systems neuroscience analysis | Annu Rev Neurosci. 2011; 34:601-28 | Excluded | Based on title and abstract |
| 187 | Brown Z, 2012 | Anaesthesia for elective ear, nose and throat surgery in children | Anaesthesia and Intensive Care Medicine. 2012; 13(5):234-239. | Excluded | Based on title and abstract |
| 188 | Brown ZE, 2021 | Update on ENT anaesthesia in children | Anaesthesia and Intensive Care Medicine. 2021; 22(11):723-728. | Excluded | Based on title and abstract |
| 189 | Brummel NE, 2013 | preventing delirium in the intensive care unit | Critical Care Clinics. 2013; 29(1):51-65. | Excluded | Based on title and abstract |
| 190 | Brummett CM, 2006 | The use of alpha-2 agonists in peripheral nerve blocks: a review of the history of clonidine and a look at a possible future for dexmedetomidine | Seminars in Anesthesia, Perioperative Medicine and Pain. 2006; 25(2):84-92. | Excluded | Based on title and abstract |
| 191 | Bugada D, 2021 | Opioid free anesthesia: Evidence for short and long-term outcome | Minerva Anestesiologica. 2021; 87(2):230-237. | Excluded | Based on title and abstract |
| 192 | Builes A, 2017 | Effect of intravenous dexmedetomidine in the duration of infraclavicular block: randomized, double-blind study | Canadian journal of anesthesia. 2017; 64(1):S161‐S163. | Excluded | Based on title and abstract |
| 193 | Bulow NMH, 2007 | Opioid consumption in total intravenous anesthesia is reduced with dexmedetomidine: a comparative study with remifentanil in gynecologic videolaparoscopic surgery | Journal of Clinical Anesthesia. 2007; 19(4):280-285. | Excluded | Based on title and abstract |
| 194 | Burns J, 2017 | The use of dexmedetomidine in pediatric palliative care: a preliminary study | Journal of Palliative Medicine. 2017; 20(7):779-783. | Excluded | Based on title and abstract |
| 195 | Burry L, 2019 | Pharmacological interventions for the treatment of delirium in critically ill adults | Cochrane Database of Systematic Reviews. 2019(9). | Excluded | Based on title and abstract |
| 196 | Bush B, 2018 | Postoperative bradycardia following adenotonsillectomy in children: Does intraoperative administration of dexmedetomidine play a role? | Int J Pediatr Otorhinolaryngol. 2018; 104:210-215. | Excluded | Based on title and abstract |
| 197 | Buvanendran A, 2007 | Useful adjuvants for postoperative pain management | Best Practice and Research in Clinical Anaesthesiology. 2007; 21(1):31-49. | Excluded | Based on title and abstract |
| 198 | Buvanendran A, 2009 | Multimodal analgesia for controlling acute postoperative pain | Current Opinion in Anaesthesiology. 2009; 22(5):588-593. | Excluded | Based on title and abstract |
| 199 | Caetta A, 2021 | Postoperative respiratory complications after adenotonsillectomy in children with obstructive sleep apnea | Int J Pediatr Otorhinolaryngol. 2021; 148:110835. | Excluded | Based on title and abstract |
| 200 | Cammaroto G, 2020 | In reference to dexmedetomidine versus propofol at different sedation depths during drug-induced sleep endoscopy: A randomized trial | Laryngoscope. 2020; 130(5):E381. | Excluded | Based on title and abstract |
| 201 | Cao Q, 2017 | Comparison of sedation by intranasal dexmedetomidine and oral chloral hydrate for pediatric ophthalmic examination | Paediatric Anaesthesia. 2017; 27(6):629-636. | Excluded | Based on title and abstract |
| 202 | Capasso R,2016 | Variable findings for drug-induced sleep endoscopy in obstructive sleep apnea with propofol versus dexmedetomidine | Otolaryngol Head Neck Surg. 2016; 154(4):765-770. | Excluded | Based on title and abstract |
| 203 | Carlton EF, 2017 | Management of multifactorial infant delirium with intravenous haloperidol in the setting of over sedation and poor enteral absorption | Journal of Child and Adolescent Psychopharmacology. 2017; 27(3):289-290. | Excluded | Based on title and abstract |
| 204 | Carney L, 2013 | Safety and effectiveness of dexmedetomidine in the pediatric intensive care unit (SAD-PICU) | Canadian Journal of Hospital Pharmacy. 2013; 66(1):21-27. | Excluded | Based on title and abstract |
| 205 | Carron M, 2020 | Perioperative care of the obese patient | British Journal of Surgery. 2020; 107(2):e39-e55. | Excluded | Based on title and abstract |
| 206 | Casey CP, 2022 | Distinct EEG signatures differentiate unconsciousness and disconnection during anaesthesia and sleep | Br J Anaesth. 2022; 128(6):1006-1018. | Excluded | Based on title and abstract |
| 207 | Casey P, 2021 | Improving pre-operative pharmacological anxiety management in paediatric patients undergoing surgical procedures in a district general hospital | Anaesthesia. 2021; 76(SUPPL 6):26. | Excluded | Based on title and abstract |
| 208 | Cataldo SH, 2019 | Using the superNO2VA device on a patient with a known difficult airway: A case report facilitating fiberoptic intubation and postoperative nasal positive pressure | A and A Practice. 2019; 12(5):160-164. | Excluded | Based on title and abstract |
| 209 | Chamadia S, 2020 | A pharmacokinetic and pharmacodynamic study of oral dexmedetomidine | Anesthesiology. 2020; 133(6):1223-1233. | Excluded | Based on title and abstract |
| 210 | Chan A, 2010 | Alpha-2 agonists in acute pain management | Expert Opinion on Pharmacotherapy. 2010; 11(17):2849-2868. | Excluded | Based on title and abstract |
| 211 | Chan BS, 2022 | A case of flubromazolam overdose | Clinical Toxicology. 2022; 60(SUPPL 1):13. | Excluded | Based on title and abstract |
| 212 | Chandrakantan A, 2011 | Multimodal therapies for postoperative nausea and vomiting, and pain | British Journal of Anaesthesia. 2011; 107(SUPPL. 1):i27-i40. | Excluded | Based on title and abstract |
| 213 | Chandrakantan A, 2020 | Pediatric obstructive sleep apnea revisited: Perioperative considerations for the pediatric Anesthesiologist | Int J Pediatr Otorhinolaryngol. 2020; 139:110420. | Excluded | Based on title and abstract |
| 214 | Chang ET, 2017 | Dexmedetomidine versus propofol during drug-induced sleep endoscopy and sedation: a systematic review | Sleep Breath. 2017; 21(3):727-735. | Excluded | Based on title and abstract |
| 215 | Chang GY, 2017 | Peri-operative outcomes of congenital central hypoventilation syndrome patients undergoing general anesthesia | American Journal of Respiratory and Critical Care Medicine. 2017; 195. | Excluded | Based on title and abstract |
| 216 | Chang YR, 2019 | Are management decisions in critical patients changed with use of hemodynamic parameters from transpulmonary thermodilution technique? | Annals of Translational Medicine. 2019; 7(16). | Excluded | Based on title and abstract |
| 217 | Charakorn N, 2016 | Drug-Induced Sleep Endoscopy | Otolaryngologic Clinics of North America. 2016; 49(6):1359-1372. | Excluded | Based on title and abstract |
| 218 | Chassery C, 2021 | Total knee arthroplasty under quadruple nerve block with ropivacaine 0.32%: Effect of addition of intravenous dexmedetomidine to intravenous dexamethasone on analgesic duration | Regional Anesthesia and Pain Medicine. 2021; 46(2):104-110. | Excluded | Based on title and abstract |
| 219 | Chatterjee D, 2014 | Anesthetic dilemmas for dynamic evaluation of the pediatric upper airway | Semin Cardiothorac Vasc Anesth. 2014; 18(4):371-378. | Excluded | Based on title and abstract |
| 220 | Chaudhary M, 2021 | Effect of two different doses of dexmedetomidine on extubation quality score during tracheal extubation in adult patients-a randomised controlled trial | Journal of clinical and diagnostic research. 2021; 15(12):UC10‐UC14. | Excluded | Based on title and abstract |
| 221 | Chawla S, 2010 | Peri-operative use of dexmedetomidine in airway reconstruction surgery for obstructive sleep apnoea | J Laryngol Otol. 2010; 124(1):67-72. | Excluded | Based on title and abstract |
| 222 | Chen JL, 2022 | Bariatric surgery in patients with obstructive sleep apnea | International Anesthesiology Clinics. 2022; 60(2):50-58. | Excluded | Based on title and abstract |
| 223 | Chen K, 2022 | Acute severe withdrawal associated with 4-fluorophenibut and bromazolam exposure | Clinical Toxicology. 2022; 60:14. | Excluded | Based on title and abstract |
| 224 | Chen L, 2021 | Comparative effects of dexmedetomidine and midazolam on dreaming of patients undergoing flexible bronchoscopy during general anesthesia | BMC Anesthesiol. 2023; 23(1):34. | Excluded | Based on title and abstract |
| 225 | Chen L, 2023 | Application of opioid-free general anesthesia for gynecological laparoscopic surgery under ERAS protocol: a non-inferiority randomized controlled trial | Med Sci Monit. 2021; 27:e929000. | Excluded | Based on title and abstract |
| 226 | Chen P, 2020 | Efficacy and safety of dexmedetomidine combined with tramadol for patient-controlled intravenous analgesia in Chinese surgical patients: A systematic review and meta-analysis | Medicine (United States). 2020; 99(3). | Excluded | Based on title and abstract |
| 227 | Chen YT, 2021 | The use of propofol versus dexmedetomidine for patients receiving drug-induced sleep endoscopy: A meta-analysis of randomized controlled trials | Journal of Clinical Medicine. 2021; 10(8). | Excluded | Based on title and abstract |
| 228 | Cheng X, 2014 | Comparison of the effects of dexmedetomidine-ketamine and sevoflurane-sufentanil anesthesia in children with obstructive sleep apnea after uvulopalatopharyngoplasty: An observational study | J Anaesthesiol Clin Pharmacol. 2014; 30(1):31-35. | Excluded | Based on title and abstract |
| 229 | Cheng XQ, 2021 | Anti-nociceptive effects of dexmedetomidine infusion plus modified intercostal nerve block during single-port thoracoscopic lobectomy: A double-blind, randomized controlled trial | Pain Physician. 2021; 24(5):E565-E572. | Excluded | Based on title and abstract |
| 230 | Chepyala P, 2007 | Treatment of cyclic vomiting syndrome | Current Treatment Options in Gastroenterology. 2007; 10(4):273-282. | Excluded | Based on title and abstract |
| 231 | Cherng CH, 2007 | Ketamine-induced emergence reactions after desflurane anaesthesia | European Journal of Anaesthesiology. 2007; 24(2):200-201. | Excluded | Based on title and abstract |
| 232 | Cheung CW, 2011 | Analgesic and sedative effects of intranasal dexmedetomidine in third molar surgery under local anaesthesia | British journal of anaesthesia. 2011; 107(3):430‐437. | Excluded | Based on title and abstract |
| 233 | Cheung H, 2021 | Feasibility of non-opioid analgesic in breast-conserving surgery under general anesthesia with laryngeal mask airway | Journal of shanghai jiaotong university (medical science). 2021; 41(5):637‐641. | Excluded | Based on title and abstract |
| 234 | ChiCtr, 2015 | Compare recovery period with different doses of anesthetic drugs in cataract surgery in infant | https://trialsearch.who.int/Trial2.aspx?TrialID=ChiCTR-IPR-15006112. 2015. | Excluded | Based on title and abstract |
| 235 | ChiCtr, 2015 | Intraoperative appliance of dexmedetomidine during laparoscopic resection of colorectal cancer: influence on early postoperative intestinal function | https://trialsearch.who.int/Trial2.aspx?TrialID=ChiCTR-IOR-15006518. 2015. | Excluded | Based on title and abstract |
| 236 | ChiCtr, 2015 | The analgesic effect of ropivacaine combined with dexmedetomidine for incision infiltration after laparoscropic cholecystectomy | https://trialsearch.who.int/Trial2.aspx?TrialID=ChiCTR-IOR-15006659. 2015. | Excluded | Based on title and abstract |
| 237 | ChiCtr, 2016 | Local anesthetic infiltration with different concentration of dexmedetomidine cocktail and continuous femoral nerve block in postoperative analgesia of total knee arthroplasy: a prospective randomized controlled study | https://trialsearch.who.int/Trial2.aspx?TrialID=ChiCTR-INR-16009825. 2016. | Excluded | Based on title and abstract |
| 238 | ChiCtr, 2018 | Application of dexmedetomidine in complication surgery and accelerated rehabilitation of gastrointestinal surgery | https://trialsearch.who.int/Trial2.aspx?TrialID=ChiCTR1800019376. 2018. | Excluded | Based on title and abstract |
| 239 | ChiCtr, 2018 | The effects of intraoperative dexmedetomidine during monitored anesthesian care on patients' postoperative recovery and respiratory function | https://trialsearch.who.int/Trial2.aspx?TrialID=ChiCTR1800019642. 2018. | Excluded | Based on title and abstract |
| 240 | ChiCtr, 2020 | A comparison of safety and efficacy of dexmedetomidine-propofol versus dexmedetomidine alone or sedation during drug-induced sleep endoscopy: a prospective, randomized study | https://trialsearch.who.int/Trial2.aspx?TrialID=ChiCTR2000034520. 2020. | Excluded | Based on title and abstract |
| 241 | ChiCtr, 2020 | Effect of remifentanil combined with dexmedetomidine on postoperative sleep quality in patients undergoing thoracoscopic lobectomy | http://www.who.int/trialsearch/Trial2.aspx?TrialID=ChiCTR2000034724. 2020. | Excluded | Based on title and abstract |
| 242 | ChiCtr, 2020 | Effect of ultrasound-guided nerve block on early rehabilitation of patients undergoing total knee arthroplasty | http://www.who.int/trialsearch/Trial2.aspx?TrialID=ChiCTR2000031258. 2020. | Excluded | Based on title and abstract |
| 243 | ChiCtr, 2020 | The effect of dexmedetomidine for anesthesia maintenance and postoperative analgesia on the non-cardiac surgery prognosis of high-risk elderly patients: a prospective, randomized, double-blind, controlled trial | https://trialsearch.who.int/Trial2.aspx?TrialID=ChiCTR2000030566. 2020. | Excluded | Based on title and abstract |
| 244 | ChiCtr, 2021 | Application of opioid-free anesthesia under ERAS in gynecological laparoscopic surgery | https://trialsearch.who.int/Trial2.aspx?TrialID=ChiCTR2100052761. 2021. | Excluded | Based on title and abstract |
| 245 | ChiCtr, 2021 | Effect of stellate ganglion block on peri-operative cerebral function in laparoscopic abdominal surgery | https://trialsearch.who.int/Trial2.aspx?TrialID=ChiCTR2100048044. 2021. | Excluded | Based on title and abstract |
| 246 | ChiCtr, 2021 | The impact of dexmedetomidine on postoperative delirium in patients with obstructive sleep apnea after non-cardiac surgery | http://www.who.int/trialsearch/Trial2.aspx?TrialID=ChiCTR2100043849. 2021. | Excluded | Based on title and abstract |
| 247 | Chidambaran V, 2018 | Anesthetic and pharmacologic considerations in perioperative care of obese children | Journal of Clinical Anesthesia. 2018; 45:39-50. | Excluded | Based on title and abstract |
| 248 | Chikkahanumanthappa NB, 2016 | A comparative study of sedation with dexmedetomidine or midazolam during spinal anesthesia | Anaesthesia, Pain and Intensive Care. 2016; 20(3):328-333. | Excluded | Based on title and abstract |
| 249 | Chirinos JD, 2023 | Patient hesitancy in perioperative clinical trial enrollment during the COVID-19 pandemic | PLoS One. 2023; 18(1):e0279643. | Excluded | Based on title and abstract |
| 250 | Cho G, 2015 | Maxillomandibular advancement-the name tells half the story | Sleep Medicine. 2015; 16:S17-S18. | Excluded | Based on title and abstract |
| 251 | Cho JS, 2015 | Comparison of three sedation regimens for drug-induced sleep endoscopy | Sleep Breath. 2015; 19(2):711-717. | Excluded | Based on title and abstract |
| 252 | Cho YJ, 2019 | Pharmacologic interventions for postoperative nausea and vomiting after thyroidectomy: A protocol for systematic review and network meta-analysis | Medicine (United States). 2019; 98(7). | Excluded | Based on title and abstract |
| 253 | Chrysostomou C, 2009 | Dexmedetomidine use in a pediatric cardiac intensive care unit: can we use it in infants after cardiac surgery? | Pediatr Crit Care Med. 2009; 10(6):654-660. | Excluded | Based on title and abstract |
| 254 | Chung F, 2009 | Screening for obstructive sleep apnea before surgery: Why is it important? | Current Opinion in Anaesthesiology. 2009; 22(3):405-411. | Excluded | Based on title and abstract |
| 255 | Chung SA, 2008 | A systemic review of obstructive sleep apnea and its implications for anesthesiologists | Anesthesia and Analgesia. 2008; 107(5):1543-1563. | Excluded | Based on title and abstract |
| 256 | Citrome LL, 2022 | Dexmedetomidine orally dissolving film for acute agitation associated with schizophrenia or bipolar disorder: SERENITY I and SERENITY II trials | CNS Spectrums. 2022; 27(2):242. | Excluded | Based on title and abstract |
| 257 | Coetzee JF, 2010 | Safety of pain control with morphine: New (and old) aspects of morphine pharmacokinetics and pharmacodynamics | Southern African Journal of Anaesthesia and Analgesia. 2010; 16(2):7-15. | Excluded | Based on title and abstract |
| 258 | Collins CE, 2010 | Challenges in pediatric ambulatory anesthesia: kids are different | Anesthesiology Clinics. 2010; 28(2):315-328. | Excluded | Based on title and abstract |
| 259 | Colson JD, 2005 | The pharmacology of sedation | Pain Physician. 2005; 8(3):297-308. | Excluded | Based on title and abstract |
| 260 | Congiusta AD, 2021 | The effects of dexmedetomidine on anesthetic outcomes following maxillomandibular advancement surgery | Journal of Oral and Maxillofacial Surgery. 2021; 79(10):e17-e18. | Excluded | Based on title and abstract |
| 261 | Cooke SE, 2002 | Clinical practice guidelines for the sustained use of sedatives and analgesics in the critically ill adult | American Journal of Health-System Pharmacy. 2002; 59(2):150-178. | Excluded | Based on title and abstract |
| 262 | Corbett SM, 2008 | Medication-related complications in the trauma patient | Journal of Intensive Care Medicine. 2008; 23(2):91-108. | Excluded | Based on title and abstract |
| 263 | Cortellazzo WL, 2022 | Recovery characteristics and parental satisfaction in pediatric procedural sedation | Paediatric Anaesthesia. 2022; 32(3):452-461. | Excluded | Based on title and abstract |
| 264 | Cortes BA, 2021 | Diagnosis, prevention, and management of delirium in the intensive cardiac care unit | Am Heart J. 2021; 232:164-176. | Excluded | Based on title and abstract |
| 265 | Costa D, 2021 | Anaesthesia for deep brain stimulation surgery-Casuistics from Egas Moniz Hospital, Lisbon, Portugal | Anesthesia and Analgesia. 2021; 133(3 SUPPL 2):975. | Excluded | Based on title and abstract |
| 266 | Costi D, 2014 | Effects of sevoflurane versus other general anaesthesia on emergence agitation in children | Cochrane Database of Systematic Reviews. 2014(9). | Excluded | Based on title and abstract |
| 267 | Cousineau J, 2021 | Management of obstructive sleep apnea in children: a Canada-wide survey | Journal of Otolaryngology - Head and Neck Surgery. 2021; 50(1). | Excluded | Based on title and abstract |
| 268 | Cozzi G, 2016 | Intranasal dexmedetomidine for procedural sedation | Medico e Bambino. 2016; 35(10):641-645. | Excluded | Based on title and abstract |
| 269 | Crassous PA, 2007 | Interest of α2-adrenergic agonists and antagonists in clinical practice: Background, facts and perspectives | Current Topics in Medicinal Chemistry. 2007; 7(2):187-194. | Excluded | Based on title and abstract |
| 270 | Crean P, 2004 | Sedation and neuromuscular blockade in paediatric intensive care; practice in the United Kingdom and North America | Paediatric Anaesthesia. 2004; 14(6):439-442. | Excluded | Based on title and abstract |
| 271 | Cruickshank M, 2016 | Alpha-2 agonists for sedation of mechanically ventilated adults in intensive care units: A systematic review | Health Technology Assessment. 2016; 20(25):1-118. | Excluded | Based on title and abstract |
| 272 | Ctri, 2012 | Does Dexmedetomidine[a sleep inducing drug] prevent injection pain due to Propofol [an anaesthetic drug]?- a study | https://trialsearch.who.int/Trial2.aspx?TrialID=CTRI/2012/01/002364. 2012. | Excluded | Based on title and abstract |
| 273 | Ctri, 2015 | Comparison of combinations of Dexmedetomidine-Ketamine with propofol-ketamine to put the children for Ct scan to sleep | https://trialsearch.who.int/Trial2.aspx?TrialID=CTRI/2015/04/005717. 2015. | Excluded | Based on title and abstract |
| 274 | Ctri, 2017 | Comparison of only ketamine nebulization and combined ketamine dexmedetomidine nebulization before putting paediatric patients under anaesthesia for surgeries as to produce sleep and to relieve anxiety | https://trialsearch.who.int/Trial2.aspx?TrialID=CTRI/2017/03/008188. 2017. | Excluded | Based on title and abstract |
| 275 | Ctri, 2017 | Comparison of two doses of drug dexmedetomidine via inhalation through oral mask route for sleep and pain in children | https://trialsearch.who.int/Trial2.aspx?TrialID=CTRI/2017/10/010276. 2017. | Excluded | Based on title and abstract |
| 276 | Ctri, 2017 | Comparison of two mild sleep medicines (dexmedetomidine given under the tongue and midazolam given as an oral syrup) to calm children just before surgery | https://trialsearch.who.int/Trial2.aspx?TrialID=CTRI/2017/12/010925. 2017. | Excluded | Based on title and abstract |
| 277 | Ctri, 2017 | How does a sleeping medicine influence the blood particles during knee surgery | https://trialsearch.who.int/Trial2.aspx?TrialID=CTRI/2017/08/009548. 2017. | Excluded | Based on title and abstract |
| 278 | Ctri, 2017 | Recovery of patients after sleep dose of propofol vs dexmedetomidine | https://trialsearch.who.int/Trial2.aspx?TrialID=CTRI/2017/09/009795. 2017. | Excluded | Based on title and abstract |
| 279 | Ctri, 2019 | To see the effect of 3 different doses of same drug called dexmedetomidine used to put the children to sleep before operation | https://trialsearch.who.int/Trial2.aspx?TrialID=CTRI/2019/05/019295. 2019. | Excluded | Based on title and abstract |
| 280 | Ctri, 2019 | Which is better gaseous drugs or liquid drugs given through blood vessel used for anaesthesia (sleep) for ease of getting less blood on operation site ? | https://trialsearch.who.int/Trial2.aspx?TrialID=CTRI/2019/06/019625. 2019. | Excluded | Based on title and abstract |
| 281 | Ctri, 2020 | Comparison of three different drug regimens for sedation in children undergoing MRI | https://trialsearch.who.int/Trial2.aspx?TrialID=CTRI/2020/01/022949. 2020. | Excluded | Based on title and abstract |
| 282 | Ctri, 2020 | Effects of addition of low dose of one medicine (midazolam) before operation on other medicines used for sedation (propofol-dexmedetomidine combination) for endoscopic sleep study in obstructive sleep apnea patients | https://trialsearch.who.int/Trial2.aspx?TrialID=CTRI/2020/12/030128. 2020. | Excluded | Based on title and abstract |
| 283 | Ctri, 2020 | Study on a new technique for pain relief after spine surgery | https://trialsearch.who.int/Trial2.aspx?TrialID=CTRI/2020/07/026430. 2020. | Excluded | Based on title and abstract |
| 284 | Ctri, 2021 | Comparision of dexmedetomidine versus midazolam and fentanyl combination on post-operative psychomotor functions and recovery | https://trialsearch.who.int/Trial2.aspx?TrialID=CTRI/2021/03/032061. 2021. | Excluded | Based on title and abstract |
| 285 | Ctri, 2022 | Comparison of effects of dexmedetomidine and midazolam in fragmented sleep related glucose changes in post operative ICU patients | https://trialsearch.who.int/Trial2.aspx?TrialID=CTRI/2022/01/039570. 2022. | Excluded | Based on title and abstract |
| 286 | Ctri, 2022 | It is the study done in spinal surgeries after completion of surgery. It is to assess the pain and sedation in post operative period for 24 hours .the study is to compare between two drugs | https://trialsearch.who.int/Trial2.aspx?TrialID=CTRI/2022/07/044302. 2022. | Excluded | Based on title and abstract |
| 287 | Ctri, 2022 | Opioid versus alternative drug for pain management during brain surgery | https://trialsearch.who.int/Trial2.aspx?TrialID=CTRI/2022/09/045705. 2022. | Excluded | Based on title and abstract |
| 288 | Ctri, 2022 | To make dental treatment easy without any anxiety for children with Intranasal spray of Dexmedetomidine/Midazolam | https://trialsearch.who.int/Trial2.aspx?TrialID=CTRI/2022/07/044010. 2022. | Excluded | Based on title and abstract |
| 289 | Cui Q, 2023 | Intraoperative infusion of dexmedetomidine for prevention of postoperative delirium in elderly patients undergoing craniotomy: a protocol of randomised clinical trial | BMJ Open. 2023; 13(1):e063976. | Excluded | Based on title and abstract |
| 290 | Cusimano JM, 2019 | Factors associated with delirium in surgical intensive care unit patients treated with supplemental melatonin: A case-cohort study | Clinical Neuropharmacology. 2019; 42(3):67-72. | Excluded | Based on title and abstract |
| 291 | Da SL, 2008 | Emergence agitation in pediatric anesthesia: Current features | Jornal de Pediatria. 2008; 84(2):107-113. | Excluded | Based on title and abstract |
| 292 | Dakin J, 2010 | Sleep-disordered breathing and anaesthesia in the morbidly obese | Current Anaesthesia and Critical Care. 2010; 21(1):24-30. | Excluded | Based on title and abstract |
| 293 | Date A, 2020 | Differences between natural sleep and the anesthetic state | Future Science OA. 2020; 6(10). | Excluded | Based on title and abstract |
| 294 | Dave NM, 2019 | Premedication and induction of anaesthesia in paediatric patients | Indian Journal of Anaesthesia. 2019; 63(9):713-720. | Excluded | Based on title and abstract |
| 295 | Davy A, 2017 | Dexmedetomidine and general anesthesia: a narrative literature review of its major indications for use in adults undergoing non-cardiac surgery | Minerva Anestesiol. 2017; 83(12):1294-1308. | Excluded | Based on title and abstract |
| 296 | Dawes J, 2014 | Identifying a rapid bolus dose of dexmedetomidine (ED50) with acceptable hemodynamic outcomes in children | Paediatric Anaesthesia. 2014; 24(12):1260-1267. | Excluded | Based on title and abstract |
| 297 | Deady KA, 2007 | Clinical pearls for procedural sedation | Orthopedics. 2007; 30(7):527-531. | Excluded | Based on title and abstract |
| 298 | DeHart AN, 2019 | Perioperative interdisciplinary approach for reduction of opioid use in pediatric tonsillectomy: Protocol using dexmedetomidine and bupivicaine as adjunct agents | Am J Otolaryngol. 2019; 40(3):382-388. | Excluded | Based on title and abstract |
| 299 | Deiner S, 2009 | Parkinson's disease and deep brain stimulator placement | Anesthesiology Clinics. 2009; 27(3):391-415. | Excluded | Based on title and abstract |
| 300 | Del LR, 2020 | Postoperative analgesia time in dogs submitted to mastectomy and anesthetized with tumescent solutions of lidocaine or ropivacaine | Acta Scientiae Veterinariae. 2020; 48. | Excluded | Based on title and abstract |
| 301 | Deng CM, 2023 | Effect of intraoperative remimazolam on postoperative sleep quality in elderly patients after total joint arthroplasty: a randomized control trial | J Anesth. 2023; 37(4):511-521. | Excluded | Based on title and abstract |
| 302 | Deng J, 2021 | A capnography and transcutaneous CO2 profile of bariatric patients during early postoperative period after opioid-sparing anesthesia | Surgery for Obesity and Related Diseases. 2021; 17(5):963-967. | Excluded | Based on title and abstract |
| 303 | Dervan LA, 2022 | Sleep architecture in mechanically ventilated pediatric ICU patients receiving goal-directed, dexmedetomidine- and opioid-based sedation | Journal of Pediatric Intensive Care. 2022; 11(1):32-40. | Excluded | Based on title and abstract |
| 304 | Devabhakthuni S, 2012 | Analgosedation: A paradigm shift in intensive care unit sedation practice | Annals of Pharmacotherapy. 2012; 46(4):530-540. | Excluded | Based on title and abstract |
| 305 | Devlin J, 2019 | ICU sleep management practices across 6 states prior to 2018 SCCM PADIS practice guideline release | Critical Care Medicine. 2019; 47(1). | Excluded | Based on title and abstract |
| 306 | Devlin JW, 2010 | Adverse drug events associated with the use of analgesics, sedatives, and antipsychotics in the intensive care unit | Critical Care Medicine. 2010; 38(6 SUPPL.):S231-S243. | Excluded | Based on title and abstract |
| 307 | Devlin JW, 2011 | Antipsychotics for the prevention and treatment of delirium in the intensive care unit: What is their role? | Harvard Review of Psychiatry. 2011; 19(2):59-67. | Excluded | Based on title and abstract |
| 308 | Devlin JW, 2012 | Pharmacologic prevention and treatment of delirium in critically ill and non-critically ill hospitalised patients: A review of data from prospective, randomised studies | Best Practice and Research: Clinical Anaesthesiology. 2012; 26(3):289-309. | Excluded | Based on title and abstract |
| 309 | Dholakia C, 2007 | The impact of perioperative dexmedetomidine infusion on postoperative narcotic use and duration of stay after laparoscopic bariatric surgery | J Gastrointest Surg. 2007; 11(11):1556-1559. | Excluded | Based on title and abstract |
| 310 | Dijemeni E, 2018 | Drug-induced sedation endoscopy: data capture, data analysis and sedation administration | European Archives of Oto-Rhino-Laryngology. 2018; 275(1):309-310. | Excluded | Based on title and abstract |
| 311 | Dillon RC, 2016 | Dexmedetomidine: A novel approach to treating refractory adrenergic crisis in familial dysautonomia | Clinical Autonomic Research. 2016; 26(5):368-369. | Excluded | Based on title and abstract |
| 312 | Dinsmore J, 2007 | Anaesthesia for elective neurosurgery | British Journal of Anaesthesia. 2007; 99(1):68-74. | Excluded | Based on title and abstract |
| 313 | Doan V, 2022 | Propofol versus remifentanil sedation for transcatheter aortic valve replacement: A single academic center experience | Journal of Cardiothoracic and Vascular Anesthesia. 2022; 36(1):103-108. | Excluded | Based on title and abstract |
| 314 | Dong CS, 2016 | The optimal dose of dexmedetomidine added to an sufentanil-based analgesic regimen for postoperative pain control in spine surgery A probit analysis study | Medicine (United States). 2016; 95(39). | Excluded | Based on title and abstract |
| 315 | Dong CS, 2017 | Effect of dexmedetomidine combined with sufentanil for post- thoracotomy intravenous analgesia: a randomized, controlled clinical study | BMC anesthesiology. 2017; 17(1):33. | Excluded | Based on title and abstract |
| 316 | Dong R, 2018 | Safety and efficacy of applying sufficient analgesia combined with a minimal sedation program as an early antihypertensive treatment for spontaneous intracerebral hemorrhage: A randomized controlled trial | Trials. 2018; 19(1):607. | Excluded | Based on title and abstract |
| 317 | Douma A, 2019 | Opioid free anaesthesia with ra techniques: What is the concept? | Regional Anesthesia and Pain Medicine. 2019; 44(10):A41-A43. | Excluded | Based on title and abstract |
| 318 | Downing F, 2018 | Anaesthesia of brachycephalic dogs | Journal of Small Animal Practice. 2018; 59(12):725-733. | Excluded | Based on title and abstract |
| 319 | Du D, 2022 | Combined sevoflurane-dexmedetomidine and nerve blockade on post-surgical serum oxidative stress biomarker levels in thyroid cancer patients | World Journal of Clinical Cases. 2022; 10(10):3027-3034. | Excluded | Based on title and abstract |
| 320 | Du J, 2023 | Development and validation of a nomogram for postoperative sleep disturbance in adults: a prospective survey of 640 patients undergoing spinal surgery | BMC Anesthesiol. 2023; 23(1):154. | Excluded | Based on title and abstract |
| 321 | Dzierba A, 2015 | A national evaluation of ICU delirium identification, prevention and treatment practices | Critical Care Medicine. 2015; 43(12):148. | Excluded | Based on title and abstract |
| 322 | Ebenezer V, 2013 | Conscious sedation after administration of drugs in dentistry | International Journal of Pharma and Bio Sciences. 2013; 4(2):P168-P172. | Excluded | Based on title and abstract |
| 323 | Eberl S, 2016 | Satisfaction and safety using dexmedetomidine or propofol sedation during endoscopic oesophageal procedures | European Journal of Anaesthesiology. 2016; 33(9):631-637. | Excluded | Based on title and abstract |
| 324 | Ebert T, 2004 | Dexmedetomidine: Another arrow for the clinician's quiver | Anesthesiology. 2004; 101(3):568-570. | Excluded | Based on title and abstract |
| 325 | Edenharter G, 2018 | Drug-induced sleep endoscopy | Somnologie. 2018; 22(2):112-116. | Excluded | Based on title and abstract |
| 326 | Egbuta C, 2021 | Current state of analgesia and sedation in the pediatric intensive care unit | Journal of Clinical Medicine. 2021; 10(9). | Excluded | Based on title and abstract |
| 327 | Ehsan Z, 2016 | The effects of anesthesia and opioids on the upper airway: A systematic review | Laryngoscope. 2016; 126(1):270-284. | Excluded | Based on title and abstract |
| 328 | Eipe N, 2022 | Perioperative pain management in bariatric anesthesia | Saudi J Anaesth. 2022; 16(3):339-346. | Excluded | Based on title and abstract |
| 329 | Eizaga R, 2021 | Dexmedetomidine & perioperative analgesia in children | Rev Esp Anestesiol Reanim (Engl Ed). 2021: S0034-9356. | Excluded | Based on title and abstract |
| 330 | Eizaga R, 2022 | Dexmedetomidine and perioperative analgesia in children | Rev Esp Anestesiol Reanim (Engl Ed). 2022; 69(8):487-492. | Excluded | Based on title and abstract |
| 331 | Elkalla RS, 2020 | Respiratory and hemodynamic effects of three different sedative regimens for drug induced sleep endoscopy in sleep apnea patients. A prospective randomized study | Minerva Anestesiol. 2020; 86(2):132-140. | Excluded | Based on title and abstract |
| 332 | Elmore B, 2015 | Pain management following thoracic surgery | Thoracic Surgery Clinics. 2015; 25(4):393-409. | Excluded | Based on title and abstract |
| 333 | Elmoutaz MH, 2018 | Efficacy of dexmedetomidine versus ketofol for sedation of postoperative mechanically ventilated patients with obstructive sleep apnea | Crit Care Res Pract. 2018; 2018:1015054. | Excluded | Based on title and abstract |
| 334 | Elsharkawy RA, 2017 | Intra-articular versus intravenous administration of dexmedetomidine in arthroscopic knee surgeries under local anesthesia: a prospective randomized study | Egyptian journal of anaesthesia. 2017; (no pagination). | Excluded | Based on title and abstract |
| 335 | Elvir L, 2010 | Postoperative pain management after ambulatory surgery: role of multimodal analgesia | Anesthesiology Clinics. 2010; 28(2):217-224. | Excluded | Based on title and abstract |
| 336 | Erickson A, 2021 | A review of pre-appointment medications to reduce fear and anxiety in dogs and cats at veterinary visits | Canadian Veterinary Journal. 2021; 62(9):952-960. | Excluded | Based on title and abstract |
| 337 | Esaki M, 2017 | Conscious sedation for endoscopic submucosal ressection by using dexmedetomidine | United European Gastroenterology Journal. 2017; 5(5):A211. | Excluded | Based on title and abstract |
| 338 | Euctr AT, 2011 | The effect and safety of dexmedetomidine as an additive to ropivacaine for interscalene brachial plexus blocks (regional anesthesia) for shoulder surgery | https://trialsearch.who.int/Trial2.aspx?TrialID=EUCTR2011-002175-42-AT. 2011. | Excluded | Based on title and abstract |
| 339 | Euctr BE, 2017 | Low dose dexmedetomidine and delirium after cardiac surgery | https://trialsearch.who.int/Trial2.aspx?TrialID=EUCTR2017-002007-97-BE. 2017. | Excluded | Based on title and abstract |
| 340 | Euctr DE, 2013 | Neuroprotection with dexmedetomidine in patients undergoing elective cardiac or abdominal surgery | https://trialsearch.who.int/Trial2.aspx?TrialID=EUCTR2013-000823-15-DE. 2013. | Excluded | Based on title and abstract |
| 341 | Euctr DK, 2021 | The effects of combined use of dexamethasone (corticosteroid) and dexmedetomidine (sedative) on a nerve block in patients undergoing surgery of the bones in the foot and ankle | https://trialsearch.who.int/Trial2.aspx?TrialID=EUCTR2021-000429-28-DK. 2021. | Excluded | Based on title and abstract |
| 342 | Euctr SE, 2013 | The effect of dexmedetomidine in combination with local anaesthetics after application around nerves in the abdominal wall in pediatric patients | https://trialsearch.who.int/Trial2.aspx?TrialID=EUCTR2013-000122-60-SE. 2013. | Excluded | Based on title and abstract |
| 343 | Fahim MR, 2005 | The addition of sufentanil, tramadol or dexmedetomidine to lidocaine for intravenous regional anaesthesia | Egyptian Journal of Anaesthesia. 2005; 21(4):283-288. | Excluded | Based on title and abstract |
| 344 | Fan Q, 2022 | Transcutaneous electrical acupoint stimulation combined with auricular acupressure reduces postoperative delirium among elderly patients following major abdominal surgery: A randomized clinical trial | Front Med. 2022; 9:855296 . | Excluded | Based on title and abstract |
| 345 | Farias FC, 2019 | Sleep endoscopy with dexmedetomidine in the evaluation of patients with Sleep Apnea | Pakistan Journal of Medical and Health Sciences. 2021; 15(2):288-292. | Excluded | Based on title and abstract |
| 346 | Farooq S, 2021 | Comparative behaviour of two different doses of dexmedetomidine in intrathecal anesthesia | Sleep Science. 2019; 12:21. | Excluded | Based on title and abstract |
| 347 | Fattah K, 2022 | Opioid sparing analgesia: Continuous intraoperative infusion of dexmedetomidine versus lidocaine for intracranial surgeries in children: A double-blind randomized clinical trial | Egyptian Journal of Anaesthesia. 2022; 38(1):158-165. | Excluded | Based on title and abstract |
| 348 | Faustino TN, 2022 | Effectiveness of combined non-pharmacological interventions in the prevention of delirium in critically ill patients: A randomized clinical trial | Journal of Critical Care. 2022; 68:114-120. | Excluded | Based on title and abstract |
| 349 | Fazal AA, 2019 | The perfect storm: Dynamic left ventricular outflow tract obstruction and the use of point of care cardiac ultrasound to guide intraoperative management | Journal of Clinical Anesthesia. 2019; 57:75-76. | Excluded | Based on title and abstract |
| 350 | Fernandes ML, 2015 | Sedation for electroencephalography with dexmedetomidine or chloral hydrate: A comparative study on the qualitative and quantitative electroencephalogram pattern | Journal of Neurosurgical Anesthesiology. 2015; 27(1):21-25. | Excluded | Based on title and abstract |
| 351 | Fernandes ML, 2016 | Sedation with dexmedetomidine for conducting electroencephalogram in a patient with Angelman syndrome: A case report | Brazilian Journal of Anesthesiology. 2016; 66(2):212-214. | Excluded | Based on title and abstract |
| 352 | Filbey WA, 2014 | Eszopiclone and dexmedetomidine depress ventilation in obese rats with features of metabolic syndrome | Sleep. 2014; 37(5):871-880. | Excluded | Based on title and abstract |
| 353 | Filos KS,1 999 | Current concepts and practice in postoperative pain management: Need for a change? | European Surgical Research. 1999; 31(2):97-107. | Excluded | Based on title and abstract |
| 354 | Fiore M, 2019 | Perioperative dexmedetomidine infusion, as opioid-sparing strategy, in patients undergoing general anesthesia: A systematic review protocol | Open Anesthesia Journal. 2019; 13(1):139-143. | Excluded | Based on title and abstract |
| 355 | Flanagan E, 2011 | He's taking how much dilaudid? Dexmedetomidine: A novel approach to refractory symptom management (338) (advanced) | Journal of Pain and Symptom Management. 2011; 41(1):214-215. | Excluded | Based on title and abstract |
| 356 | Fondeur J, 2022 | Dexmedetomidine in prevention of postoperative delirium: A systematic review | Cureus. 2022; 14(6):e25639. | Excluded | Based on title and abstract |
| 357 | Fong CY, 2017 | Chloral hydrate as a sedating agent for neurodiagnostic procedures in children | Cochrane Database of Systematic Reviews. 2017; 2017(11). | Excluded | Based on title and abstract |
| 358 | Foster CH, 2019 | Awake craniotomy in glioma surgery: Is it necessary? | Journal of Neurosurgical Sciences. 2019; 63(2):162-178. | Excluded | Based on title and abstract |
| 359 | Fourie CE, 2003 | Preventing ICU delirium | Southern African Journal of Critical Care. 2003; 19(2):42-45. | Excluded | Based on title and abstract |
| 360 | Francos GC, 2021 | McCune albright syndrome: Facing a scary airway | Anesthesia and Analgesia. 2021; 133(3 SUPPL 2):401-402. | Excluded | Based on title and abstract |
| 361 | Franks NP, 2021 | The inescapable drive to sleep: Overlapping mechanisms of sleep and sedation | Science. 2021; 374(6567):556-559. | Excluded | Based on title and abstract |
| 362 | Freiherr V, 2016 | Use of dexmedetomidine continuous subcutaneous infusion (CSCI) in a palliative care service in Ireland | Palliative Medicine. 2016; 30(6):NP85. | Excluded | Based on title and abstract |
| 363 | Friedlander AH, 2018 | The Importance of recognizing an association between obstructive sleep apnea and post-traumatic stress disorder | Journal of Oral and Maxillofacial Surgery. 2018; 76(11):2251-2253. | Excluded | Based on title and abstract |
| 364 | Friedman NR, 2017 | The current state of pediatric drug-induced sleep endoscopy | Laryngoscope. 2017; 127(1):266-272. | Excluded | Based on title and abstract |
| 365 | Friedman NR, 2022 | Tonsillectomy for obstructive sleep-disordered breathing: should they stay, or could they go? | Laryngoscope. 2022; 132(8):1675-1681. | Excluded | Based on title and abstract |
| 366 | Friese RS, 2008 | Sleep and recovery from critical illness and injury: A review of theory, current practice, and future directions | Critical Care Medicine. 2008; 36(3):697-705. | Excluded | Based on title and abstract |
| 367 | Fritsch G, 2014 | Dexmedetomidine added to ropivacaine extends the duration of interscalene brachial plexus blocks for elective shoulder surgery when compared with ropivacaine alone: A single-center, prospective, triple-blind, randomized controlled trial | Regional Anesthesia and Pain Medicine. 2014; 39(1):37-47. | Excluded | Based on title and abstract |
| 368 | Frontera JA, 2011 | Delirium and sedation in the ICU | Neurocritical Care. 2011; 14(3):463-474. | Excluded | Based on title and abstract |
| 369 | Frost EAM, 2007 | Anesthesia in the patient for awake craniotomy | Current Opinion in Anaesthesiology. 2007; 20(4):331-335. | Excluded | Based on title and abstract |
| 370 | ftypy RBR, 2018 | Pain after two different multimodal anesthesia techniques in obesity surgery | https://trialsearch.who.int/Trial2.aspx?TrialID=RBR-9ftypy. 2018. | Excluded | Based on title and abstract |
| 371 | Fujita T, 2009 | Better sleep in the ICU for preventing postoperative delirium | Journal of the American College of Surgeons. 2009; 209(6):792-793. | Excluded | Based on title and abstract |
| 372 | Fumagalli R, 2006 | Postoperative sedation and analgesia after pediatric liver transplantation | Transplantation Proceedings. 2006; 38(3):841-843. | Excluded | Based on title and abstract |
| 373 | Gadsden J, 2005 | Post-cesarean delivery analgesia | Anesthesia and Analgesia. 2005; 101(5 SUPPL.):S62-S69. | Excluded | Based on title and abstract |
| 374 | Gan TJ, 2006 | Pharmacokinetic and pharmacodynamic characteristics of medications used for moderate sedation | Clinical Pharmacokinetics. 2006; 45(9):855-869. | Excluded | Based on title and abstract |
| 375 | Gandolfi JV, 2020 | The effects of melatonin supplementation on sleep quality and assessment of the serum melatonin in ICU patients: A randomized controlled trial | Critical Care Medicine. 2020; 48(12):E1286-E1293. | Excluded | Based on title and abstract |
| 376 | Garavaglia M, 2013 | Dexmedetomidine for awake craniotomy in complex patients | Canadian Journal of Anesthesia. 2013; 60(1):S67. | Excluded | Based on title and abstract |
| 377 | Garavaglia M, 2013 | Dexmedetomidine for complex patients undergoing awake craniotomy | Anesthesia and Analgesia. 2013; 116. | Excluded | Based on title and abstract |
| 378 | Garcia A, 2020 | Postoperative effect of midazolam premedication in pediatric patients with obstructive sleep apnea | Clinical Pharmacology in Drug Development. 2020; 9(SUPPL 2):51. | Excluded | Based on title and abstract |
| 379 | Gardner BM, 2014 | Obstructive sleep apnoea | South African Family Practice. 2014; 56(2):S41-S44. | Excluded | Based on title and abstract |
| 380 | Garg N, 2016 | Comparison of small dose ketamine and dexmedetomidine infusion for postoperative analgesia in spine surgery - A prospective randomized double-blind placebo controlled study | Journal of Neurosurgical Anesthesiology. 2016; 28(1):27-31. | Excluded | Based on title and abstract |
| 381 | Garner S, 2022 | Delirium occurrence in a fentanyldriven analgosedation protocol in the intensive care unit | Critical Care Medicine. 2022; 50(1 SUPPL):401. | Excluded | Based on title and abstract |
| 382 | Garrity AG, 2015 | Dexmedetomidine-induced sedation does not mimic the neurobehavioral phenotypes of sleep in sprague dawley rat | Sleep. 2015; 38(1):73-84. | Excluded | Based on title and abstract |
| 383 | Gasper PL, 2013 | The use of dexmedetomidine HCL as a safe, efficacious, and cost-effective alternative to general anesthesia during arctic Front庐 balloon cryoablation for atrial fibrillation | Heart Rhythm. 2013; 10(5):S105-S106. | Excluded | Based on title and abstract |
| 384 | Gaszynski T, 2006 | Dexmedetomidine for sedation in Prader-Willi syndrome. Case report | Anestezjologia Intensywna Terapia. 2006; 38(2):75-77. | Excluded | Based on title and abstract |
| 385 | Gau TP, 2020 | Multimodal anesthesia management of a morbidly obese spinal muscular atrophy patient with obstructive sleep apnea | Kaohsiung Journal of Medical Sciences. 2020; 36(1):73-74. | Excluded | Based on title and abstract |
| 386 | Georgopoulos D, 2021 | Effects of sedatives on sleep architecture measured with odds ratio product in critically ill patients | Crit Care Explor. 2021; 3(8):e0503. | Excluded | Based on title and abstract |
| 387 | Gernsback JE, 2018 | Who needs sleep? An analysis of patient tolerance in awake craniotomy | World Neurosurgery. 2018; 118:e842-e848. | Excluded | Based on title and abstract |
| 388 | Ghai B, 2022 | Opioid sparing strategies for perioperative pain management other than regional anaesthesia: A narrative review | Journal of Anaesthesiology Clinical Pharmacology. 2022; 38(1):3-10. | Excluded | Based on title and abstract |
| 389 | Ghosn A, 2017 | Evaluation of the effect of the addition of dexmedetomidine in the axillary block during upper limb surgery | Regional Anesthesia and Pain Medicine. 2017; 42(5):e196-e197. | Excluded | Based on title and abstract |
| 390 | Gomez B, 2016 | Assessment of postoperative psychological outcomes associated with awake craniotomy facilitated by a dexmedetomidine-based anesthetic protocol | Journal of Neurosurgical Anesthesiology. 2016; 28(2):S38. | Excluded | Based on title and abstract |
| 391 | Goncalves D, 2021 | Regional anesthesia for correction of clavicle fracture in a polytrauma patient diagnosed with severe obstructive sleep apnea (osa) | Journal of Biological Regulators and Homeostatic Agents. 2020; 34(6):2171-2175. | Excluded | Based on title and abstract |
| 392 | Gong HY, 2020 | Effects of dexmedetomidine combined with propofol on peri-anesthesia hemodynamics and postoperative sedation and analgesia in patients undergoing radical esophagectomy | Regional Anesthesia and Pain Medicine. 2021; 70(SUPPL 1):A80. | Excluded | Based on title and abstract |
| 393 | Goo BW, 2017 | A comparison of upper airway patency by dexmedetomidine and propofol used for intraoperative sedation in patients with obstructive sleep apnea | Sleep medicine. Conference: 14th world sleep congress. Czech republic. 2017; 40(Supplement 1):e234. | Excluded | Based on title and abstract |
| 394 | Goudra B, 2019 | Big sleep: beyond propofol sedation during GI endoscopy | Digestive Diseases and Sciences. 2019; 64(1). | Excluded | Based on title and abstract |
| 395 | Grace E, 2023 | Association between postoperative delirium and long-term subjective cognitive decline in older patients undergoing cardiac surgery: A secondary analysis of the minimizing intensive care unit neurological dysfunction with dexmedetomidine-induced sleep trial | J Cardiothorac Vasc Anesth. 2023; 37(9):1700-1706. | Excluded | Based on title and abstract |
| 396 | Grassi L, 2015 | Management of delirium in palliative care: a review | Current Psychiatry Reports. 2015; 17(3). | Excluded | Based on title and abstract |
| 397 | Grawe ES, 2013 | Sedation of critically ill patients undergoing mechanical ventilation | International Anesthesiology Clinics. 2013; 51(2):62-80. | Excluded | Based on title and abstract |
| 398 | Gray M, 2017 | The management of unplanned erection during endoscopic urological surgery | Current Urology. 2017; 10(3):113-117. | Excluded | Based on title and abstract |
| 399 | Greenhalgh DL, 2008 | Sedation during ophthalmic surgery | European Journal of Anaesthesiology. 2008; 25(9):701-707. | Excluded | Based on title and abstract |
| 400 | Grewal G, 2019 | Obesity and obstructive sleep apnea in the ambulatory patient | Anesthesiology Clinics. 2019; 37(2):215-224. | Excluded | Based on title and abstract |
| 401 | Grig M, 2018 | Reducing postoperative opioid use in children after adenotonsillectomy | Anesthesia and analgesia. 2018; 126(4):559‐. | Excluded | Based on title and abstract |
| 402 | Grigg-Damberger MM, 2022 | Sleep spindles and K-complexes are favorable prognostic biomarkers in critically ill patients | Journal of Clinical Neurophysiology. 2022; 39(5):372-382. | Excluded | Based on title and abstract |
| 403 | Guenther U, 2011 | Delirium in the postanaesthesia period | Current Opinion in Anaesthesiology. 2011; 24(6):670-675. | Excluded | Based on title and abstract |
| 404 | Guldenmund P, 2017 | Brain functional connectivity differentiates dexmedetomidine from propofol and natural sleep | British Journal of Anaesthesia. 2017; 119(4):674-684. | Excluded | Based on title and abstract |
| 405 | Gumus H, 2015 | Comparison of effects of different dexmedetomidine and chloral hydrate doses used in sedation on electroencephalography in pediatric patients | Journal of Child Neurology. 2015; 30(8):983-988. | Excluded | Based on title and abstract |
| 406 | Guo B, 2021 | Effects of dexmedetomidine on postoperative cognitive function of sleep deprivation rats based on changes in inflammatory response | Bioengineered. 2021; 12(1):7920-7928. | Excluded | Based on title and abstract |
| 407 | Ha SH, 2011 | Use of dexmedetomidine for awake crainiotomy | Korean Journal of Anesthesiology. 2011; 61(4):346-347. | Excluded | Based on title and abstract |
| 408 | Habib AS, 2005 | Role of analgesic adjuncts in postoperative pain management | Anesthesiology Clinics of North America. 2005; 23(1):85-107. | Excluded | Based on title and abstract |
| 409 | Haenecour A, 2015 | Prolonged dexmedetomidine infusion and drug withdrawal in critically ill children | Critical Care. 2015; 19:S169-S170. | Excluded | Based on title and abstract |
| 410 | Hamid AMA, 2017 | Effect of intranasal dexmedetomidine on emergence agitation after sevoflurane anesthesia in children undergoing tonsillectomy and/or adenoidectomy | Saudi Journal of Anaesthesia. 2017; 11(2):137-143. | Excluded | Based on title and abstract |
| 411 | Hamilton TB, 2020 | Adenotonsillectomy and postoperative respiratory adverse events: A retrospective study | Laryngoscope Investigative Otolaryngology. 2020; 5(1):168-174. | Excluded | Based on title and abstract |
| 412 | Hammer GB, 2005 | Prolonged infusion of dexmedetomidine for sedation following tracheal resection | Paediatr Anaesth. 2005; 15(7):616-620. | Excluded | Based on title and abstract |
| 413 | Hammer GB, 2009 | Sedation and analgesia in the pediatric intensive care unit following laryngotracheal reconstruction | Paediatric Anaesthesia. 2009; 19(SUPPL. 1):166-179. | Excluded | Based on title and abstract |
| 414 | Han C, 2014 | Application of dexmedetomidine combined with ropivacaine in the cesarean section under epidural anesthesia | National Medical Journal of China. 2014; 94(44):3501-3505. | Excluded | Based on title and abstract |
| 415 | Han C, 2017 | Incidence and risk factors of chronic pain following hysterectomy among Southern Jiangsu Chinese Women | BMC Anesthesiol. 2017; 17(1):103. | Excluded | Based on title and abstract |
| 416 | Han G, 2014 | A randomized study of intranasal vs. intravenous infusion of dexmedetomidine in gastroscopy | International journal of clinical pharmacology and therapeutics. 2014; 52(9):756‐761. | Excluded | Based on title and abstract |
| 417 | Hanna AH, 2012 | Challenges in paediatric ambulatory anesthesia | Curr Opin Anaesthesiol. 2012; 25(3):315-320. | Excluded | Based on title and abstract |
| 418 | Hannallah MS, 2012 | Evaluation of dexmedetomidine/profofol (D/P) combination anesthesia for patients with obstructive sleep apnea (OSA) characteristics during upper gastrointestinal (GI) endoscopy | Anesthesia and Analgesia. 2012; 114(5):S02. | Excluded | Based on title and abstract |
| 419 | Harper P, 2015 | Update on ENT anaesthesia | Anaesthesia and Intensive Care Medicine. 2015; 16(12):635-640. | Excluded | Based on title and abstract |
| 420 | Harrison TK, 2018 | Perioperative considerations for the patient with opioid use disorder on buprenorphine, methadone, or naltrexone maintenance therapy | Anesthesiology Clinics. 2018; 36(3):345-359. | Excluded | Based on title and abstract |
| 421 | Hau V, 2021 | Anesthesia management for ENT surgery in a child with X-linked pyruvate kinase deficiency | Paediatr Anaesth. 2021; 31(4):499-500. | Excluded | Based on title and abstract |
| 422 | Hayashi Y, 1996 | Hypnotic and analgesic effects of the α2-adrenergic agonist dexmedetomidine in morphine-tolerant rats | Anesthesia and Analgesia. 1996; 83(3):606-610. | Excluded | Based on title and abstract |
| 423 | Hayek SM, 2003 | Pharmacology of intrathecally administered agents for treatment of spasticity and pain | Seminars in Pain Medicine. 2003; 1(4):238-253. | Excluded | Based on title and abstract |
| 424 | Hayhurst CJ, 2016 | Intensive care unit delirium: A review of diagnosis, prevention, and treatment | Anesthesiology. 2016; 125(6):1229-1241. | Excluded | Based on title and abstract |
| 425 | Hazkani I, 2021 | Hypoglossal nerve stimulation for treatment of severe refractory obstructive sleep apnea in children with down syndrome | Current Sleep Medicine Reports. 2021; 7(4):206-212. | Excluded | Based on title and abstract |
| 426 | He J, 2024 | Dexmedetomidine nasal administration improves perioperative sleep quality and neurocognitive deficits in elderly patients undergoing general anesthesia | BMC Anesthesiol. 2024; 24(1):42. | Excluded | Based on title and abstract |
| 427 | He S, 2018 | Outcomes of drug-induced sleep endoscopy-directed surgery for pediatric obstructive sleep apnea | Otolaryngology - Head and Neck Surgery (United States). 2018; 158(3):559-565. | Excluded | Based on title and abstract |
| 428 | Heard C, 2014 | Does dexmedetomidine cause less airway collapse than propofol when used for deep sedation | Anesthesia and Analgesia. 2014; 118(5):S259. | Excluded | Based on title and abstract |
| 429 | Hefni AF, 2022 | Comparing effect of adding ketamine versus dexmedetomidine to bupivacaine in pecs- block on postoperative pain control in patients undergoing breast surgery | Clinical Journal of Pain. 2022; 38(9):568-574. | Excluded | Based on title and abstract |
| 430 | Henrichs BM, 2012 | Is that snoring something to worry about? Anesthetic implications for obstructive sleep apnea | AANA Journal. 2012; 80(5):393-401. | Excluded | Based on title and abstract |
| 431 | Hernandez-Avalos I, 2021 | Neurobiology of anesthetic-surgical stress and induced behavioral changes in dogs and cats: A review | Veterinary World. 2021; 14(2):393-404. | Excluded | Based on title and abstract |
| 432 | Heybati K, 2022 | Efficacy of dexmedetomidine versus propofol sedation in critically ill adults requiring mechanical ventilation: A systematic review and meta-analysis of randomized trials | American Journal of Respiratory and Critical Care Medicine. 2022; 205(1). | Excluded | Based on title and abstract |
| 433 | Hight D, 2022 | Consciousness and the outside world: is there anyone listening? | British Journal of Anaesthesia. 2022; 128(6):895-897. | Excluded | Based on title and abstract |
| 434 | Hippard HK, 2014 | Preservation of microelectrode recordings with non-GABAergic drugs during deep brain stimulator placement in children | J Neurosurg Pediatr. 2014; 14(3):279-286. | Excluded | Based on title and abstract |
| 435 | Hofer RE, 2005 | Anesthesia for a patient with morbid obesity using dexmedetomidine without narcotics | Can J Anaesth. 2005; 52(2):176-180. | Excluded | Based on title and abstract |
| 436 | Hofherr ML, 2020 | Dexmedetomidine: A novel strategy for patients with intractable pain, opioid-induced hyperalgesia, or delirium at the end of life | Journal of Palliative Medicine. 2020; 23(11):1515-1517. | Excluded | Based on title and abstract |
| 437 | Hollmann MW, 2021 | Opioid-free anaesthesia - Ways out of the opioid crisis and the future of perioperative patient management? | Anasthesiologie und Intensivmedizin. 2021; 62(12):553-562. | Excluded | Based on title and abstract |
| 438 | Hong B, 2019 | Prolonged analgesic duration of brachial plexus block by addition of dexamethasone to dexmedetomidine for sedation | Regional anesthesia and pain medicine. 2019; 44(10):A271‐A273. | Excluded | Based on title and abstract |
| 439 | Hong B, 2021 | The effect of intravenous dexamethasone and dexmedetomidine on analgesia duration of supraclavicular brachial plexus block: a randomized, four-arm, triple-blinded, placebo-controlled trial | Journal of personalized medicine. 2021; 11(12). | Excluded | Based on title and abstract |
| 440 | Hong H, 2021 | Impact of dexmedetomidine supplemented analgesia on delirium in patients recovering from orthopedic surgery: A randomized controlled trial | BMC Anesthesiol. 2021; 21(1):223. | Excluded | Based on title and abstract |
| 441 | Honorato CC, 2017 | Sedation during surgery for movement disorders and perioperative neurologic complications: An observational study comparing local anesthesia, remifentanil, and dexmedetomidine | World Neurosurgery. 2017; 101:114-121. | Excluded | Based on title and abstract |
| 442 | Hsieh SJ, 2013 | Can intensive care unit delirium be prevented and reduced? lessons learned and future directions | Annals of the American Thoracic Society. 2013; 10(6):648-656. | Excluded | Based on title and abstract |
| 443 | Hsu YW, 2004 | Dexmedetomidine pharmacodynamics: Part I - Crossover comparison of the respiratory effects of dexmedetomidine and remifentanil in healthy volunteers | Anesthesiology. 2004; 101(5):1066-1076. | Excluded | Based on title and abstract |
| 444 | Huang F, 2021 | Analgesia and patient comfort after enhanced recovery after surgery in uvulopalatopharyngoplasty: a randomised controlled pilot study | BMC Anesthesiology. 2021; 21(1). | Excluded | Based on title and abstract |
| 445 | Huang X, 2021 | Effect of dexmedetomidine on postoperative sleep quality: A systematic review | Drug Des Devel Ther. 2021; 15:2161-2170. | Excluded | Based on title and abstract |
| 446 | Huang X, 2022 | Ultrasound-guided erector spinae plane block improves analgesia after laparoscopic hepatectomy: a randomised controlled trial | British Journal of Anaesthesia. 2022; 129(3):445-453. | Excluded | Based on title and abstract |
| 447 | Huncke T, 2008 | The use of continuous positive airway pressure during an awake craniotomy in a patient with obstructive sleep apnea | J Clin Anesth. 2008; 20(4):297-299. | Excluded | Based on title and abstract |
| 448 | Huo S, 2022 | Intravenous immunoglobulins for treatment of severe COVID-19-related acute encephalopathy | Journal of Neurology. 2022; 269(8):4013-4020. | Excluded | Based on title and abstract |
| 449 | Huupponen E, 2008 | Electroencephalogram spindle activity during dexmedetomidine sedation and physiological sleep | Acta Anaesthesiologica Scandinavica. 2008; 52(2):289-294. | Excluded | Based on title and abstract |
| 450 | Hyzer JM, 2021 | Drug-induced sleep endoscopy findings in children with obstructive sleep apnea with vs without obesity or down syndrome | JAMA Otolaryngology - Head and Neck Surgery. 2021; 147(2):175-181. | Excluded | Based on title and abstract |
| 451 | Iakovou A, 2013 | Update on sedation in the critical care unit | Open Critical Care Medicine Journal. 2013; 6(SPEC. ISSUE.1):66-79. | Excluded | Based on title and abstract |
| 452 | Inagaki Y, 2009 | Comparison of dexmedetomidine with a combination of midazolam and ketamine in monitored anesthesia care for facial aesthetic surgery | European Journal of Anaesthesiology. 2009; 26:21-22. | Excluded | Based on title and abstract |
| 453 | Irct, 2017 | Comparison of the effects of two anesthetics on recovery profile of patients after hysterectomy operation | https://trialsearch.who.int/Trial2.aspx?TrialID=IRCT2017102731487N6. 2017. | Excluded | Based on title and abstract |
| 454 | Irct, 2017 | Effects of intravenous dexmedetomidine and intravenous apotel and combined of those (intravenous apodex) on postoperative emergence agitation & pain | https://trialsearch.who.int/Trial2.aspx?TrialID=IRCT2016092930049N1. 2017. | Excluded | Based on title and abstract |
| 455 | Irct, 2018 | A Comparative study between midazolam, dexmedetomidine and melatonin ?as oral premedication for pediatric sedation and ease of separation from ?their parents before anesthesia ? | https://trialsearch.who.int/Trial2.aspx?TrialID=IRCT20141209020258N77. 2018. | Excluded | Based on title and abstract |
| 456 | Irct, 2018 | Clinical trial the effect of local dexmedetomidine injection on post operative pain in patient with total knee arthroplasty surgery | https://trialsearch.who.int/Trial2.aspx?TrialID=IRCT20160430027677N7. 2018. | Excluded | Based on title and abstract |
| 457 | Irct, 2020 | Comparison of the effect of granisetron, ketamine, dexmedetomidine and lidocaine with fentanyl on sedation and analgesia for cystoscopy and bladder catheter tolerance | https://trialsearch.who.int/Trial2.aspx?TrialID=IRCT20141209020258N138. 2020. | Excluded | Based on title and abstract |
| 458 | Irct, 2020 | Survey the effect of mixture of two anesthetic drugs on control of postoperative pain of opioid dependent patients undergoing orthopedic ankle surgery | https://trialsearch.who.int/Trial2.aspx?TrialID=IRCT20121204011662N15. 2020. | Excluded | Based on title and abstract |
| 459 | Irct, 2021 | Comparison of oral premedication of midazolam, ketamine and dexmedetomidine in sedation and facilitation of separation of children from parents for induction of anesthesia | https://trialsearch.who.int/Trial2.aspx?TrialID=IRCT20211007052693N1. 2021. | Excluded | Based on title and abstract |
| 460 | Isaiah A, 2018 | Characterization of upper airway obstruction using cine MRI in children with residual obstructive sleep apnea after adenotonsillectomy | Sleep Med. 2018; 50:79-86. | Excluded | Based on title and abstract |
| 461 | Ishibashi C, 2013 | Safety and efficacy of dexmedetomidine in intubated, spontaneously breathing patients after endscopic submucosal dissection for esophageal or pharyngeal cancer | Anesthesia and Analgesia. 2013; 116. | Excluded | Based on title and abstract |
| 462 | Ishii D, 2020 | Safety and feasibility of the woven endobridge device deployment with monitored anesthesia care | Interventional Neuroradiology. 2020; 26(6):767-771. | Excluded | Based on title and abstract |
| 463 | Isomura H, 2008 | Combination of dexmedetomidine and midazolam is useful for postoperative sedation in healthy-young patients | Journal of japanese dental society of anesthesiology. 2008; 36(1):28‐32. | Excluded | Based on title and abstract |
| 464 | Iwasaki Y, 2010 | Retrospective evaluation of dexmedetomidine for postoperative sedation in patients for cerebral aneurysm surgery | Masui. 2010; 59(11):1396-1399. | Excluded | Based on title and abstract |
| 465 | Jackson P, 2015 | Delirium in critically ill patients | Critical Care Clinics. 2015; 31(3):589-603. | Excluded | Based on title and abstract |
| 466 | Jacobson JR, 2013 | Elevated liver enzymes associated with fluphenazine used to manage delirium symptoms in infants | Journal of Child and Adolescent Psychopharmacology. 2013; 23(7):513-514. | Excluded | Based on title and abstract |
| 467 | Jaideep CN, 2015 | Good vagal tone, a tourniquet and dexmedetomidine: Recipe for disaster | Indian Journal of Anaesthesia. 2015; 59(7):450-451. | Excluded | Based on title and abstract |
| 468 | Jain D, 2020 | In response to dexmedetomidine versus propofol at different sedation depths during drug-induced sleep endoscopy: A randomized trial | Laryngoscope. 2020; 130(7):E453. | Excluded | Based on title and abstract |
| 469 | Jayaraman L, 2013 | A comparative study to evaluate the effect of intranasal dexmedetomidine versus oral alprazolam as a premedication agent in morbidly obese patients undergoing bariatric surgery | J Anaesthesiol Clin Pharmacol. 2013; 29(2):179-182. | Excluded | Based on title and abstract |
| 470 | Ji K, 2022 | Pre- versus post-tonsillectomy intraoperative bupivacaine injection in the pediatric population: An age-and surgical indication-stratified analysis | Ear, Nose and Throat Journal. 2022; 101(8):518-525. | Excluded | Based on title and abstract |
| 471 | Jiang C, 2020 | Nalbuphine exhibited a better adjuvant than dexmedetomidine in supraclavicular brachial plexus block in youths | Clinical Neuropharmacology. 2020; 43(5):134-138. | Excluded | Based on title and abstract |
| 472 | Jiang N, 2021 | Optimized propofol anesthesia increases power of subthalamic neuronal activity in patients with parkinson's Disease Undergoing Deep Brain Stimulation | Neurology and Therapy. 2021; 10(2):785-802. | Excluded | Based on title and abstract |
| 473 | Jiang PS, 2003 | Recent advances in neuraxial analgesics | Progress in Anesthesiology. 2003; 17(6):91-102. | Excluded | Based on title and abstract |
| 474 | Jiang T, 2021 | Sedative and hypnotic effects of alpha-2 adrenergic agonists are mediated by adrenergic and glutamatergic neural populations | Journal of Neurosurgical Anesthesiology. 2021; 33(4):418. | Excluded | Based on title and abstract |
| 475 | Jin XB, 2021 | Effect of different modes of administration of dexmedetomidine combined with nerve block on postoperative analgesia in total knee arthroplasty | Pain Ther. 2021; 10(2):1649-1662. | Excluded | Based on title and abstract |
| 476 | Jin Z, 2021 | Safety of treatment options available for postoperative pain | Expert Opinion on Drug Safety. 2021; 20(5):549-559. | Excluded | Based on title and abstract |
| 477 | Jolly AS, 2007 | Ketamine-current uses and future perspectives | Journal of Anaesthesiology Clinical Pharmacology. 2007; 23(2):169-181. | Excluded | Based on title and abstract |
| 478 | Jones GM, 2020 | Foreword | Critical Care Nursing Quarterly. 2020; 43(2):107-108. | Excluded | Based on title and abstract |
| 479 | Jones OS, 2022 | Pediatric obstructive sleep apnea: Screening, diagnosis, outcomes, and disparities in care | International Anesthesiology Clinics. 2022; 60(2):66-73. | Excluded | Based on title and abstract |
| 480 | Joo EY, 2020 | Intramuscular dexmedetomidine and oral chloral hydrate for pediatric sedation for electroencephalography: A propensity score-matched analysis | Paediatric Anaesthesia. 2020; 30(5):584-591. | Excluded | Based on title and abstract |
| 481 | Jooste EH, 2017 | Phase IV, open-label, safety study Evaluating the use of dexmedetomidine in pediatric patients undergoing procedure-type sedation | Front Pharmacol. 2017. | Excluded | Based on title and abstract |
| 482 | Joseph BO, 2012 | Treatment of four psychiatric emergencies in the intensive care unit | Critical Care Medicine. 2012; 40(9):2662-2670. | Excluded | Based on title and abstract |
| 483 | Joshi GP, 2005 | Multimodal analgesia techniques and postoperative rehabilitation | Anesthesiology Clinics of North America. 2005; 23(1):185-202. | Excluded | Based on title and abstract |
| 484 | Joshi GP, 2005 | Multimodal analgesia techniques for ambulatory surgery | International Anesthesiology Clinics. 2005; 43(3):197-204. | Excluded | Based on title and abstract |
| 485 | Joshi GP, 2008 | Morphine-6-glucuronide, an active morphine metabolite for the potential treatment of post-operative pain | Current Opinion in Investigational Drugs. 2008; 9(7):786-799. | Excluded | Based on title and abstract |
| 486 | Joshi GP, 2017 | Anaesthetic considerations for ambulatory Bariatric surgery | Ambulatory Surgery. 2017; 23(2):29-30. | Excluded | Based on title and abstract |
| 487 | jRcts, 2020 | Dexmedetomidine supplemented analgesia for delirium and prolong survival after surgrey | https://trialsearch.who.int/Trial2.aspx?TrialID=JPRN-jRCTs021200016. 2020. | Excluded | Based on title and abstract |
| 488 | Jung D, 2012 | Comparison of dexmedetomidine with propofol for airway intervention in MRI sleep studies | Pediatric Radiology. 2012; 42:S270-S271. | Excluded | Based on title and abstract |
| 489 | Jurszewicz P, 2021 | Severe paediatric inflammatory multisystem syndrome temporally associated with SARS-CoV-2 in a 13-year-old boy - case report | Pediatria i Medycyna Rodzinna. 2021; 17(3):261-269. | Excluded | Based on title and abstract |
| 490 | Kabon B, 2003 | Therapeutic hypothermia | Best Practice and Research: Clinical Anaesthesiology. 2003; 17(4):551-568. | Excluded | Based on title and abstract |
| 491 | Kalra M, 2017 | Use of upper airway cine MRI to develop personalized treatment for obstructive sleep apnea in obese children | American Journal of Respiratory and Critical Care Medicine. 2017; 195. | Excluded | Based on title and abstract |
| 492 | Kamal M, 2016 | Efficacy of dexmedetomidine as an adjuvant to ropivacaine in pediatric caudal epidural block | Saudi J Anaesth. 2016; 10(4):384-389. | Excluded | Based on title and abstract |
| 493 | Kamath S, 2023 | Non-opioid versus opioid Peri-operative analgesia in neurosurgery (NOPAIN): study protocol for a multi-centric randomised controlled trial | Indian J Anaesth. 2023; 67(10):920-926. | Excluded | Based on title and abstract |
| 494 | Kameyama Y, 2012 | A case of congenital central hypoventilation syndrome | Journal of Anesthesia. 2012; 26(6):922-924. | Excluded | Based on title and abstract |
| 495 | Kanda S, 2019 | A case report: Protection of the air embolism during PVI for atrial fibrillation patient with severe OSAS | Journal of Arrhythmia. 2019; 35:214-215. | Excluded | Based on title and abstract |
| 496 | Kandil A, 2016 | Comparison of the combination of dexmedetomidine and ketamine to propofol or propofol/sevoflurane for drug-induced sleep endoscopy in children | Paediatr Anaesth. 2016; 26(7):742-751. | Excluded | Based on title and abstract |
| 497 | Kang D, 2019 | The correlation of heart rate between natural sleep and dexmedetomidine sedation | Korean J Anesthesiol. 2019; 72(2):164-168. | Excluded | Based on title and abstract |
| 498 | Kang H, 2012 | The effect of dexmedetomidine added to preemptive ropivacaine infiltration on post-operative pain after inguinal herniorrhaphy: A prospective, randomized, double-blind, placebo-controlled study | European Surgery - Acta Chirurgica Austriaca. 2012; 44(4):274-280. | Excluded | Based on title and abstract |
| 499 | Kapil S, 2017 | Desperate measures: Shunt insertion under local anesthesia | Journal of Neurosciences in Rural Practice. 2017; 8(5):153-154. | Excluded | Based on title and abstract |
| 500 | Kapoor MC, 2020 | Neurological dysfunction after cardiac surgery and cardiac intensive care admission: A narrative review part 2: Cognitive dysfunction after critical illness; potential contributors in surgery and intensive care; pathogenesis; and therapies to prevent/treat perioperative neurological dysfunction | Annals of Cardiac Anaesthesia. 2020; 23(4):391-400. | Excluded | Based on title and abstract |
| 501 | Karan SB, 2007 | Anesthesia and sleep medicine: an opportunity to be mutually informative? | Seminars in Anesthesia, Perioperative Medicine and Pain. 2007; 26(2):42-48. | Excluded | Based on title and abstract |
| 502 | Kawaai H, 2008 | Dexmedetomidine for postoperative management after sevoflurane anesthesia in children | Journal of Japanese Dental Society of Anesthesiology. 2008; 36(3):269-277. | Excluded | Based on title and abstract |
| 503 | Kaye AD, 2014 | Perioperative analgesia: Ever-changing technology and pharmacology | Best Practice and Research: Clinical Anaesthesiology. 2014; 28(1):3-14. | Excluded | Based on title and abstract |
| 504 | Kaye AD, 2017 | An update on nonopioids: Intravenous or oral analgesics for perioperative pain management | Anesthesiology Clinics. 2017; 35(2):e55-e71. | Excluded | Based on title and abstract |
| 505 | Kaye AD, 2017 | Pharmacologic considerations of anesthetic agents in pediatric patients: A comprehensive review | Anesthesiology Clinics. 2017; 35(2):e73-e94. | Excluded | Based on title and abstract |
| 506 | Kct,2017 | Comparision of IV dexamethasone and dexmedetomidine for prolonging the duration of analgesia after single shot interscalene brachial plexus block for arthroscopic shoulder surgery | http://www.who.int/trialsearch/Trial2.aspx?TrialID=KCT0002569. 2017. | Excluded | Based on title and abstract |
| 507 | Kct,2020 | Effect of intratheal fentanyl on sedation conducted by dexmedetomidne infusion | http://www.who.int/trialsearch/Trial2.aspx?TrialID=KCT0005162. 2020. | Excluded | Based on title and abstract |
| 508 | Keegan MT,2008 | Sedation in the neurologic intensive care unit | Current Treatment Options in Neurology. 2008; 10(2):111-125. | Excluded | Based on title and abstract |
| 509 | Keene DD,2011 | Acute pain management in trauma | Trauma. 2011; 13(3):167-179. | Excluded | Based on title and abstract |
| 510 | Keidan I,2014 | Electroencephalography for children with autistic spectrum disorder: A sedation protocol | Paediatr Anaesth. 2015; 25(2):200-5 | Excluded | Based on title and abstract |
| 511 | Kelly V,2022 | The woman who kept passing out | Current Psychiatry. 2022; 21(5):44-49. | Excluded | Based on title and abstract |
| 512 | Khalife M, 2020 | The challenge of opioid-free anesthesia | 2020(150):167-186 | Excluded | Based on title and abstract |
| 513 | Khandelwal A, 2019 | Phenytoin-induced excessive sedation during awake craniotomy: an unusual observation | Journal of Neurosurgical Anesthesiology. 2019; 31(1):87-88. | Excluded | Based on title and abstract |
| 514 | Khromava M, 2022 | Dexmedetomidine in the treatment of serotonin syndrome | Journal of Neurosurgical Anesthesiology. 2022; 34(4):468. | Excluded | Based on title and abstract |
| 515 | Kim KH, 2014 | Safe sedation and hypnosis using dexmedetomidine for minimally invasive spine surgery in a prone position | Korean J Pain. 2014; 27(4):313-320. | Excluded | Based on title and abstract |
| 516 | Kirkham EM, 2021 | Drug-Induced sleep endoscopy in children with positional obstructive sleep apnea | Otolaryngology - Head and Neck Surgery (United States). 2021; 164(1):191-198. | Excluded | Based on title and abstract |
| 517 | Kirkham EM, 2021 | Propofol versus dexmedetomidine during drug-induced sleep endoscopy (DISE) for pediatric obstructive sleep apnea | Sleep Breath. 2021; 25(2):757-765. | Excluded | Based on title and abstract |
| 518 | Kirksey MA, 2015 | Local anesthetic peripheral nerve block adjuvants for prolongation of analgesia: A systematic qualitative review | PLoS ONE. 2015; 10(9). | Excluded | Based on title and abstract |
| 519 | Kleiman AM, 2017 | Chronic poststernotomy pain: incidence, risk factors, treatment, prevention, and the anesthesiologist's role | Regional Anesthesia and Pain Medicine. 2017; 42(6):698-708. | Excluded | Based on title and abstract |
| 520 | Klimiec E, 2016 | Knowns and unknowns about delirium in stroke: A review | Cognitive and Behavioral Neurology. 2016; 29(4):174-189. | Excluded | Based on title and abstract |
| 521 | Koh JL, 2012 | Pediatric epilepsy surgery anesthetic considerations | Anesthesiology Clinics. 2012; 30(2):191-206. | Excluded | Based on title and abstract |
| 522 | Kong H, 2022 | Perioperative neurocognitive disorders: A narrative review focusing on diagnosis, prevention, and treatment | CNS Neurosci Ther. 2022; 28(8):1147-1167. | Excluded | Based on title and abstract |
| 523 | Koning L, 2021 | Sleep medication and sedative use in dutch intensive care units: A nationwide survey | Netherlands Journal of Critical Care. 2021; 29(5):201-206. | Excluded | Based on title and abstract |
| 524 | Korc GB, 2015 | Prevention of post-operative delirium in older patients with cancer undergoing surgery | Journal of Geriatric Oncology. 2015; 6(1):60-69. | Excluded | Based on title and abstract |
| 525 | Kortelainen J, 2018 | Nighttime slow wave activity in recovery from ICU delirium: A pilot study | Intensive Care Medicine Experimental. 2018; 6. | Excluded | Based on title and abstract |
| 526 | Kotecha B, 2018 | Drug induced sleep endoscopy: Its role in evaluation of the upper airway obstruction and patient selection for surgical and nonsurgical treatment | Journal of Thoracic Disease. 2018; 10:S40-S47. | Excluded | Based on title and abstract |
| 527 | Kotfis K, 2018 | ICU delirium a diagnostic and therapeutic challenge in the intensive care unit | Anaesthesiology Intensive Therapy. 2018; 50(2):128-140. | Excluded | Based on title and abstract |
| 528 | Krause C, 2022 | Dexmedetomidine and paralytic exposure after damage control laparotomy: risk factors for delirium? Results from the EAST SLEEP-TIME multicenter trial | Eur J Trauma Emerg Surg. 2022; 48(3):2097-2105. | Excluded | Based on title and abstract |
| 529 | Krishnaiyan R, 2013 | Drugs affecting the autonomic nervous system | Anaesthesia and Intensive Care Medicine. 2013; 14(12):548-553. | Excluded | Based on title and abstract |
| 530 | Kudchadkar SR, 2014 | Sedation, sleep promotion, and delirium screening practices in the care of mechanically ventilated children: A wake-up call for the pediatric critical care community | Critical Care Medicine. 2014; 42(7):1592-1600. | Excluded | Based on title and abstract |
| 531 | Kudchadkar SR, 2014 | Sedation, sleep promotion, and delirium screening practices in the PICU: A wake-up call for the pediatric critical care community | Pediatric Critical Care Medicine. 2014; 15(4):19-20. | Excluded | Based on title and abstract |
| 532 | Kumar A, 2021 | Comeback of ketamine: Resurfacing facts and dispelling myths | Korean Journal of Anesthesiology. 2021; 74(2):103-114. | Excluded | Based on title and abstract |
| 533 | Kumar S, 2022 | A prospective randomised study to evaluate the efficacy of propofol-ketamine (ketofol) and dexmedetomidine for performing drug induced sleep endoscopy (DISE) in patients of obstructive sleep apnea | Sleep medicine. 2022; 100:S236‐S237. | Excluded | Based on title and abstract |
| 534 | Kundra T, 2017 | To evaluate dexmedetomidine as an additive to propofol for sedation for elective cardioversion in a cardiac intensive care unit: A double-blind randomized controlled trial | Annals of Cardiac Anaesthesia. 2017; 20(3):337-340. | Excluded | Based on title and abstract |
| 535 | Kunisawa T, 2005 | Sedation of a pediatric patient using dexmedetomidine for day-care MRI | Japanese Journal of Anesthesiology. 2005; 54(9):1047-1050. | Excluded | Based on title and abstract |
| 536 | Kuragaichi T, 2015 | Usefulness of dexmedetomidine for sedation in patients with end-stage heart failure: A report of two cases | Journal of Cardiac Failure. 2015; 21(10):S203. | Excluded | Based on title and abstract |
| 537 | Kurdi MS, 2013 | The role of melatonin in anaesthesia and critical care | Indian Journal of Anaesthesia. 2013; 57(2):137-144. | Excluded | Based on title and abstract |
| 538 | Kurdi MS, 2018 | Does the phase of the menstrual cycle really matter to anaesthesia? | Indian Journal of Anaesthesia. 2018; 62(5):330-336. | Excluded | Based on title and abstract |
| 539 | Kushikata T, 2012 | Sleep in anesthesiology - What can we learn about anesthesia from studying sleep? | Trends in Anaesthesia and Critical Care. 2012; 2(1):30-35. | Excluded | Based on title and abstract |
| 540 | Kuyrukluyildiz U, 2015 | Comparison of dexmedetomidine and propofol used for drug-induced sleep endoscopy in patients with obstructive sleep apnea syndrome | Int J Clin Exp Med. 2015; 8(4):5691-5698. | Excluded | Based on title and abstract |
| 541 | Kwon WK, 2016 | Microelectrode recording (MER) findings during sleep-awake anesthesia using dexmedetomidine in deep brain stimulation surgery for Parkinson's disease | Clin Neurol Neurosurg. 2016; 143:27-33. | Excluded | Based on title and abstract |
| 542 | Lakshmi KB, 2022 | Effects of adding dexmedetomidine to ropivacaine for paravertebral block in breast cancer surgery | European Journal of Molecular and Clinical Medicine. 2022; 9(5):210-218. | Excluded | Based on title and abstract |
| 543 | Lam DJ, 2019 | Relationship between Drug-Induced Sleep Endoscopy Findings, Tonsil Size, and Polysomnographic Outcomes of Adenotonsillectomy in Children | Otolaryngology - Head and Neck Surgery (United States). 2019; 161(3):507-513. | Excluded | Based on title and abstract |
| 544 | Lamey PS, 2022 | Iatrogenic opioid withdrawal syndromes in adults in intensive care units: a narrative review | Journal of Thoracic Disease. 2022; 14(6):2297-2308. | Excluded | Based on title and abstract |
| 545 | Landsness EC, 2017 | Mapping the neural basis of functional connectivity in genetically-encoded calcium indicator (GECI) mice during wakefulness, sleep, and under anesthesia | Annals of Neurology. 2017; 82:S208-S209. | Excluded | Based on title and abstract |
| 546 | Latmore M, 2012 | Sedation during surgery under regional anesthesia: Which drug is best for my patient? | Regional Anesthesia and Pain Medicine. 2012; 37(5):E77-E79. | Excluded | Based on title and abstract |
| 547 | Lazar SB, 2011 | Systemic administration of dexmedetomidine (DEX) disrupts sleep architecture and microdialysis delivery of dex to rat substantia innominata (SI) does not increase adenosine levels | Sleep. 2011; 34:A7. | Excluded | Based on title and abstract |
| 548 | Lazar SB, 2012 | Dexmedetomidine, adenosine, and sleep: Convergence or conflation? | British Journal of Anaesthesia. 2012; 108(2):348P. | Excluded | Based on title and abstract |
| 549 | LeBlanc JM, 2006 | Role of the bispectral index in sedation monitoring in the ICU | Annals of Pharmacotherapy. 2006; 40(3):490-500. | Excluded | Based on title and abstract |
| 550 | Lee JK, 2019 | Adverse events associated with drugs used for endoscopic sedation: A nationwide assessment | Endoscopy. 2019; 51(4):S189-S190. | Excluded | Based on title and abstract |
| 551 | Lee JM, 2014 | Clinical year in review 2014: Critical care medicine | Tuberculosis and Respiratory Diseases. 2014; 77(1):6-12. | Excluded | Based on title and abstract |
| 552 | Lee S, 2019 | Dexmedetomidine: present and future directions | Korean J Anesthesiol. 2019; 72(4):323-330. | Excluded | Based on title and abstract |
| 553 | Lee SSH, 2015 | Use of the draeger apollo to deliver bilevel positive pressure ventilation during awake frontal craniotomy for a patient with severe chronic obstructive pulmonary disease | A and A Case Reports. 2015; 5(11):202-205. | Excluded | Based on title and abstract |
| 554 | Lee-Archer P, 2018 | Does dexmedetomidine given as a premedication or intraoperatively reduce post-hospitalisation behaviour change in children? A study protocol for a randomised controlled trial in a tertiary paediatric hospital | BMJ Open. 2018; 8(4):e019915. | Excluded | Based on title and abstract |
| 555 | Lee-Archer PF, 2020 | The effect of dexmedetomidine on postoperative behaviour change in children: a randomised controlled trial | Anaesthesia. 2020; 75(11):1461-1468. | Excluded | Based on title and abstract |
| 556 | Lefman SH, 2019 | Psychogenic stress in hospitalized veterinary patients: Causation, implications, and therapies | Journal of Veterinary Emergency and Critical Care. 2019; 29(2):107-120. | Excluded | Based on title and abstract |
| 557 | Lemmens HJM, 2013 | Pharmacology and obesity | International Anesthesiology Clinics. 2013; 51(3):52-66. | Excluded | Based on title and abstract |
| 558 | Lenhardt R, 2003 | Monitoring and thermal management | Best Practice and Research: Clinical Anaesthesiology. 2003; 17(4):569-581. | Excluded | Based on title and abstract |
| 559 | Lerman J, 2009 | A disquisition on sleep-disordered breathing in children | Paediatric Anaesthesia. 2009; 19(SUPPL. 1):100-108. | Excluded | Based on title and abstract |
| 560 | Lerman J, 2013 | Preoperative assessment and premedication in paediatrics | European Journal of Anaesthesiology. 2013; 30(11):645-650. | Excluded | Based on title and abstract |
| 561 | Levanen J, 1995 | Dexmedetomidine premedication attenuates ketamine-induced cardiostimulatory effects and postanesthetic delirium | Anesthesiology. 1995; 82(5):1117-1125. | Excluded | Based on title and abstract |
| 562 | Lewis LD, 2017 | Hierarchy in disruption of large-scale networks across altered arousal states | British Journal of Anaesthesia. 2017; 119(4):566-568. | Excluded | Based on title and abstract |
| 563 | Lewis SR, 2015 | Alpha-2 adrenergic agonists for the prevention of shivering following general anaesthesia | Cochrane Database of Systematic Reviews. 2015(8). | Excluded | Based on title and abstract |
| 564 | Li L, 2019 | A comparison of dezocine and dexmedetomidine with nsaids in burn wound dressing change procedures: A prospective, double-blind, randomized clinical trial | International Journal of Clinical and Experimental Medicine. 2019; 12(12):13651-13658. | Excluded | Based on title and abstract |
| 565 | Li M, 2023 | Effect of dexmedetomidine on postoperative delirium in patients undergoing awake craniotomies: study protocol of a randomized controlled trial | Trials. 2023; 24(1):607. | Excluded | Based on title and abstract |
| 566 | Li N, 2022 | Effects of dexmedetomidine on oxygenation and inflammatory factors in patients undergoing uvulopalatopharyngoplasty: a prospective, randomized, placebo-controlled trial | Sleep Breath. 2023; 27(3):1099-1106. | Excluded | Based on title and abstract |
| 567 | Li N, 2023 | Effects of dexmedetomidine on oxygenation and inflammatory factors in patients undergoing uvulopalatopharyngoplasty: a prospective, randomized, placebo-controlled trial | Sleep Breath. 2023; 27(3):1099-1106. | Excluded | Based on title and abstract |
| 568 | Li P, 2014 | Feasibility study for the dexmetomidine utend the drug induced sleep endoscopy | Lin Chung Er Bi Yan Hou Tou Jing Wai Ke Za Zhi. 2014; 28(15):1151-1154. | Excluded | Based on title and abstract |
| 569 | Li R, 2021 | Clinical risk factors for sleep disruption in critically ill children | Pediatric Critical Care Medicine. 2021; 22(SUPPL 1):69-70. | Excluded | Based on title and abstract |
| 570 | Li SY, 2019 | Comparison of intravenous lidocaine and dexmedetomidine infusion for prevention of postoperative catheter-related bladder discomfort: A randomized controlled trial | BMC Anesthesiology. 2019; 19(1). | Excluded | Based on title and abstract |
| 571 | Li X, 2022 | Application of dexmetomidine in gynecological laparoscopic surgery and its effect on S-100 尾 protein and cognitive function in patients | Minerva Medica. 2022; 113(3):599-601. | Excluded | Based on title and abstract |
| 572 | Li Y, 2016 | Effect of dexmedetomidine combined with oxycodone hydrochloride on post-anesthesia in elderly patients with gastrointestinal surgery | Chinese Journal of New Drugs. 2016; 25(8):903-907. | Excluded | Based on title and abstract |
| 573 | Li Y, 2017 | Dexmedetomidine Effect on Emergence Agitation and Delirium in Children Undergoing Laparoscopic Hernia Repair: a Preliminary Study | Journal of international medical research. 2017; 45(3):973‐983. | Excluded | Based on title and abstract |
| 574 | Li Y, 2019 | Postoperative analgesia with ropivacaine and dexmedetomidine for ultrasound-guided fascia iliaca compartment block after arthroscopic knee surgery | Saudi journal of anaesthesia. 2019; 13(2):100‐105. | Excluded | Based on title and abstract |
| 575 | Li Y, 2022 | Development and validation of prediction models for neurocognitive disorders in adult patients admitted to the ICU with sleep disturbance | CNS Neuroscience and Therapeutics. 2022; 28(4):554-565. | Excluded | Based on title and abstract |
| 576 | Lim YP, 2018 | The comparison between propofol and dexmedetomidine infusion on perioperative anxiety during regional anesthesia | Turkish journal of medical sciences. 2018; 48(6):1219‐1227. | Excluded | Based on title and abstract |
| 577 | Lin D, 2021 | Perioperative sleep disorder: A review | Front Med (Lausanne). 2021; 8:640416. | Excluded | Based on title and abstract |
| 578 | Lin TF, 2009 | Effect of combining dexmedetomidine and morphine for intravenous patient-controlled analgesia | British Journal of Anaesthesia. 2009; 102(1):117-122. | Excluded | Based on title and abstract |
| 579 | Lin TY, 2018 | Comparison of dexmedetomidine to propofol for sedation of endobronchial ultrasound-guided transbronchial needle aspiration: A randomized controlled trial | Respirology. 2018; 23:222. | Excluded | Based on title and abstract |
| 580 | Lin TY, 2019 | Comparison of dexmedetomidine to propofol for sedation of endobronchial ultrasound-guided transbronchial needle aspiration | European Respiratory Journal. 2019; 54. | Excluded | Based on title and abstract |
| 581 | Lin TY, 2020 | Dexmedetomidine sedation for endobronchial ultrasound-guided transbronchial needle aspiration, a randomised controlled trial | ERJ Open Res. 2020; 6(4). | Excluded | Based on title and abstract |
| 582 | Liu B, 2022 | Recent advances and perspectives of postoperative neurological disorders in the elderly surgical patients | CNS Neuroscience and Therapeutics. 2022; 28(4):470-483. | Excluded | Based on title and abstract |
| 583 | Liu J, 2018 | Shivering prevention and treatment during cesarean delivery under neuraxial anesthesia: A systematic review | Minerva Anestesiologica. 2018; 84(12):1393-1405. | Excluded | Based on title and abstract |
| 584 | Liu J, 2019 | Sedation effects of intranasal dexmedetomidine combined with ketamine and risk factors for sedation failure in young children during transthoracic echocardiography | Paediatric Anaesthesia. 2019; 29(1):77-84. | Excluded | Based on title and abstract |
| 585 | Liu J, 2020 | Analysis of optimizing effects of dexmedetomidine and oxycodone on cytokine levels in patients with laparoscopic radical gastrectomy | Indian Journal of Pharmaceutical Sciences. 2020; 82(4):702-706. | Excluded | Based on title and abstract |
| 586 | Liu KA, 2020 | Anesthetic management of children undergoing drug-induced sleep endoscopy: A retrospective review | Int J Pediatr Otorhinolaryngol. 2020; 139:110440. | Excluded | Based on title and abstract |
| 587 | Liu M, 2018 | Gαi and Gβγ subunits have opposing effects on dexmedetomidine-induced sedation | European Journal of Pharmacology. 2018; 831:28-37. | Excluded | Based on title and abstract |
| 588 | Liu M, 2020 | The effects of dexmedetomidine in general anesthesia on the perioperative hemodynamics and postoperative cognitive functions of patients with sleep apnea syndrome in the perioperative period of uvulopalatopharyngoplasty | International journal of clinical and experimental medicine. 2020; 13(3):2086‐2094. | Excluded | Based on title and abstract |
| 589 | Liu S, 2021 | The effectiveness and safety of intravenous dexmedetomidine of different concentrations combined with butorphanol for post-caesarean section analgesia: a randomized controlled trial | Drug design, development and therapy. 2021; 15:689‐698. | Excluded | Based on title and abstract |
| 590 | Liu X, 2021 | Recent advances in the clinical value and potential of dexmedetomidine | J Inflamm Res. 2021; 14:7507-7527. | Excluded | Based on title and abstract |
| 591 | Liu Z, 2022 | Application of inhaled sevoflurane anesthesia without intubation in oral daytime surgery in children | Minerva Pediatrics. 2022; 74(2):229-231. | Excluded | Based on title and abstract |
| 592 | Loh PS, 2016 | Comparing the efficacy and safety between propofol and dexmedetomidine for sedation in claustrophobic adults undergoing magnetic resonance imaging (PADAM trial) | Journal of Clinical Anesthesia. 2016; 34:216-222. | Excluded | Based on title and abstract |
| 593 | Lombo C, 2022 | Pharyngoplasty for obstructive sleep apnea: The influence of surgical technique | Acta Otorrinolaringologica Espanola. 2022; 73(6):362-369. | Excluded | Based on title and abstract |
| 594 | Long E, 2017 | Monitoring and morphologic classification of pediatric cataract using slit-lamp-adapted photography | Translational Vision Science and Technology. 2017; 6(6). | Excluded | Based on title and abstract |
| 595 | Long MHY, 2022 | Sevoflurane requirements during electroencephalogram (EEG)-guided vs standard anesthesia Care in Children: A randomized controlled trial | Journal of Clinical Anesthesia. 2022; 81. | Excluded | Based on title and abstract |
| 596 | Longrois D, 2014 | Sedation in non-invasive ventilation: Do we know what to do (and why)? | Multidisciplinary Respiratory Medicine. 2014; 9(1). | Excluded | Based on title and abstract |
| 597 | Lookabaugh S, 2020 | Drug-induced sleep endoscopy findings in surgically-na茂ve obese vs non-obese children | International Journal of Pediatric Otorhinolaryngology. 2020; 138. | Excluded | Based on title and abstract |
| 598 | Loots H, 2006 | Agents for sedation in ophthalmic surgery: A review of the pharmacodynamics and clinical applications | Current Anaesthesia and Critical Care. 2006; 17(3-4):179-190. | Excluded | Based on title and abstract |
| 599 | Lopez L, 2019 | Usage of psychotropic drug therapy in a polyvalent ICU: A retrospective study | Intensive Care Medicine Experimental. 2019; 7. | Excluded | Based on title and abstract |
| 600 | Losiniecki A, 2010 | Management of traumatic brain injury | Current Treatment Options in Neurology. 2010; 12(2):142-154. | Excluded | Based on title and abstract |
| 601 | Lu Y, 2019 | Promoting sleep and circadian health may prevent postoperative delirium: A systematic review and meta-analysis of randomized clinical trials | Sleep Med Rev. 2019; 48:101207. | Excluded | Based on title and abstract |
| 602 | Luo J, 2017 | Postoperative pain management in the postanesthesia care unit: An update | Journal of Pain Research. 2017; 10:2687-2698. | Excluded | Based on title and abstract |
| 603 | Luo M, 2020 | Sleep disturbances after general anesthesia: current perspectives | Frontiers in Neurology. 2020; 11. | Excluded | Based on title and abstract |
| 604 | Luscri N, 2006 | Monitored anesthesia care with a combination of ketamine and dexmedetomidine during magnetic resonance imaging in three children with trisomy 21 and obstructive sleep apnea | Paediatric Anaesthesia. 2006; 16(7):782-786. | Excluded | Based on title and abstract |
| 605 | Luthra A, 2017 | Alleviating stress response to tracheal extubation in neurosurgical patients: a comparative study of two infusion doses of dexmedetomidine | Journal of neurosciences in rural practice. 2017; 8(5):49‐56. | Excluded | Based on title and abstract |
| 606 | Luz M, 2022 | Practices in sedation, analgesia, mobilization, delirium, and sleep deprivation in adult intensive care units (SAMDS-ICU): an international survey before and during the COVID-19 pandemic | Annals of Intensive Care. 2022; 12(1). | Excluded | Based on title and abstract |
| 607 | Ma K, 2019 | Awake craniotomy in a patient with history of posttraumatic stress disorder: A clinical dilemma | Journal of Neurosurgical Anesthesiology. 2019; 31(4):475. | Excluded | Based on title and abstract |
| 608 | Ma Y, 2021 | Laparoscopic sleeve gastrectomy under general anesthesia in severely obese patients: A single-centered retrospective study | Peer J. 2021; 9. | Excluded | Based on title and abstract |
| 609 | Mahendru V, 2013 | A comparison of intrathecal dexmedetomidine, clonidine, and fentanyl as adjuvants to hyperbaric bupivacaine for lower limb surgery: A double blind controlled study | Journal of Anaesthesiology Clinical Pharmacology. 2013; 29(4):496-502. | Excluded | Based on title and abstract |
| 610 | Mahmoud M, 2010 | Effect of increasing depth of dexmedetomidine anesthesia on upper airway morphology in children | Br J Anaesth. 2015; 115(2):171-182. | Excluded | Based on title and abstract |
| 611 | Mahmoud M, 2015 | Dexmedetomidine: review, update, and future considerations of paediatric perioperative and periprocedural applications and limitations | Paediatric Anaesthesia. 2010; 20(6):506-515. | Excluded | Based on title and abstract |
| 612 | Mahmoud M, 2017 | Upper airway reflexes are preserved during dexmedetomidine sedation in children with down syndrome and obstructive sleep apnea | Journal of Clinical Sleep Medicine. 2017; 13(5):721-727. | Excluded | Based on title and abstract |
| 613 | Makary L, 2010 | Prolonged recovery associated with dexmedetomidine when used as a sole sedative agent in office-based oral and maxillofacial surgery procedures | J Oral Maxillofac Surg. 2010; 68(2):386-391. | Excluded | Based on title and abstract |
| 614 | Maldonado JR, 2013 | Neuropathogenesis of delirium: Review of current etiologic theories and common pathways | American Journal of Geriatric Psychiatry. 2013; 21(12):1190-1222. | Excluded | Based on title and abstract |
| 615 | Mantz J, 2011 | Dexmedetomidine: New insights | European Journal of Anaesthesiology. 2011; 28(1):3-6. | Excluded | Based on title and abstract |
| 616 | Mantz J, 2014 | Delirium in the adult intensive care unit: Does the chosen drug play a significant role? | Reanimation. 2014; 23(SUPPL.2):S372-S378. | Excluded | Based on title and abstract |
| 617 | Mart MF, 2021 | Prevention and management of delirium in the intensive care unit | Seminars in Respiratory and Critical Care Medicine. 2021; 42(1):112-126. | Excluded | Based on title and abstract |
| 618 | Marty P, 2021 | Obturator nerve block does not provide analgesic benefits in total hip arthroplasty under multimodal analgesic regimen: A randomized controlled trial | Regional Anesthesia and Pain Medicine. 2021; 46(8):657-662. | Excluded | Based on title and abstract |
| 619 | Mashour GA, 2011 | Consciousness versus responsiveness: Insights from general anesthetics | Brain and Cognition. 2011; 77(3):325-326. | Excluded | Based on title and abstract |
| 620 | Mason K, 2012 | Intramuscular dexmedetomidine for pediatric electroencephalogram (EEG) sedation | European Journal of Anaesthesiology. 2012; 29:161. | Excluded | Based on title and abstract |
| 621 | Mason KP, 2008 | The pediatric sedation service: Who is appropriate to sedate, which medications should I use, who should prescribe the drugs, how do I bill? | Pediatric Radiology. 2008; 38(SUPPL. 2):S218-S224. | Excluded | Based on title and abstract |
| 622 | Mason KP, 2009 | Effects of dexmedetomidine sedation on the EEG in children | Paediatr Anaesth. 2009; 19(12):1175-1183. | Excluded | Based on title and abstract |
| 623 | Mason KP, 2013 | Dexmedetomidine offers an option for safe and effective sedation for nuclear medicine imaging in children | Radiology. 2013; 267(3):911-917. | Excluded | Based on title and abstract |
| 624 | Mason KP, 2014 | Hemodynamic response to fluid management in children undergoing dexmedetomidine sedation for MRI | American Journal of Roentgenology. 2014; 202(6):W574-W579. | Excluded | Based on title and abstract |
| 625 | Mason KP, 2015 | Response to Dr. Phelps et al.: high-dose dexmedetomidine for noninvasive pediatric procedural sedation and discharge readiness | Paediatric Anaesthesia. 2015; 25(9):963. | Excluded | Based on title and abstract |
| 626 | Massimiliano L, 2023 | Continuous erector spinae and serratus-intercostal block with ketamine-dexmedetomidine sedation for quadrantectomy and axillary dissection in a multimorbid patient | Report Number. 2023:2168-8184 | Excluded | Based on title and abstract |
| 627 | Mathews AMV, 2013 | A case report on the anaesthetic management of dexmedetomidineinduced sleep endoscopy and transoral robotic surgery for the treatment of obstructive sleep apnoea | Proceedings of Singapore Healthcare. 2013; 22(2):151-155. | Excluded | Based on title and abstract |
| 628 | Matsunami S, 2014 | Awake insertion of i-gel under dexmedetomidine sedation in a patient with severe obstructive sleep apnea syndrome | Masui. 2014; 63(7):807-809. | Excluded | Based on title and abstract |
| 629 | Matsunami S, 2014 | Awake insertion of i-gel under dexmedetomidine sedation in a patient with severe obstructive sleep apnea syndrome and angina | Japanese Journal of Anesthesiology. 2014; 63(7):807-809. | Excluded | Based on title and abstract |
| 630 | Mavropoulos G, 2014 | Alpha-2 adrenoreceptor agonists in anaesthesia and intensive care medicine | Rev Med Liege. 2014; 69(2):97-101. | Excluded | Based on title and abstract |
| 631 | Mavropoulos G, 2014 | Interest for alpha-2 adrenoreceptor agonists in anaesthesia and intensive care medicine | Revue Medicale de Liege. 2014; 69(2):97-101. | Excluded | Based on title and abstract |
| 632 | Maze M, 1995 | Anesthetic and analgesic actions of α2 adrenoceptor agonists | Pharmacology Communications. 1995; 6(1-3):175-182. | Excluded | Based on title and abstract |
| 633 | Maze M, 2004 | Dexmedetomidine and opioid interactions: Defining the role of dexmedetomidine for intensive care unit sedation | Anesthesiology. 2004; 101(5):1059-1061. | Excluded | Based on title and abstract |
| 634 | McConnell K, 2012 | Effect of dexmedetomidine on upper airway collapsibility in children with down's syndrome | American Journal of Respiratory and Critical Care Medicine. 2012; 185. | Excluded | Based on title and abstract |
| 635 | McEvoy MD, 2022 | Opioid-sparing perioperative analgesia within enhanced recovery programs | Anesthesiology Clinics. 2022; 40(1):35-58. | Excluded | Based on title and abstract |
| 636 | McGinlay M, 2016 | An opioid-free "wake throughout" craniotomy using dexmedetomidine and nasal high flow oxygen in an obese man with suspected obstructive sleep apnoea (OSA) | Journal of Neurosurgical Anesthesiology. 2016; 28(2):S17. | Excluded | Based on title and abstract |
| 637 | Meerts C, 2013 | Anxiolytics, sedatives and hypnotics | Anaesthesia and Intensive Care Medicine. 2013; 14(8):355-360. | Excluded | Based on title and abstract |
| 638 | Melis Y, 2023 | Dexmedetomidine, an alpha 2A receptor agonist, triggers seizures unilaterally in GAERS during the pre-epileptic phase: does the onset of spike-and-wave discharges occur in a focal manner? | Front Neurol. 2023; 14:1231736. | Excluded | Based on title and abstract |
| 639 | Melo WAL, 2021 | Apnea free sedation for curettage in morbidly obese patient: Is ketodex a viable option? | Anesthesia and Analgesia. 2021; 133(3 SUPPL 2):1809. | Excluded | Based on title and abstract |
| 640 | Memary E, 2017 | Case report: Successful regional anesthesia for awake fiber optic intubation in a rare case of grisel's syndrome | Regional Anesthesia and Pain Medicine. 2017; 42(5):e109. | Excluded | Based on title and abstract |
| 641 | Metzler L, 2022 | Cheer (collaborating to help everyone effectively recover): Early mobility in a tertiary PICU | Critical Care Medicine. 2022; 50(1 SUPPL):657. | Excluded | Based on title and abstract |
| 642 | Mine T, 2014 | A case report: perioperative management of adenotonsillectomy in a morbidly obese patient with severe obstructive sleep apnea syndrome | Masui. 2014; 63(11):1284-1286. | Excluded | Based on title and abstract |
| 643 | Minguez JJ, 2021 | Benign juvenile idiopathic epilepsy in captive Iberian lynx (Lynx pardinus) in the ex situ conservation program (2005-2019) | BMC Veterinary Research. 2021; 17(1). | Excluded | Based on title and abstract |
| 644 | Miranda HG, 2017 | Nocturnal dexmedetomidine in nonintubated, critically ill patients: Sleep or sedation? | Anesthesiology. 2017; 127(2):397-398. | Excluded | Based on title and abstract |
| 645 | Mirski MA, 2007 | Critical care sedation for neuroscience patients | Journal of the Neurological Sciences. 2007; 261(1-2):16-34. | Excluded | Based on title and abstract |
| 646 | Mirski MA, 2008 | Sedation and pain management in acute neurological disease | Seminars in Neurology. 2008; 28(5):611-630. | Excluded | Based on title and abstract |
| 647 | Mirski MA, 2010 | Sedation and analgesia in acute neurologic disease | Current Opinion in Critical Care. 2010; 16(2):81-91. | Excluded | Based on title and abstract |
| 648 | Mistraletti G, 2008 | Sleep and delirium in the intensive care unit | Minerva Anestesiologica. 2008; 74(6):329-333. | Excluded | Based on title and abstract |
| 649 | Mizuno T, 2022 | Effects of general anesthesia on behavioral circadian rhythms and clock-gene expression in the suprachiasmatic nucleus in rats | Histochem Cell Biol. 2022; 158(2):149-158. | Excluded | Based on title and abstract |
| 650 | Mo Y, 2013 | Role of dexmedetomidine for the prevention and treatment of delirium in intensive care unit patients | Annals of Pharmacotherapy. 2013; 47(6):869-876. | Excluded | Based on title and abstract |
| 651 | Mo Y, 2017 | Effects of dexmedetomidine in reducing post-cesarean adverse reactions | Experimental and therapeutic medicine. 2017; 14(3):2036‐2039. | Excluded | Based on title and abstract |
| 652 | Modir H, 2018 | Comparing remifentanil, magnesium sulfate, and dexmedetomidine for intraoperative hypotension and bleeding and postoperative recovery in endoscopic sinus surgery and tympanomastoidectomy | Medical Gas Research. 2018; 8(2):42-47. | Excluded | Based on title and abstract |
| 653 | Mohamed SAB, 2016 | Intrathecal dexmedetomidine, ketamine, and their combination added to bupivacaine for postoperative analgesia in major abdominal cancer surgery | Pain Physician. 2016; 19(6):E829-E839. | Excluded | Based on title and abstract |
| 654 | Molto L, 2016 | Regional anesthesia and sedation technique with dexmedetomidine for awake craniotomy | Journal of Neurosurgical Anesthesiology. 2016; 28(2):S16. | Excluded | Based on title and abstract |
| 655 | Mongodi S, 2021 | High prevalence of acute stress disorder and persisting symptoms in ICU survivors after COVID-19 | Intensive Care Medicine. 2021; 47(5):616-618. | Excluded | Based on title and abstract |
| 656 | Moody OA, 2021 | The neural circuits underlying general anesthesia and sleep | Anesth Analg. 2021; 132(5):1254-1264. | Excluded | Based on title and abstract |
| 657 | Mooney KL, 2022 | The quest for a DISE protocol | Otolaryngology - Head and Neck Surgery (United States). 2022; 167(3):590-599. | Excluded | Based on title and abstract |
| 658 | Morandi A, 2009 | Advances in sedation for critically ill patients | Minerva Anestesiologica. 2009; 75(6):385-391. | Excluded | Based on title and abstract |
| 659 | Morandi A, 2009 | Delirium in the intensive care unit | International Review of Psychiatry. 2009; 21(1):43-58. | Excluded | Based on title and abstract |
| 660 | Morrison C, 2019 | Diagnosis and perioperative management in pediatric sleep-disordered breathing | Paediatric Anaesthesia. 2019; 29(3):291-292. | Excluded | Based on title and abstract |
| 661 | Moskowitz A, 2020 | Identification, collection, and reporting of harms among non-industry-sponsored randomized clinical trials of pharmacologic interventions in the critically ill population: a systematic review | Critical Care. 2020; 24(1). | Excluded | Based on title and abstract |
| 662 | Motshabi-Chakane P, 2021 | Contemporary neuroprotection strategies during cardiac surgery: State of the art review | International Journal of Environmental Research and Public Health. 2021; 18(23). | Excluded | Based on title and abstract |
| 663 | Mu B, 2023 | Determination of the effective dose of dexmedetomidine to achieve loss of consciousness during anesthesia induction | Front Med (Lausanne). 2023; 10:1158085. | Excluded | Based on title and abstract |
| 664 | Mulier JP, 2019 | Anaesthetic factors affecting outcome after bariatric surgery, a retrospective levelled regression analysis | Obesity Surgery. 2019; 29(6):1841-1850. | Excluded | Based on title and abstract |
| 665 | Muller S, 2008 | Clinical efficacy of dexmedetomidine alone is less than propofol for conscious sedation during ERCP | Gastrointestinal Endoscopy. 2008; 67(4):651-659. | Excluded | Based on title and abstract |
| 666 | Mun-Price C, 2022 | The effect of anesthesia without opioid on perioperative opioid demand in children with severe obstructive sleep apnea (OSA) for adenotonsillectomies 鈥 single-center retrospective observational study | JA Clinical Reports. 2022; 8(1). | Excluded | Based on title and abstract |
| 667 | Mupamombe CT, 2019 | Dexmedetomidine as an Option for Opioid Refractory Pain in the Hospice Setting | Journal of Palliative Medicine. 2019; 22(11):1478-1481. | Excluded | Based on title and abstract |
| 668 | Murabito P, 2019 | Comparison of genioglossus muscle activity and efficiency of dexmedetomidine or propofol during drug-induced sleep endoscopy in patients with obstructive sleep apnea/hypopnea syndrome | Eur Rev Med Pharmacol Sci. 2019; 23(1):389-396. | Excluded | Based on title and abstract |
| 669 | Murcia D, 2022 | Investigation of asleep versus awake motor mapping in resective brain surgery | World Neurosurgery. 2022; 157:e129-e136. | Excluded | Based on title and abstract |
| 670 | Musica A, 2018 | Effects of propofol and dexmedetomidine on sleep architecture in ICU patients | Critical Care. 2018; 22. | Excluded | Based on title and abstract |
| 671 | Mylavarapu G, 2017 | Effect of a combination of dexmedetomidine and ketamine anesthesia on the upper airway caliber in normal children | American Journal of Respiratory and Critical Care Medicine. 2017; 195. | Excluded | Based on title and abstract |
| 672 | Mylavarapu G, 2017 | Predicting critical closing airway pressure in obstructive sleep apnea patients using fluid structure interaction simulations | American Journal of Respiratory and Critical Care Medicine. 2017; 195. | Excluded | Based on title and abstract |
| 673 | Nair A, 2017 | Less explored off-label indications of dexmedetomidine | Korean Journal of Anesthesiology. 2017; 70(3):361-362. | Excluded | Based on title and abstract |
| 674 | Nam S, 2015 | Comparison of the sleep pattern by dexmedetomidine and propofol used for sedation: watch-PAT200 analysis | Regional anesthesia and pain medicine. 2015; 40(5 SUPPL. 1):e100. | Excluded | Based on title and abstract |
| 675 | Narayanasamy S, 2021 | Outcomes of dexmedetomidine sedation for drug-induced sleep cine magnetic resonance imaging studies in pediatric obstructive sleep apnea patients | Paediatric Anaesthesia. 2021; 31(11):1241-1249. | Excluded | Based on title and abstract |
| 676 | Naruse S, 2012 | Evaluation of dexmedetomidine hydrochloride as a basal agent in conservative therapy for acute aortic dissection | Intensive Care Medicine. 2012; 38:S159. | Excluded | Based on title and abstract |
| 677 | Nazemi AK, 2017 | Prevention and management of postoperative delirium in elderly patients following elective spinal surgery | Clinical Spine Surgery. 2017; 30(3):112-119. | Excluded | Based on title and abstract |
| 678 | Nct, 2007 | Cognitive protection - dexmedetomidine and cognitive reserve | https://clinicaltrials.gov/show/NCT00455143. 2007. | Excluded | Based on title and abstract |
| 679 | Nct, 2007 | Perioperative cognitive function - dexmedetomidine and cognitive reserve | https://clinicaltrials.gov/show/NCT00561678. 2007. | Excluded | Based on title and abstract |
| 680 | Nct, 2009 | Analgesic effect of peripheral dexmedetomidine | https://clinicaltrials.gov/show/NCT00971178. 2009. | Excluded | Based on title and abstract |
| 681 | Nct, 2009 | Dexmedetomidine versus propofol in vitreoretinal surgery | https://clinicaltrials.gov/show/NCT01001429. 2009. | Excluded | Based on title and abstract |
| 682 | Nct, 2011 | Dexmedetomidine and propofol in children with history of obstructive sleep apnea | https://clinicaltrials.gov/show/NCT01344759. 2011. | Excluded | Based on title and abstract |
| 683 | Nct, 2012 | Comparison of dexmedetomidine and propofol-remifentanil conscious sedation for awake craniotomy for tumor surgery | https://clinicaltrials.gov/show/NCT01545297. 2012. | Excluded | Based on title and abstract |
| 684 | Nct, 2013 | A comparison of surgical outcome following drug-induced sleep endoscopic diagnosis using propofol or dexmedetomidine for sedation : a prospective randomized trial | https://clinicaltrials.gov/show/NCT01895348. 2013. | Excluded | Based on title and abstract |
| 685 | Nct, 2013 | Effect of intravenous dexmedetomidine on analgesic duration of infraclavicular block | https://clinicaltrials.gov/show/NCT01981369. 2013. | Excluded | Based on title and abstract |
| 686 | Nct, 2013 | Postoperative recovery in elderly patients undergoing hip hemi-arthroplasty | https://clinicaltrials.gov/show/NCT01934049. 2013. | Excluded | Based on title and abstract |
| 687 | Nct, 2013 | SLEEP 2013-27th Annual Meeting of the Associated Professional Sleep Societies, LLC - Scientific Highlights/Abstracts of Original Investigations | Sleep. 2013; 36. | Excluded | Based on title and abstract |
| 688 | Nct, 2014 | Dexmedetomidine and delirium in patients after cardiac surgery | https://clinicaltrials.gov/show/NCT02267538. 2014. | Excluded | Based on title and abstract |
| 689 | Nct, 2014 | Dexmedetomidine as an adjuvant for sub-tenon's anesthesia | https://clinicaltrials.gov/show/NCT02327156. 2014. | Excluded | Based on title and abstract |
| 690 | Nct, 2014 | Establishment of ideal IV sedative regimen for successful fiberoptic bronchoscopy in assessing the site, severity of sleep apnea syndrome | https://clinicaltrials.gov/show/NCT02148432. 2014. | Excluded | Based on title and abstract |
| 691 | Nct, 2014 | Evaluation of motor sparing knee block to infiltration analgesia for pain following knee arthroplasty | https://clinicaltrials.gov/show/NCT02540070. 2014. | Excluded | Based on title and abstract |
| 692 | Nct, 2015 | Evaluation of dexmedetomidine on post-operative narcotic requirements and pain scores in bariatric patients | https://clinicaltrials.gov/show/NCT02604940. 2015. | Excluded | Based on title and abstract |
| 693 | Nct, 2015 | Ropivacaine with clonidine for pediatric rectus sheath blocks-the magic combination | https://clinicaltrials.gov/show/NCT02439281. 2015. | Excluded | Based on title and abstract |
| 694 | Nct, 2016 | Dexmedetomidine to treat bariatric surgery-associated pain | https://clinicaltrials.gov/show/NCT02880540. 2016. | Excluded | Based on title and abstract |
| 695 | Nct, 2016 | Influence of dexmedetomidine or propofol on ICU delirium | https://clinicaltrials.gov/show/NCT02807467. 2016. | Excluded | Based on title and abstract |
| 696 | Nct, 2016 | Minimizing ICU neurological dysfunction with dexmedetomidine-induced sleep | https://clinicaltrials.gov/show/NCT02856594. 2016. | Excluded | Based on title and abstract |
| 697 | Nct, 2017 | Dexmedetomidine and long-term outcomes in elderly patients after cardiac surgery | https://clinicaltrials.gov/show/NCT03289325. 2017. | Excluded | Based on title and abstract |
| 698 | Nct, 2017 | Dexmedetomidine supplemented analgesia and 2-year survival after cancer surgery | https://clinicaltrials.gov/show/NCT03012971. 2017. | Excluded | Based on title and abstract |
| 699 | Nct, 2017 | Impact of dexamethasone on the duration of sensory and motor block following spinal anesthesia | https://clinicaltrials.gov/show/NCT03078062. 2017. | Excluded | Based on title and abstract |
| 700 | Nct, 2017 | Investigation of sleep in the intensive care unit | https://clinicaltrials.gov/show/NCT03355053. 2017. | Excluded | Based on title and abstract |
| 701 | Nct, 2017 | Low-dose dexmedetomidine in mechanically ventilated ICU patients | https://clinicaltrials.gov/show/NCT03172897. 2017. | Excluded | Based on title and abstract |
| 702 | Nct, 2018 | Dex as analgesic adjuvant in OSA patients | https://clinicaltrials.gov/show/NCT03613558. 2018. | Excluded | Based on title and abstract |
| 703 | Nct, 2018 | Dexmedetomidine combined with ropivacaine for postoperative continuous femoral nerve block | https://clinicaltrials.gov/show/NCT03629483. 2018. | Excluded | Based on title and abstract |
| 704 | Nct, 2018 | Dexmedetomidine supplemented intravenous analgesia in elderly after orthopedic surgery | https://clinicaltrials.gov/show/NCT03629262. 2018. | Excluded | Based on title and abstract |
| 705 | Nct, 2018 | Propofol and dexmedetomidine on inflammation | https://clinicaltrials.gov/show/NCT03600727. 2018. | Excluded | Based on title and abstract |
| 706 | Nct, 2018 | the influence of sleep improvement through different methods on patients in ICU | https://clinicaltrials.gov/show/NCT03480789. 2018. | Excluded | Based on title and abstract |
| 707 | Nct, 2019 | Dexmedetomidine and outcomes of elderly admitted to ICU after surgery | https://clinicaltrials.gov/show/NCT04204798. 2019. | Excluded | Based on title and abstract |
| 708 | Nct, 2019 | The effect of opioid free anesthesia in bariatric surgeries | https://clinicaltrials.gov/show/NCT04048200. 2019. | Excluded | Based on title and abstract |
| 709 | Nct, 2020 | A comparative study of oral gabapentin, oral alprazolam and intravenous dexmedetomidine on perioperative anxiety and pain during posterior segment eye surgery under peribulbar block: a randomized, double-blind study | https://clinicaltrials.gov/show/NCT04658732. 2020. | Excluded | Based on title and abstract |
| 710 | Nct, 2020 | Application of dexmedetomidine hydrochloride injection in anesthesia for patients without tracheal intubation | https://clinicaltrials.gov/show/NCT04652427. 2020. | Excluded | Based on title and abstract |
| 711 | Nct, 2020 | Co-administration of dexmedetomidine in carotid endarterectomy (CEA) | https://clinicaltrials.gov/show/NCT04662177. 2020. | Excluded | Based on title and abstract |
| 712 | Nct, 2020 | Comparison of caudal block versus dexmedetomidine infusion in pediatric patients undergoing hypospadias repair surgery | https://clinicaltrials.gov/show/NCT04331418. 2020. | Excluded | Based on title and abstract |
| 713 | Nct, 2020 | Dexmedetomidine adjuvant treatment for depressed patients undergoing ECT | https://clinicaltrials.gov/show/NCT04661475. 2020. | Excluded | Based on title and abstract |
| 714 | Nct, 2020 | Dexmedetomidine as an adjuvant for femoral nerve block and functional recovery after total knee arthroplasty | https://clinicaltrials.gov/show/NCT04642651. 2020. | Excluded | Based on title and abstract |
| 715 | Nct, 2020 | Dexmedetomidine on tourniquet induced-systemic effects | https://clinicaltrials.gov/show/NCT04307290. 2020. | Excluded | Based on title and abstract |
| 716 | Nct, 2020 | Dexmedetomidine supplemented analgesia in patients at high-risk of obstructive sleep apnea | https://clinicaltrials.gov/show/NCT04608331. 2020. | Excluded | Based on title and abstract |
| 717 | Nct, 2020 | Effect of intratracheal dexmedetomidine administration on recovery from general anesthesia in pediatrics undergoing lower abdominal surgery | https://clinicaltrials.gov/show/NCT04385602. 2020. | Excluded | Based on title and abstract |
| 718 | Nct, 2020 | Intraperitoneal versus intravenous dexmedetomidine for post-operative analgesia following laparoscopic sleeve gastrectomy surgery | https://clinicaltrials.gov/show/NCT04370392. 2020. | Excluded | Based on title and abstract |
| 719 | Nct, 2020 | Ketamine infusion vs dexmedetomidine infusion in obese patients undergoing bariatric surgery | https://clinicaltrials.gov/show/NCT04576975. 2020. | Excluded | Based on title and abstract |
| 720 | Nct, 2020 | Liposomal bupivacaine vs standard penile block for hypospadias repair | https://clinicaltrials.gov/show/NCT04479371. 2020. | Excluded | Based on title and abstract |
| 721 | Nct, 2020 | Opioid-free anesthetic for tonsillectomy | https://clinicaltrials.gov/show/NCT04528173. 2020. | Excluded | Based on title and abstract |
| 722 | Nct, 2020 | Subtenon and intravenous dexmedetomidine effect on patients undergoing cataract surgery | https://clinicaltrials.gov/show/NCT04668456. 2020. | Excluded | Based on title and abstract |
| 723 | Nct, 2020 | The effect of guanfacine on delirium in critically ill patients | https://clinicaltrials.gov/show/NCT04578886. 2020. | Excluded | Based on title and abstract |
| 724 | Nct, 2021 | Benefits of opioid free anesthesia on morphine consumption in gastric bypass | https://clinicaltrials.gov/show/NCT05004519. 2021. | Excluded | Based on title and abstract |
| 725 | Nct, 2021 | Comparison of doses of dexmedetomidine with bupivacaine in caudal block for duration of analgesia in paediatric infraumblical surgeries | https://clinicaltrials.gov/show/NCT04700228. 2021. | Excluded | Based on title and abstract |
| 726 | Nct, 2021 | Dexmedetomidine supplemented analgesia and delirium after hip fracture surgery | https://clinicaltrials.gov/show/NCT04955249. 2021. | Excluded | Based on title and abstract |
| 727 | Nct, 2021 | Effect of pretreatment of lignocaine versus midazolam in prevention of etomidate induced myoclonus | https://clinicaltrials.gov/show/NCT04921046. 2021. | Excluded | Based on title and abstract |
| 728 | Nct, 2021 | Ketorolac on postoperative pain reduction in pediatric patients with adenotonsillectomy | https://clinicaltrials.gov/show/NCT05074056. 2021. | Excluded | Based on title and abstract |
| 729 | Nct, 2021 | Low-dose s-ketamine and dexmedetomidine in combination with opioids for postoperative analgesia | https://clinicaltrials.gov/show/NCT04791059. 2021. | Excluded | Based on title and abstract |
| 730 | Nct, 2021 | Safety and efficacy of esmketamine versus dexmedetomidine | https://clinicaltrials.gov/show/NCT04877639. 2021. | Excluded | Based on title and abstract |
| 731 | Nct, 2021 | Sedation complications in urology during spinal anesthesia with dexmedetomidine or midazolam regarding OSA risk | https://clinicaltrials.gov/show/NCT04817033. 2021. | Excluded | Based on title and abstract |
| 732 | Nct, 2021 | The neuroprotective effect of dexmedetomidine preserving brain functional connectivity in elderly patients | https://clinicaltrials.gov/show/NCT04973124. 2021. | Excluded | Based on title and abstract |
| 733 | Nct, 2021 | Ultrasound guided rectus sheath block in pediatric single port laparoscopic appendectomy | https://clinicaltrials.gov/show/NCT04838379. 2021. | Excluded | Based on title and abstract |
| 734 | Nct, 2022 | Combination of nalbuphine and dexmedetomidine versus sufentanil and dexmedetomidine on patients | https://clinicaltrials.gov/show/NCT05445024. 2022. | Excluded | Based on title and abstract |
| 735 | Nct, 2022 | Dexmedetomidine versus dexamethasone as adjuncts in erector spinae plane block in modified radical mastectomy | https://clinicaltrials.gov/show/NCT05447949. 2022. | Excluded | Based on title and abstract |
| 736 | Nct, 2022 | Dexmedetomidine versus propofol sedation for drug-induced sleep endoscopy in pediatric obstructive sleep apnea | https://clinicaltrials.gov/show/NCT05303987. 2022. | Excluded | Based on title and abstract |
| 737 | Nct, 2022 | Hemodynamic effect of dexmedetomidine alone with dexmedetomidine plus ketamine combination in CABG | https://clinicaltrials.gov/show/NCT05218161. 2022. | Excluded | Based on title and abstract |
| 738 | Nct, 2022 | The effects of subanesthetic s-ketamine on postoperative delirium and cognitive function in the elderly undergoing non-cardiac thoracic surgery | https://clinicaltrials.gov/show/NCT05242692. 2022. | Excluded | Based on title and abstract |
| 739 | Nct, 2022 | Trazodone vs. quetiapine for the treatment of ICU delirium: a prospective observational pilot study | https://clinicaltrials.gov/show/NCT05307003. 2022. | Excluded | Based on title and abstract |
| 740 | Nelson LE, 2003 | The α2-adrenoceptor agonist dexmedetomidine converges on an endogenous sleep-promoting pathway to exert its sedative effects | Anesthesiology. 2003; 98(2):428-436. | Excluded | Based on title and abstract |
| 741 | Neogi M, 2010 | A comparative study between clonidine and dexmedetomidine used as adjuncts to ropivacaine for caudal analgesia in paediatric patients | Journal of anaesthesiology, clinical pharmacology. 2010; 26(2):149‐153. | Excluded | Based on title and abstract |
| 742 | Newcomer J, 2017 | Psychotomimetic effects of single-dose ketamine in children: Exploring effects of age and alpha-2 agonist pretreatment | Neuropsychopharmacology. 2017; 43:S398-S399. | Excluded | Based on title and abstract |
| 743 | Newsom CT, 2014 | The effectiveness of dexmedetomidine on respiratory adverse events, overall use of narcotics, and pain levels in perioperative patients with obstructive sleep apnea: A systematic review protocol | JBI Database of Systematic Reviews and Implementation Reports. 2014; 12(7):24-34. | Excluded | Based on title and abstract |
| 744 | Nguyen V, 2017 | Alpha-2 agonists | Anesthesiology Clinics. 2017; 35(2):233-245. | Excluded | Based on title and abstract |
| 745 | Nie Y, 2014 | Effect of dexmedetomidine combined with sufentanil for post-caesarean section intravenous analgesia: a randomised, placebo-controlled study | European journal of anaesthesiology. 2014; 31(4):197‐203. | Excluded | Based on title and abstract |
| 746 | Niu JY, 2023 | Effect of different administration routes of dexmedetomidine on postoperative delirium in elderly patients undergoing elective spinal surgery: A prospective randomized double-blinded controlled trial | Anesth Analg. 2023; 136(6):1075-1083. | Excluded | Based on title and abstract |
| 747 | Niu Z, 2021 | Effect of total intravenous anesthesia or inhalation anesthesia on postoperative quality of recovery in patients undergoing total laparoscopic hysterectomy: A randomized controlled trial | Journal of Clinical Anesthesia. 2021; 73. | Excluded | Based on title and abstract |
| 748 | Niwa Y, 2013 | Use of RRa sensor in a pediatric patient with post-adenotonsillectomy | Masui. 2013; 62(7):855-858. | Excluded | Based on title and abstract |
| 749 | Niwa Y, 2013 | Use of RRaR sensor in a pediatric patient with post-adenotonsillectomy | Japanese Journal of Anesthesiology. 2013; 62(7):855-858. | Excluded | Based on title and abstract |
| 750 | Nosanov LB, 2020 | Pain management in burn patients | Current Trauma Reports. 2020; 6(4):161-173. | Excluded | Based on title and abstract |
| 751 | Nunes SL, 2016 | Effect of dexmedetomidine on weaning from mechanical ventilation in intensive care patients | Intensive Care Medicine Experimental. 2016; 4. | Excluded | Based on title and abstract |
| 752 | Ogonowska-Kobusiewicz M, 2008 | Perioperative risk in patients with sleep apnoea | Anestezjol Intens Ter. 2008; 40(3):182-187. | Excluded | Based on title and abstract |
| 753 | Oh MS, 2017 | Current techniques and role of drug-induced sleep endoscopy for obstructive sleep apnea | Current Sleep Medicine Reports. 2017; 3(3):152-163. | Excluded | Based on title and abstract |
| 754 | Oh ST, 2019 | Postoperative delirium | Korean Journal of Anesthesiology. 2019; 72(1):4-12. | Excluded | Based on title and abstract |
| 755 | Ohtsuka M, 2012 | Dexmedetomidine for postoperative sedation in elderly patients with cognitive impairment | Masui. 2012; 61(4):379-383. | Excluded | Based on title and abstract |
| 756 | Ojha S, 2022 | Sedation and analgesia from prolonged pain and stress during mechanical ventilation in preterm infants: is dexmedetomidine an alternative to current practice? | BMJ Paediatr Open. 2022; 6(1). | Excluded | Based on title and abstract |
| 757 | Ok YM, 2016 | Nefopam reduces dysesthesia after percutaneous endoscopic lumbar discectomy | Korean Journal of Pain. 2016; 29(1):40-47. | Excluded | Based on title and abstract |
| 758 | Okamoto T, 2012 | Dexmedetomidine is an excellent sedative for voice monitoring surgery | Masui. 2012; 61(5):542-545. | Excluded | Based on title and abstract |
| 759 | Okutani H, 2020 | Painful myokymia after surgery in a patient with Isaacs' syndrome: a case report | JA Clinical Reports. 2020; 6(1). | Excluded | Based on title and abstract |
| 760 | Olausson A, 2022 | Total opioid-free general anaesthesia can improve postoperative outcomes after surgery, without evidence of adverse effects on patient safety and pain management: A systematic review and meta-analysis | Acta Anaesthesiologica Scandinavica. 2022; 66(2):170-185. | Excluded | Based on title and abstract |
| 761 | Oldham M, 2015 | Sedation in critically ill patients | Critical Care Clinics. 2015; 31(3):563-587. | Excluded | Based on title and abstract |
| 762 | O'Neill A, 2022 | Multimodal analgesia | Anesthesiology Clinics. 2022; 40(3):455-468. | Excluded | Based on title and abstract |
| 763 | Onodera Y, 2017 | A high flow nasal cannula during sleep reduces inspiratory effort but does not reduce respiratory rate | Intensive Care Medicine Experimental. 2017; 5(2). | Excluded | Based on title and abstract |
| 764 | Oriol-Lopez SA, 2011 | What adjuvant for sedation, dexmedetomidine or fentanyl epidural? For abdominal surgery and lower limb | Revista mexicana de anestesiologia. 2011; 34(3):189‐195. | Excluded | Based on title and abstract |
| 765 | Ortiz D, 2020 | Assessment and management of agitation, sleep, and mental illness in the surgical ICU | Current Opinion in Critical Care. 2020; 26(6):634-639. | Excluded | Based on title and abstract |
| 766 | Osman M, 2018 | Effect of dexmedetomidine on mortality and length of icu stay in patients with sepsis requiring mechanical ventilation: A systematic review and meta-analysis of randomized clinical trials | American Journal of Respiratory and Critical Care Medicine. 2018; 197(MeetingAbstracts). | Excluded | Based on title and abstract |
| 767 | Oto J, 2012 | Sleep quality of mechanically ventilated patients sedated with dexmedetomidine | Intensive Care Medicine. 2012; 38(12):1982-1989. | Excluded | Based on title and abstract |
| 768 | Owais Q, 2023 | Recent advances in predicting, preventing, and managing postoperative delirium | Fac Rev. 2023; 12:19. | Excluded | Based on title and abstract |
| 769 | Owens RL, 2016 | Better sleep in the intensive care unit: blue pill or red pill or no pill? | Anesthesiology. 2016; 125(5):835-837. | Excluded | Based on title and abstract |
| 770 | Pactr, 2019 | Opioid sparing anesthesia for bariatric surgery | https://trialsearch.who.int/Trial2.aspx?TrialID=PACTR201911712319290. 2019. | Excluded | Based on title and abstract |
| 771 | Pactr, 2022 | The effect of nebulized dexmedetomidine as sedative premedication in pediatrics undergoing cochlear implantation | https://trialsearch.who.int/Trial2.aspx?TrialID=PACTR202204644214951. 2022. | Excluded | Based on title and abstract |
| 772 | Padiyara TV, 2020 | Dexmedetomidine versus propofol at different sedation depths during drug-induced sleep endoscopy: A randomized trial | Laryngoscope. 2020; 130(1):257-262. | Excluded | Based on title and abstract |
| 773 | Page V, 2021 | Sedation in the intensive care unit | Current Anesthesiology Reports. 2021; 11(2):92-100. | Excluded | Based on title and abstract |
| 774 | Palacios-Cena D, 2016 | How do doctors and nurses manage delirium in intensive care units? A qualitative study using focus groups | BMJ Open. 2016; 6(1). | Excluded | Based on title and abstract |
| 775 | Pandharipande PP, 2017 | The intensive care delirium research agenda: a multinational, interprofessional perspective | Intensive Care Medicine. 2017; 43(9):1329-1339. | Excluded | Based on title and abstract |
| 776 | Pant D, 2014 | Comparison of sublingual midazolam and dexmedetomidine for premedication in children | Minerva Anestesiol. 2014; 80(2):167-175. | Excluded | Based on title and abstract |
| 777 | Paramasivan VK, 2016 | A simple economical protocol for drug induced sleep endoscopy in OSAS | Otolaryngology - Head and Neck Surgery (United States). 2016; 155:P164. | Excluded | Based on title and abstract |
| 778 | Park B, 2017 | Effect of nasal high flow system on oxygenation during sedation under spinal anesthesia in elderly patients | Respirology (Carlton, Vic.). 2017; 22:228. | Excluded | Based on title and abstract |
| 779 | Parkar A, 2019 | Effect of prolonged sedation with midazolam and dexmedetomidine on sleep homeostasis in rats | Journal of Neurosurgical Anesthesiology. 2019; 31(4):489-490. | Excluded | Based on title and abstract |
| 780 | Parra CC, 2021 | A feasible and safe alternative: Radiologically inserted gastrostomy with limited sedation for people with amyotrophic lateral sclerosis | Amyotrophic Lateral Sclerosis and Frontotemporal Degeneration. 2021; 22(SUPPL 2):222. | Excluded | Based on title and abstract |
| 781 | Parthasarathy S, 2015 | Effects of central α2 adrenergic agent on sleep and inflammation in critically ill patients with acute respiratory distress syndrome | American Journal of Respiratory and Critical Care Medicine. 2015; 191. | Excluded | Based on title and abstract |
| 782 | Pasolini MP, 2013 | Acepromazine-dexmedetomidine-ketamine for injectable anaesthesia in captive European brown hares (Lepus europaeus) | Vet Anaesth Analg. 2013; 40(6):610-614. | Excluded | Based on title and abstract |
| 783 | Patel A, 2010 | Dexmedetomidine infusion for analgesia and prevention of emergence agitation in children with obstructive sleep apnea syndrome undergoing tonsillectomy and adenoidectomy | Anesth Analg. 2010; 111(4):1004-1010. | Excluded | Based on title and abstract |
| 784 | Patel M, 2008 | Sleep in the intensive care unit setting | Critical Care Nursing Quarterly. 2008; 31(4):309-318. | Excluded | Based on title and abstract |
| 785 | Patino M, 2012 | Obstructive sleep apnea syndrome: Anesthetic concerns | International Anesthesiology Clinics. 2012; 50(4):41-53. | Excluded | Based on title and abstract |
| 786 | Patino M, 2013 | Pediatric sedation outside the operating room | International Anesthesiology Clinics. 2013; 51(2):127-146. | Excluded | Based on title and abstract |
| 787 | Patla SDK, 2019 | Is it necessary to perform drug-induced sleep endoscopy in severe obstructive sleep apnea? | Clinical Rhinology. 2019; 12(1):3-5. | Excluded | Based on title and abstract |
| 788 | Paul BS, 2013 | Sedation in neurological intensive care unit | Annals of Indian Academy of Neurology. 2013; 16(2):194-202. | Excluded | Based on title and abstract |
| 789 | Pearson SD, 2020 | Evolving targets for sedation during mechanical ventilation | Current Opinion in Critical Care. 2020; 26(1):47-52. | Excluded | Based on title and abstract |
| 790 | Pedemonte JC, 2020 | Electroencephalogram burst-suppression during cardiopulmonary bypass in elderly patients mediates postoperative delirium | Anesthesiology. 2020; 133(2):280-292. | Excluded | Based on title and abstract |
| 791 | Peeters-Asdourian C, 2017 | Pain control in thoracic oncology | European Respiratory Journal. 2017; 50(3). | Excluded | Based on title and abstract |
| 792 | Peitz GJ, 2013 | Top 10 myths regarding sedation and delirium in the ICU | Critical Care Medicine. 2013; 41(9 SUPPL.1):S46-S56. | Excluded | Based on title and abstract |
| 793 | Peng H, 2017 | Acoustic analysis of snoring sounds originating from different sources determined by drug-induced sleep endoscopy | Acta Otolaryngol. 2017; 137(8):872-876. | Excluded | Based on title and abstract |
| 794 | Peng J, 2022 | Letter to the editor regarding "reduced pain and opioid use in the early postoperative period in patients undergoing a frontotemporal craniotomy under regional versus general anesthesia" | World Neurosurgery. 2022; 157:243. | Excluded | Based on title and abstract |
| 795 | Peng K, 2017 | Optimization of postoperative intravenous patient-controlled analgesia with opioid-dexmedetomidine combinations: An updated meta-analysis with trial sequential analysis of randomized controlled trials | Pain Physician. 2017; 20(7):569-595. | Excluded | Based on title and abstract |
| 796 | Peruzzi WT, 2005 | Approach to sedation in the ICU | Seminars in Anesthesia, Perioperative Medicine and Pain. 2005; 24(1):27-33. | Excluded | Based on title and abstract |
| 797 | Pestieau SR, 2011 | High-dose dexmedetomidine increases the opioid-free interval and decreases opioid requirement after tonsillectomy in children | Can J Anaesth. 2011; 58(6):540-550. | Excluded | Based on title and abstract |
| 798 | Ping Y, 2017 | Dexmedetomidine as an adjuvant to local anesthetics in brachial plexus blocks a meta-analysis of randomized controlled trials | Medicine (United States). 2017; 96(4). | Excluded | Based on title and abstract |
| 799 | Pirbudak L, 2014 | Pain management with intrathecal clonidine in a colon cancer patient with opioid hyperalgesia: case presentation | Agri. 2014; 26(2):93-96. | Excluded | Based on title and abstract |
| 800 | Pisani MA, 2019 | Pain and delirium in critical illness: an exploration of key 2018 SCCM PADIS guideline evidence gaps | Seminars in Respiratory and Critical Care Medicine. 2019; 40(5):604-613. | Excluded | Based on title and abstract |
| 801 | Plunkett AR, 2009 | Awake thyroidectomy under local anesthesia and dexmedetomidine infusion | Mil Med. 2009; 174(1):100-102. | Excluded | Based on title and abstract |
| 802 | Porhomayon J, 2013 | Strategies in postoperative analgesia in the obese obstructive sleep apnea patient | Clinical Journal of Pain. 2013; 29(11):998-1005. | Excluded | Based on title and abstract |
| 803 | Porhomayon J, 2014 | Respiratory perioperative management of patients with obstructive sleep apnea | Journal of Intensive Care Medicine. 2014; 29(3):145-153. | Excluded | Based on title and abstract |
| 804 | Porterfield L, 2021 | Distinct phenotypes of oropharyngeal obstruction on adult DISE | Otolaryngology - Head and Neck Surgery. 2021; 165(1 SUPPL):P163-P164. | Excluded | Based on title and abstract |
| 805 | Porterfield L, 2021 | OSA characteristics and DISE patterns in males and females | Otolaryngology - Head and Neck Surgery. 2021; 165(1 SUPPL):P352. | Excluded | Based on title and abstract |
| 806 | Porterfield L, 2022 | Sleep endoscopy patterns in six adults with trisomy 21 and obstructive sleep apnea | Laryngoscope. 2022; 132(2):485-487. | Excluded | Based on title and abstract |
| 807 | Power I, 1999 | Analgesic agents for the postoperative period: Nonopioids | Surgical Clinics of North America. 1999; 79(2):275-295. | Excluded | Based on title and abstract |
| 808 | Prabhahar T, 2018 | Analgesia, sedation and delirium (ASD) in critical care | Sri Lankan Journal of Anaesthesiology. 2018; 26(2):82-88. | Excluded | Based on title and abstract |
| 809 | Prashar H, 2018 | Laparoscopic bariatric surgery of super-obese patient with intractable anterior abdominal wall cellulitis: A rare challenge to anaesthesiologist | Journal of Clinical and Diagnostic Research. 2018; 12(7):UD01-UD03. | Excluded | Based on title and abstract |
| 810 | Praud JP, 2019 | Sleep-disordered breathing in children: Which study and when? | Pediatric Pulmonology. 2019; 54:S41-A43. | Excluded | Based on title and abstract |
| 811 | Qu JZ, 2020 | Perioperative cognitive function: Must the poor get poorer? | Minerva Anestesiologica. 2020; 86(3):368-370. | Excluded | Based on title and abstract |
| 812 | Quintard H, 2016 | Dexmedetomidine, a revolution for sedation in ICU? | Anaesthesia Critical Care and Pain Medicine. 2016; 35(1):5-6. | Excluded | Based on title and abstract |
| 813 | Raeder J, 2011 | Ambulatory anesthesia aspects for tonsillectomy and abrasion in children | Current Opinion in Anaesthesiology. 2011; 24(6):620-626. | Excluded | Based on title and abstract |
| 814 | Raiker NK, 2018 | Early initiation of venovenous extracorporeal membrane oxygenation in a mechanically ventilated patient with severe acute respiratory distress syndrome | BMJ Case Reports. 2018; 2018. | Excluded | Based on title and abstract |
| 815 | Ramasubbu C, 2011 | Pharmacological treatment of opioid-induced hyperalgesia: A review of the evidence | Journal of Pain and Palliative Care Pharmacotherapy. 2011; 25(3):219-230. | Excluded | Based on title and abstract |
| 816 | Ramaswamy SM, 2021 | Dexmedetomidine-induced deep sedation mimics non-rapid eye movement stage 3 sleep: large-scale validation using machine learning | Sleep. 2021; 44(2). | Excluded | Based on title and abstract |
| 817 | Rampes S, 2019 | Postoperative sleep disorders and their potential impacts on surgical outcomes | J Biomed Res. 2019; 34(4):271-280. | Excluded | Based on title and abstract |
| 818 | Ramsay MA, 2006 | Tracheal resection in the morbidly obese patient: the role of dexmedetomidine | J Clin Anesth. 2006; 18(6):452-454. | Excluded | Based on title and abstract |
| 819 | Ramsay MAE, 2006 | Sedation for the patient in the critical care unit: Current and future strategies | Anaesthesiology and Intensive Care. 2006; 33(3):3-8. | Excluded | Based on title and abstract |
| 820 | Ran J, 2021 | Role of dexmedetomidine in early POCD in patients undergoing thoracic surgery | BioMed research international. 2021; 2021:8652028. | Excluded | Based on title and abstract |
| 821 | Ranallo CD, 2013 | Sleep and mechanical ventilation in the intensive care unit | Journal of Pediatric Intensive Care. 2013; 2(1):5-9. | Excluded | Based on title and abstract |
| 822 | Rao S, 2021 | Electroencephalographic characteristics associated with the use of dexmedetomidine during supratentorial craniotomies | Journal of Neurosurgical Anesthesiology. 2021; 33(4):403-404. | Excluded | Based on title and abstract |
| 823 | Rascon-Martinez DM, 2018 | Advantages of ketamine as a perioperative analgesic | Revista Medica del Hospital General de Mexico. 2018; 81(4):253-261. | Excluded | Based on title and abstract |
| 824 | Rathier MO, 2011 | A review of recent clinical trials and guidelines on the prevention and management of delirium in hospitalized older patients | Hosp Pract (1995). 2011; 39(4):96-106. | Excluded | Based on title and abstract |
| 825 | Reade MC, 2010 | Optimal sedation for the ventilation of critically ill patients | Current Respiratory Medicine Reviews. 2010; 6(4):292-299. | Excluded | Based on title and abstract |
| 826 | Reade MC, 2016 | Low dose dexmedetomidine for the prophylaxis of perioperative ICU delirium-how much evidence is enough? | Journal of Thoracic Disease. 2016; 8(11):3020-3023. | Excluded | Based on title and abstract |
| 827 | Reed RN, 2020 | Acute pain in the trauma patient | Current Trauma Reports. 2020; 6(4):147-153. | Excluded | Based on title and abstract |
| 828 | Reel B, 2022 | Dexmedetomidine | StatPearls. 2022. | Excluded | Based on title and abstract |
| 829 | Reid K, 1994 | Chronic administration of an α2 adrenergic agonist desensitizes rats to the anesthetic effects of dexmedetomidine | Pharmacology Biochemistry and Behavior. 1994; 47(1):171-175. | Excluded | Based on title and abstract |
| 830 | Rekatsina M, 2021 | Effects of intravenous dexmedetomidine versus lidocaine on postoperative pain, analgesic consumption and functional recovery after abdominal gynecological surgery: A randomized placebo-controlled double blind study | Pain Physician. 2021; 24(7):E997-e1006. | Excluded | Based on title and abstract |
| 831 | Ren C, 2015 | Effect of intraoperative and postoperative infusion of dexmedetomidine on the quality of postoperative analgesia in highly nicotine-dependent patients after thoracic surgery: a CONSORT-prospective, randomized, controlled Trial | Medicine. 2015; 94(32):e1329. | Excluded | Based on title and abstract |
| 832 | Ren WHP, 2009 | Anesthetic management of epileptic pediatric patients | International Anesthesiology Clinics. 2009; 47(3):101-116. | Excluded | Based on title and abstract |
| 833 | Ren X, 2021 | Dysfunction of the glymphatic system as a potential mechanism of perioperative neurocognitive disorders | Frontiers in Aging Neuroscience. 2021; 13. | Excluded | Based on title and abstract |
| 834 | Ren Z, 2022 | Efficacy of intraoperative sedation combined with preemptive analgesia for single-level kyphoplasty under local anesthesia: A randomized clinical trial | Journal of Orthopaedic Science. 2022; 27(6):1215-1221. | Excluded | Based on title and abstract |
| 835 | Rettig RL, 2022 | Same day discharge for pectus excavatum-is it possible? | Journal of Pediatric Surgery. 2022; 57(9):34-38. | Excluded | Based on title and abstract |
| 836 | Richa F, 2015 | Dexmedetomidine sedation for a claustrophobic patient with obstructive sleep apnea undergoing magnetic resonance imaging | Journal of Clinical Anesthesia. 2015; 27(8):698-699. | Excluded | Based on title and abstract |
| 837 | Richardson AG, 2017 | Hippocampal gamma-slow oscillation coupling in macaques during sedation and sleep | Hippocampus. 2017; 27(11):1125-1139. | Excluded | Based on title and abstract |
| 838 | Richman M, 2022 | A pipe dream? Opioid-free anesthesia in pediatric urology | Journal of Urology. 2022; 207(SUPPL 5):e293. | Excluded | Based on title and abstract |
| 839 | Ridgway R, 2019 | Update on ENT anaesthesia in children | Anaesthesia and Intensive Care Medicine. 2019; 20(1):56-60. | Excluded | Based on title and abstract |
| 840 | Riker RR, 2005 | Adverse events associated with sedatives, analgesics, and other drugs that provide patient comfort in the intensive care unit | Pharmacotherapy. 2005; 25(5 II):8S-18S. | Excluded | Based on title and abstract |
| 841 | Roberts S, 2016 | Peripheral and local anaesthetic techniques for paediatric surgery | Anaesthesia and Intensive Care Medicine. 2016; 17(6):286-292. | Excluded | Based on title and abstract |
| 842 | Rocans RP, 2021 | Patient satisfaction and safety of sedation using perineurial dexmedetomidine in axillary brachial plexus block for wrist surgery | Regional anesthesia and pain medicine. 2021; 70(SUPPL 1):A83. | Excluded | Based on title and abstract |
| 843 | Rocans RP, 2022 | Perineural administration of dexmedetomidine in axillary brachial plexus block provides safe and comfortable sedation: A randomized clinical trial | Front Med (Lausanne). 2022; 9:834778. | Excluded | Based on title and abstract |
| 844 | Roche D, 2021 | Depth of anesthesia monitoring | Anesthesiology Clinics. 2021; 39(3):477-492. | Excluded | Based on title and abstract |
| 845 | Rodrigues L, 2022 | Dexmedetomidine combined with propofol as a sedation protocol for drug-induced sleep endoscopy | Otolaryngology - Head and Neck Surgery. 2022; 167(1):P293-P294. | Excluded | Based on title and abstract |
| 846 | Romagnoli S, 2018 | Light sedation with dexmedetomidine: a practical approach for the intensivist in different ICU patients | Minerva Anestesiol. 2018; 84(6):731-746. | Excluded | Based on title and abstract |
| 847 | Romagnoli S, 2018 | Patients, families and intensive care unit-staff members: From sedation strategies to global interaction for stress control | Minerva Anestesiologica. 2018; 84(10):1120-1122. | Excluded | Based on title and abstract |
| 848 | Romero N, 2021 | Evaluation of delirium in critically ill patients prescribed melatonin or ramelteon | Annals of Pharmacotherapy. 2021; 55(11):1347-1354. | Excluded | Based on title and abstract |
| 849 | Romo S, 2021 | General anesthesia in Kleine Levin Syndrome. Is it possible? A case report | Anesthesia and Analgesia. 2021; 133(3 SUPPL 2):825. | Excluded | Based on title and abstract |
| 850 | Rossi MG, 2009 | New modalities and paradigms for sedation: "new sedation agents" | Techniques in Gastrointestinal Endoscopy. 2009; 11(4):171-176. | Excluded | Based on title and abstract |
| 851 | Rozet I, 2008 | Anesthesia for functional neurosurgery: the role of dexmedetomidine | Curr Opin Anaesthesiol. 2008; 21(5):537-543. | Excluded | Based on title and abstract |
| 852 | Rozet I, 2021 | Multimodal monitoring of arousal, or just call your patient by name | British Journal of Anaesthesia. 2021; 127(2):170-172. | Excluded | Based on title and abstract |
| 853 | Rudikoff AG, 2022 | Perioperative acetaminophen and dexmedetomidine eliminate post-operative opioid requirement following pediatric tonsillectomy | J Clin Med. 2022; 11(3). | Excluded | Based on title and abstract |
| 854 | Rudra A, 2010 | Obstructive sleep apnoea syndrome in children and anaesthesia | Indian Journal of Anaesthesia. 2010; 54(1):18-23. | Excluded | Based on title and abstract |
| 855 | Rutledge R, 2013 | Opioid sparing anesthesia in minigastric bypass using dexmedetomidine, ketamine and total intravenous anesthesia (TIVA) | Obesity Surgery. 2013; 23(6):840. | Excluded | Based on title and abstract |
| 856 | Rutman MS, 2009 | Sedation for emergent diagnostic imaging studies in pediatric patients | Current Opinion in Pediatrics. 2009; 21(3):306-312. | Excluded | Based on title and abstract |
| 857 | Sa Rego MM, 1997 | Monitored anesthesia care: An alternative to general and regional anesthesia | Current Opinion in Anaesthesiology. 1997; 10(6):430-437. | Excluded | Based on title and abstract |
| 858 | Sa Rego MM, 1997 | The changing role of monitored anesthesia care in the ambulatory setting | Anesthesia and Analgesia. 1997; 85(5):1020-1036. | Excluded | Based on title and abstract |
| 859 | Saadawy I, 2009 | Effect of dexmedetomidine on the characteristics of bupivacaine in a caudal block in pediatrics | Acta Anaesthesiol Scand. 2009; 53(2):251-256. | Excluded | Based on title and abstract |
| 860 | Sairaku A, 2014 | Procedural sedation with dexmedetomidine during ablation of atrial fibrillation: a randomized controlled trial | Europace. 2014; 16(7):994-999. | Excluded | Based on title and abstract |
| 861 | Saito J, 2018 | Usefulness of dexmedetomidine to prevent emergence agitation in a patient with Krabbe disease: a case report | JA Clin Rep. 2018; 4(1):34. | Excluded | Based on title and abstract |
| 862 | Salgado DR, 2013 | Control of hypertension in the critically ill: A pathophysiological approach | Annals of Intensive Care. 2013; 3(1):1-13. | Excluded | Based on title and abstract |
| 863 | Salik I, 2011 | Dexmedetomidine in emergence agitation in children undergoing general anesthesia for interventional radiology | Journal of Neurosurgical Anesthesiology. 2011; 23(4):436. | Excluded | Based on title and abstract |
| 864 | Saliski M, 2015 | Optimizing sedation management to promote early mobilization for critically ill children | Journal of Pediatric Intensive Care. 2015; 4(4):188-193. | Excluded | Based on title and abstract |
| 865 | Sanders MO, 2021 | A systematic review of anaesthetic agents used in Drug Induced Sedation Endoscopy (DISE) and a description of a new DISE technique | 2021 | Excluded | Based on title and abstract |
| 866 | Sanders RD, 2007 | α2-Adrenoceptor agonists | Current Opinion in Investigational Drugs. 2007; 8(1):25-33. | Excluded | Based on title and abstract |
| 867 | Sanders RD, 2011 | Contribution of sedative-hypnotic agents to delirium via modulation of the sleep pathway | Canadian Journal of Anesthesia. 2011; 58(2):149-156. | Excluded | Based on title and abstract |
| 868 | Sanders RD, 2013 | Impact of anaesthetics and surgery on neurodevelopment: An update | British Journal of Anaesthesia. 2013; 110(SUPPL.1):i53-i72. | Excluded | Based on title and abstract |
| 869 | Sanders RD, 2016 | Is consciousness fragile? | British Journal of Anaesthesia. 2016; 116(1):1-3. | Excluded | Based on title and abstract |
| 870 | Sandiumenge A, 2010 | Sedation of ventilated patients and analgesia | Clinical Pulmonary Medicine. 2010; 17(6):290-299. | Excluded | Based on title and abstract |
| 871 | Sandvik RM, 2022 | Prospective measurement of lung function in 0-4-year-old danish children | Pediatric Pulmonology. 2022; 57:S89. | Excluded | Based on title and abstract |
| 872 | Sandvik RM, 2022 | Prospective measurement of lung function in 0-4-year-old danish children with cystic fibrosis | Journal of Cystic Fibrosis. 2022; 21:S37. | Excluded | Based on title and abstract |
| 873 | Sanford DT, 2012 | Systemic administration of eszopiclone depresses ventilation in a rat model of metabolic syndrome | Sleep. 2012; 35:A55-A56. | Excluded | Based on title and abstract |
| 874 | Saniasiaya J, 2020 | Outcome of drug induced sleep endoscopy directed surgery in paediatrics obstructive sleep apnoea: A systematic review | International Journal of Pediatric Otorhinolaryngology. 2020; 139. | Excluded | Based on title and abstract |
| 875 | Sankaran S, 2016 | Sleep disordered breathing as a risk factor for emergence delirium in pediatric patients undergoing ambulatory surgery | Anesthesia and Analgesia. 2016; 122(5):S242. | Excluded | Based on title and abstract |
| 876 | Sathishkumar S, 2007 | Management of emergence agitation | Anaesthesia. 2007; 62(5):530. | Excluded | Based on title and abstract |
| 877 | Saudek DE, 2022 | Intranasal dexmedetomidine: the ideal drug for sedation in the pediatric echo lab? | Cardiology in the Young. 2022; 32(4):545-549. | Excluded | Based on title and abstract |
| 878 | Savola JM, 1991 | Central α2-adrenoceptors are highly stereoselective for dexmedetomidine, the dextro enantiomer of medetomidine | European Journal of Pharmacology. 1991; 195(2):193-199. | Excluded | Based on title and abstract |
| 879 | Savvidou A, 2022 | Drug-induced hyperthermia with rhabdomyolysis in CLN3 disease | European Journal of Paediatric Neurology. 2022; 39:74-78. | Excluded | Based on title and abstract |
| 880 | Saxena S, 2018 | Impact on the brain of the inflammatory response to surgery | Presse Medicale. 2018; 47(4P2):e73-e81. | Excluded | Based on title and abstract |
| 881 | Scheinin A, 2021 | Foundations of human consciousness: Imaging the twilight zone | Journal of Neuroscience. 2021; 41(8):1769-1778. | Excluded | Based on title and abstract |
| 882 | Schiltz B, 2016 | Pentobarbital coma for rapid withdrawal and detoxification | Critical Care Medicine. 2016; 44(12):539. | Excluded | Based on title and abstract |
| 883 | Schipmann S, 2016 | Awake-awake-awake craniotomies in glioma surgery | Neuro-Oncology. 2016; 18:vi192. | Excluded | Based on title and abstract |
| 884 | Schug SA, 2011 | Postoperative pain management of the obese patient | Best Practice and Research: Clinical Anaesthesiology. 2011; 25(1):73-81. | Excluded | Based on title and abstract |
| 885 | Schuler BR, 2021 | Evaluating the safety of continuous infusion lidocaine for postoperative pain | Clinical Journal of Pain. 2021; 37(9):657-663. | Excluded | Based on title and abstract |
| 886 | Schulz U, 2006 | Asleep-awake-asleep-anaesthetic technique for awake craniotomy | Anaesthesist. 2006; 55(5):585-598. | Excluded | Based on title and abstract |
| 887 | Schumann R, 2011 | Anaesthesia for bariatric surgery | Best Practice and Research: Clinical Anaesthesiology. 2011; 25(1):83-93. | Excluded | Based on title and abstract |
| 888 | Schwartz AC, 2016 | Pharmacologic and nonpharmacologic approaches to the prevention and management of delirium | International Journal of Psychiatry in Medicine. 2016; 51(2):160-170. | Excluded | Based on title and abstract |
| 889 | Schwengel DA, 2009 | Perioperative management of children with obstructive sleep apnea | Anesthesia and Analgesia. 2009; 109(1):60-75. | Excluded | Based on title and abstract |
| 890 | Seamon ML, 2021 | Hypertension in Pheochromocytoma and Paraganglioma: Evaluation and Management in Pediatric Patients | Current Hypertension Reports. 2021; 23(5). | Excluded | Based on title and abstract |
| 891 | Seet E, 2010 | Obstructive sleep apnea: Preoperative assessment | Anesthesiology Clinics. 2010; 28(2):199-215. | Excluded | Based on title and abstract |
| 892 | Seet E, 2011 | Airway management in obstructive sleep apnea: Local solutions - Reply | Canadian Journal of Anesthesia. 2011; 58(2):229. | Excluded | Based on title and abstract |
| 893 | Seidel WF, 1995 | Alpha-2 adrenergic modulation of sleep: Time-of-day-dependent pharmacodynamic profiles of dexmedetomidine and clonidine in the rat | Journal of Pharmacology and Experimental Therapeutics. 1995; 275(1):263-273. | Excluded | Based on title and abstract |
| 894 | Selvadurai S, 2018 | Evaluating the effects of general anesthesia on sleep in children undergoing elective surgery: An observational case-control study | Sleep. 2018; 41(8). | Excluded | Based on title and abstract |
| 895 | Shah AN, 2007 | Dexmedetomidine related cardiac arrest in a patient with permanent pacemaker; a cautionary tale | PACE - Pacing and Clinical Electrophysiology. 2007; 30(9):1158-1160. | Excluded | Based on title and abstract |
| 896 | Shaikh M, 2021 | Delayed awakening after use of dexmedetomidine | Pakistan Journal of Medical and Health Sciences. 2021; 15(10):2701-2702. | Excluded | Based on title and abstract |
| 897 | Shaw R, 2022 | Comparative study between dexmeditomidine and midazolam for prevention of emergence agitation after nasal surgery | JK Science. 2022; 24(2):75-80. | Excluded | Based on title and abstract |
| 898 | Shelton KT, 2018 | Minimizing ICU neurological dysfunction with dexmedetomidine-induced sleep (MINDDS): Protocol for a randomised, double-blind, parallel-arm, placebo-controlled trial | BMJ Open. 2018; 8(4):e020316. | Excluded | Based on title and abstract |
| 899 | Shen F, 2022 | Effect of intranasal dexmedetomidine or midazolam for premedication on the occurrence of respiratory adverse events in children undergoing tonsillectomy and adenoidectomy: A randomized clinical trial | JAMA Netw Open. 2022; 5(8):e2225473. | Excluded | Based on title and abstract |
| 900 | Shepard SM, 2011 | Dexmedetomidine-related atrial standstill and loss of capture in a pediatric patient after congenital heart surgery | Crit Care Med. 2011; 39(1):187-189. | Excluded | Based on title and abstract |
| 901 | Sher Y, 2017 | Delirium after lung transplantation: Association with recipient characteristics, hospital resource utilization, and mortality | Clinical Transplantation. 2017; 31(5). | Excluded | Based on title and abstract |
| 902 | Sher Y, 2020 | COVID-19-Associated hyperactive intensive care unit delirium with proposed pathophysiology and treatment: A case report | Psychosomatics. 2020; 61(5):544-550. | Excluded | Based on title and abstract |
| 903 | Shi CM, 2016 | Effects of dexmedetomidine combined with propofol on painless gastroscopy and colonoscopy in the elderly | Chinese Journal of New Drugs. 2016; 25(19):2229-2233. | Excluded | Based on title and abstract |
| 904 | Shi XX, 2017 | Safety and effectiveness of dexmedetomidine combined with butorphanol in patient- controlled intravenous analgesia in elderly patients treated with proximal femoral nail anti- rotation | Journal of International Translational Medicine. 2017; 5(4):212-216. | Excluded | Based on title and abstract |
| 905 | Shim JJ, 2012 | An update on delirium in the postoperative setting: Prevention, diagnosis and management | Best Practice and Research: Clinical Anaesthesiology. 2012; 26(3):327-343. | Excluded | Based on title and abstract |
| 906 | Shimabukuro A, 2007 | Airway management with dexmedetomidine for difficult airway | Masui. 2007; 56(6):681-684. | Excluded | Based on title and abstract |
| 907 | Shin HJ, 2018 | Comparison of upper airway patency in patients with mild obstructive sleep apnea during dexmedetomidine or propofol sedation: a prospective, randomized, controlled trial | BMC Anesthesiol. 2018; 18(1):120. | Excluded | Based on title and abstract |
| 908 | Shionoya Y, 2019 | Intravenous sedation in arnold-chiari malformation with respiratory failure | Anesth Prog. 2019; 66(1):37-41. | Excluded | Based on title and abstract |
| 909 | Shott SR, 2011 | Evaluation and management of pediatric obstructive sleep apnea beyond tonsillectomy and adenoidectomy | Current Opinion in Otolaryngology and Head and Neck Surgery. 2011; 19(6):449-454. | Excluded | Based on title and abstract |
| 910 | Shott SR, 2012 | Sleep cine magnetic resonance imaging-A dynamic evaluation of the airway | Operative Techniques in Otolaryngology - Head and Neck Surgery. 2012; 23(1):19-24. | Excluded | Based on title and abstract |
| 911 | Shteamer JW, 2017 | Sedative choice in drug-induced sleep endoscopy: A neuropharmacology-based review | Laryngoscope. 2017; 127(1):273-279. | Excluded | Based on title and abstract |
| 912 | Sieber FE, 2011 | Preventing postoperative complications in the elderly | Anesthesiology Clinics. 2011; 29(1):83-97. | Excluded | Based on title and abstract |
| 913 | Siegmann MJ, 2019 | Dexmedetomidine causes age-dependent differences in the EEG compared to NREM sleep in b6 mice | Sleep. 2019; 42:A51-A52. | Excluded | Based on title and abstract |
| 914 | Simone C, 2023 | Treatment of septic encephalopathy and encephalitis - a critical appraisal | Expert Rev Neurother. 2023; 23(12):1069-1080. | Excluded | Based on title and abstract |
| 915 | Singh A, 2021 | Analgesic efficacy and safety of dexmedetomidine as an adjuvant to caudal levobupivacaine for infraumbilical surgeries in children | Anestezi dergisi. 2021; 29(4):263‐269. | Excluded | Based on title and abstract |
| 916 | Singh G, 2010 | Cognitive impairment in the ICU: Could more have been done for this elder? | Journal of the American Geriatrics Society. 2010; 58:S136. | Excluded | Based on title and abstract |
| 917 | Singla N, 2008 | Treatment of acute postoperative hypertension in cardiac surgery patients: an efficacy study of clevidipine assessing its postoperative antihypertensive effect in cardiac surgery-2 (ESCAPE-2), a randomized, double-blind, placebo-controlled trial | Anesthesia and analgesia. 2008; 107(1):59‐67. | Excluded | Based on title and abstract |
| 918 | Sinha A, 2012 | Pain management in morbidly obese patients undergoing laparoscopic bariatric surgery : Our experience | Obesity Surgery. 2012; 22(9):1353. | Excluded | Based on title and abstract |
| 919 | Situ, 2020 | Perioperative sedation and sympatholysis due to tizanidine | Journal of Clinical and Diagnostic Research. 2020; 14(9):UD01-UD02. | Excluded | Based on title and abstract |
| 920 | Siyam M, 2007 | Anaesthetic management of adult patients with obstructive sleep apnea syndrome | Annales Francaises d'Anesthesie et de Reanimation. 2007; 26(1):39-52. | Excluded | Based on title and abstract |
| 921 | Skrobik Y, 2009 | Delirium prevention and treatment | Critical Care Clinics. 2009; 25(3):585-591. | Excluded | Based on title and abstract |
| 922 | Skrobik Y, 2016 | To sleep, perhaps to dream sedatives and the uncertainties surrounding therapeutic choices in critical care | Chest. 2016; 149(6):1355-1356. | Excluded | Based on title and abstract |
| 923 | Skrobik Y, 2017 | Impact of nocturnal dexmedetomidine on delirium incidence and sleep quality in critically ill adults: A randomized, double-blind, placebo-controlled trial | American Journal of Respiratory and Critical Care Medicine. 2017; 195. | Excluded | Based on title and abstract |
| 924 | Sleigh JW, 2011 | General anaesthesia and electroencephalographic spindles | Trends in Anaesthesia and Critical Care. 2011; 1(5-6):263-269. | Excluded | Based on title and abstract |
| 925 | Smally AJ, 2011 | Procedural sedation and analgesia in the emergency department | Current Opinion in Critical Care. 2011; 17(4):317-322. | Excluded | Based on title and abstract |
| 926 | Smith DF, 2020 | Effectiveness of pediatric drug-induced sleep endoscopy for REM-predominant obstructive sleep apnea | Sleep Breath. 2020; 24(4):1705-1713. | Excluded | Based on title and abstract |
| 927 | Smith HAB, 2022 | 2022 Society of critical care medicine clinical practice guidelines on prevention and management of pain, agitation, neuromuscular blockade, and delirium in critically ill pediatric patients with consideration of the ICU environment and early mobility | Pediatric Critical Care Medicine. 2022; 23(2):E74-E110. | Excluded | Based on title and abstract |
| 928 | Smith HB, 2013 | Pediatric critical care perceptions on analgesia, sedation, and delirium | Seminars in Respiratory and Critical Care Medicine. 2013; 34(2):244-261. | Excluded | Based on title and abstract |
| 929 | Smithburger PL, 2019 | Pharmacologic considerations surrounding sedation, delirium, and sleep in critically ill adults: A narrative review | Journal of Pharmacy Practice. 2019; 32(3):271-291. | Excluded | Based on title and abstract |
| 930 | Song F, 2018 | Effect of perioperative infusion of Dexmedetomidine combined with Sufentanil on quality of postoperative analgesia in patients undergoing laparoscopic nephrectomy: A CONSORT-prospective, randomized, controlled trial | BMC Anesthesiology. 2018; 18(1). | Excluded | Based on title and abstract |
| 931 | Song J, 2018 | Sleep and anesthesia | Sleep Medicine Research. 2018; 9(1):11-19. | Excluded | Based on title and abstract |
| 932 | Souter MJ, 2007 | Dexmedetomidine sedation during awake craniotomy for seizure resection: Effects on electrocorticography | Journal of Neurosurgical Anesthesiology. 2007; 19(1):38-44. | Excluded | Based on title and abstract |
| 933 | Spenard S, 2021 | Morphine or hydromorphone: Which should be preferred? A systematic review | Archives of Disease in Childhood. 2021; 106(10):1002-1009. | Excluded | Based on title and abstract |
| 934 | Spiller HA, 2013 | Overdose of drugs for attention-deficit hyperactivity disorder: Clinical presentation, mechanisms of toxicity, and management | CNS Drugs. 2013; 27(7):531-543. | Excluded | Based on title and abstract |
| 935 | Sreedhar N, 2017 | Bentall procedure in a patient with Parkinson disease | Annals of Cardiac Anaesthesia. 2017; 20(3):383-384. | Excluded | Based on title and abstract |
| 936 | Sriganesh K, 2020 | Anaesthesia for frameless stereotactic neurosurgery in a patient with Cheyne-Stokes respiration | Indian Journal of Anaesthesia. 2020; 64(10):913-915. | Excluded | Based on title and abstract |
| 937 | Starkey E, 2011 | Sedation for radiological imaging | Archives of Disease in Childhood: Education and Practice Edition. 2011; 96(3):101-106. | Excluded | Based on title and abstract |
| 938 | Steblaj B, 2022 | Effects of acepromazine and dexmedetomidine, followed by propofol induction and maintenance with isoflurane anaesthesia, on the microcirculation of Beagle dogs evaluated by sidestream dark field imaging: an experimental trial | Veterinary Anaesthesia and Analgesia. 2022; 49(4):364-371. | Excluded | Based on title and abstract |
| 939 | Steele J, 2022 | Evaluation of an opioid-free anesthesia protocol for elective abdominal surgery in a community hospital | AANA Journal. 2022; 9(3):215-223. | Excluded | Based on title and abstract |
| 940 | Stienstra R, 2016 | Pro-con debate: catheters and not adjuvants are the best way to prolong analgesia-pro | Regional anesthesia and pain medicine. 2016; 41(5):e30‐e31. | Excluded | Based on title and abstract |
| 941 | Su X, 2016 | Dexmedetomidine for prevention of delirium in elderly patients after non-cardiac surgery: a randomised, double-blind, placebo-controlled trial | Curr Opin Anaesthesiol. 2018; 31(1):83-88. | Excluded | Based on title and abstract |
| 942 | Su X, 2018 | Improve postoperative sleep: what can we do? | Lancet. 2016; 388(10054):1893‐1902. | Excluded | Based on title and abstract |
| 943 | Subramanyam R, 2016 | Upper airway morphology in Down Syndrome patients under dexmedetomidine sedation | Brazilian Journal of Anesthesiology. 2016; 66(4):388-394. | Excluded | Based on title and abstract |
| 944 | Sui X, 2021 | Postoperative delirium after long-term general anesthesia in elderly patients, how to reduce it?: Protocol of a double-blinded, randomized, placebo-controlled trial | Medicine (Baltimore). 2021; 100(22):e25885. | Excluded | Based on title and abstract |
| 945 | Suleman S, 2020 | Effects of sedative infusions on sleep features and background activity in pediatric TBI patients | Journal of Clinical Neurophysiology. 2020; 37(4):341. | Excluded | Based on title and abstract |
| 946 | Sullinger D, 2017 | Development, implementation, and outcomes of a delirium protocol in the surgical trauma intensive care unit | Annals of Pharmacotherapy. 2017; 51(1):5-12. | Excluded | Based on title and abstract |
| 947 | Sultana A, 2017 | Special indications for opioid free anaesthesia and analgesia, patient and procedure related: Including obesity, sleep apnoea, chronic obstructive pulmonary disease, complex regional pain syndromes, opioid addiction and cancer surgery | Best Pract Res Clin Anaesthesiol. 2017; 31(4):547-560. | Excluded | Based on title and abstract |
| 948 | Sury MRJ, 2005 | The management of infants and children for painless imaging | Clinical Radiology. 2005; 60(7):731-741. | Excluded | Based on title and abstract |
| 949 | Sury MRJ, 2008 | Deep sedation and minimal anesthesia | Paediatric Anaesthesia. 2008; 18(1):18-24. | Excluded | Based on title and abstract |
| 950 | Svider PF, 2018 | Perioperative analgesia for patients undergoing endoscopic sinus surgery: an evidence-based review | International Forum of Allergy and Rhinology. 2018; 8(7):837-849. | Excluded | Based on title and abstract |
| 951 | Sweidan AJ, 2019 | Recalcitrant central neurogenic hyperventilation: An unusual etiology and management | American Journal of Respiratory and Critical Care Medicine. 2019; 199(9). | Excluded | Based on title and abstract |
| 952 | Syeda S, 2020 | Dexmedetomidine as a primary systemic analgesic for craniotomy in an obese patient with obstructive sleep apnea | Neurol India. 2020; 68(2):507-508. | Excluded | Based on title and abstract |
| 953 | Syeda S, 2020 | Opioid free analgesia with dexmedetomidine for craniotomy in an obese patient with obstructive sleep apnea and difficult airway | Asian J Anesthesiol. 2020; 58(2):76-77. | Excluded | Based on title and abstract |
| 954 | Szmuk P, 2007 | Sedation and anesthesia for magnetic resonance imaging in pediatric patients: is dexmedetomidine the answer? | Seminars in Anesthesia, Perioperative Medicine and Pain. 2007; 26(4):229-236. | Excluded | Based on title and abstract |
| 955 | Taira T, 2019 | Usefulness of INTELLiVENT-ASV for postoperative ventilator-associated pneumonia: a case report | JA Clinical Reports. 2019; 5(1). | Excluded | Based on title and abstract |
| 956 | Takaya I, 2023 | Considerations for satisfactory sedation during dental implant surgery | J Pers Med. 2023; 13(3):36983643. | Excluded | Based on title and abstract |
| 957 | Talebi G, 2021 | Comparison of three different doses of dexmedetomidine added to bupivacaine in ultrasound-guided transversus abdominis plane block; a randomized clinical trial | Anesthesiology and Pain Medicine. 2021; 11(2). | Excluded | Based on title and abstract |
| 958 | Talon MD, 2009 | Intranasal dexmedetomidine premedication is comparable with midazolam in burn children undergoing reconstructive surgery | J Burn Care Res. 2009; 30(4):599-605. | Excluded | Based on title and abstract |
| 959 | Tamisier R, 2018 | Anesthesia and sleep apnea | Sleep Medicine Reviews. 2018; 40:79-92. | Excluded | Based on title and abstract |
| 960 | Tan J, 2021 | Effect of CT localization of upper airway obstruction site after inducing sleep on the value of obstructive sleep apnea hypopnea syndrome and the effect of surgery | Lin Chung Er Bi Yan Hou Tou Jing Wai Ke Za Zhi. 2021; 35(8):683-688. | Excluded | Based on title and abstract |
| 961 | Tanaka M, 2017 | Evaluation of influence of transient apnea and deep breathing recognized only during performing pulmonary vein isolation on maneuver of catheter ablation and recurrence of atrial fibrillation | Europace. 2017; 19:iii178-iii179. | Excluded | Based on title and abstract |
| 962 | Tang W, 2019 | Nasotracheal intubation-extubation-intubation and asleep-awake-asleep anesthesia technique for deep brain stimulation | BMC Anesthesiology. 2019; 19(1). | Excluded | Based on title and abstract |
| 963 | Tang W, 2020 | Effect of dexmedetomidine in children undergoing general anaesthesia with sevoflurane: a meta-analysis and systematic review | J Int Med Res. 2020; 48(6):300060520927530. | Excluded | Based on title and abstract |
| 964 | Tasso JJ, 2021 | Single shot subarachnoid anesthesia combined with NIV for prolonged resection of massive scrotal lymphedema in a morbidly obese patient | Anesthesia and Analgesia. 2021; 133(3 SUPPL 2):1819-1820. | Excluded | Based on title and abstract |
| 965 | Tedder A, 2014 | Profound hypertension with dexmedetomidine sedation for DBS surgery | Canadian Journal of Anesthesia. 2014; 61:S107. | Excluded | Based on title and abstract |
| 966 | Tedore TR, 2016 | Accidental infusion of phenylephrine infusion via popliteal catheter | Regional Anesthesia and Pain Medicine. 2016; 41(5). | Excluded | Based on title and abstract |
| 967 | Teegarden BMT, 2016 | Delirium prevention: Another piece of the puzzle | Journal of Thoracic Disease. 2016; 8(12):E1614-E1616. | Excluded | Based on title and abstract |
| 968 | Telias I, 2019 | Sleep and circadian rhythm in critical illness | Critical Care. 2019; 23(1). | Excluded | Based on title and abstract |
| 969 | Templeton D, 2022 | Early mobility in a tertiary pediatric intensive care unit | Journal of Investigative Medicine. 2022; 70(2):630. | Excluded | Based on title and abstract |
| 970 | Tenney JR, 2019 | Intranasal dexmedetomidine for sedation during magnetoencephalography | Journal of Clinical Neurophysiology. 2019; 36(5):371-374. | Excluded | Based on title and abstract |
| 971 | Terajima K, 2006 | Repeated dexmedetomidine infusions, a postoperative living-donor liver transplantation patient | J Anesth. 2006; 20(3):234-236. | Excluded | Based on title and abstract |
| 972 | Theuerkauf N, 2012 | Postoperative delirium in the PACU and intensive care unit | Trends in Anaesthesia and Critical Care. 2012; 2(4):148-155. | Excluded | Based on title and abstract |
| 973 | Thomas DA, 2016 | Efficacy and safety of nonopioid analgesics in perioperative pain control | Current Drug Safety. 2016; 11(3):196-205. | Excluded | Based on title and abstract |
| 974 | Thompson C, 2018 | Pain management within an enhanced recovery program after thoracic surgery | Journal of Thoracic Disease. 2018; 10:S3773-S3780. | Excluded | Based on title and abstract |
| 975 | Tian G, 2019 | Protective effects of dexmedetomidine on cognitive function in elderly patients with hip replacement | Latin American Journal of Pharmacy. 2019; 38(10):1963-1968. | Excluded | Based on title and abstract |
| 976 | Tian S, 2020 | Effects of dexmedetomidine combined with remifentanil anesthesia and propofol combined with remifentanil anesthesia on perioperative inflammatory response and pulmonary function in patients with lung cancer | Oncologie. 2020; 22(1):1-21. | Excluded | Based on title and abstract |
| 977 | Tobias JD, 2011 | Dexmedetomidine: Applications for the pediatric patient with congenital heart disease | Pediatric Cardiology. 2011; 32(8):1075-1087. | Excluded | Based on title and abstract |
| 978 | Tobias JD, 2014 | Clonidine infusions - Do they have a role in the PICU? | Pediatric Critical Care Medicine. 2014; 15(6):563-565. | Excluded | Based on title and abstract |
| 979 | Togawa E, 2019 | Dexmedetomidine and midazolam sedation reduces unexpected patient movement during dental surgery compared with propofol and midazolam sedation | Journal of Oral and Maxillofacial Surgery. 2019; 77(1):29-41. | Excluded | Based on title and abstract |
| 980 | Tolani KA, 2007 | Prevention and treatment of homeostatic disorders after central neurosurgical procedures | Best Practice and Research in Clinical Anaesthesiology. 2007; 21(4):539-556. | Excluded | Based on title and abstract |
| 981 | Tomita Y, 2022 | Sleep apnea and abnormal respiratory patterns with deep sedation during radiofrequency catheter ablation in patients with atrial fibrillation | Reviews in Cardiovascular Medicine. 2022; 23(4). | Excluded | Based on title and abstract |
| 982 | Torres JV, 2017 | Nonintubated video-assisted thoracoscopic surgery: A report of five cases | Journal of Cardiothoracic and Vascular Anesthesia. 2017; 31:S54. | Excluded | Based on title and abstract |
| 983 | Trabold B, 2014 | Postoperative delirium: Risk factors, prevention, and treatment | Journal of Cardiothoracic and Vascular Anesthesia. 2014; 28(5):1352-1360. | Excluded | Based on title and abstract |
| 984 | Traube C, 2014 | Neuroblastoma and pediatric delirium: A case series | Pediatric Blood and Cancer. 2014; 61(6):1121-1123. | Excluded | Based on title and abstract |
| 985 | Triantafillidis JK, 2013 | Sedation in gastrointestinal endoscopy: Current issues | World Journal of Gastroenterology. 2013; 19(4):463-481. | Excluded | Based on title and abstract |
| 986 | Tripathi A, 2016 | A comparative study of clonidine and dexmedetomidine as an adjunct to bupivacaine in supraclavicular brachial plexus block | Journal of Anaesthesiology Clinical Pharmacology. 2016; 32(3):344-348. | Excluded | Based on title and abstract |
| 987 | Tung A, 2005 | New anesthesia techniques | Thorac Surg Clin. 2005; 15(1):27-38. | Excluded | Based on title and abstract |
| 988 | Turkel SB, 2017 | Pediatric delirium: recognition, management, and outcome | Current Psychiatry Reports. 2017; 19(12). | Excluded | Based on title and abstract |
| 989 | Turner AD, 2021 | Cognitive dysfunction after analgesia and sedation: out of the operating room and into the pediatric intensive care unit | Frontiers in Behavioral Neuroscience. 2021; 15. | Excluded | Based on title and abstract |
| 990 | Ueno Y, 2022 | The quality and quantity of sleep on dexmedetomidine during high-flow nasal cannula oxygen therapy in critically ill patients | Journal of Medical Investigation. 2022; 69(3.4):266-272. | Excluded | Based on title and abstract |
| 991 | Umin, 2018 | The efficasy of dexmedetomidine in preoperaive sleep | https://trialsearch.who.int/Trial2.aspx?TrialID=JPRN-UMIN000032302. 2018. | Excluded | Based on title and abstract |
| 992 | Urner M, 2018 | Supportive care of patients on mechanical ventilation | Respiratory Care. 2018; 63(12):1567-1574. | Excluded | Based on title and abstract |
| 993 | Vacas S, 2013 | Sleep and anesthesia: Common mechanisms of action | Sleep Medicine Clinics. 2013; 8(1):1-9. | Excluded | Based on title and abstract |
| 994 | Valentine K, 2022 | Analgesia, sedation, paralytics, delirium, and latrogenic withdrawal | Pediatric Clinics of North America. 2022; 69(3):531-546. | Excluded | Based on title and abstract |
| 995 | Van Dort CJ, 2013 | Dexmedetomidine-induced sedation is similar to natural NREM sleep in mice | Sleep. 2013; 36:A7. | Excluded | Based on title and abstract |
| 996 | Varga MO, 2021 | Development and validation of a delirium risk prediction preoperative model for cardiac surgery patients (DELIPRECAS): An observational multicentre study | Journal of Clinical Anesthesia. 2021; 69. | Excluded | Based on title and abstract |
| 997 | Vargo JJ, 2009 | Procedural sedation and obesity: waters left uncharted | Gastrointestinal Endoscopy. 2009; 70(5):980-984. | Excluded | Based on title and abstract |
| 998 | Vargo JJ, 2011 | Sedation in the bariatric patient | Gastrointestinal Endoscopy Clinics of North America. 2011; 21(2):257-263. | Excluded | Based on title and abstract |
| 999 | Vaughns JD, 2017 | Dexmedetomidine as an adjuvant for perioperative pain management in adolescents undergoing bariatric surgery: An observational cohort study | J Pediatr Surg. 2017; 52(11):1787-1790. | Excluded | Based on title and abstract |
| 1000 | Verstraete S, 2012 | Post-cesarean section analgesia | Acta Anaesthesiologica Belgica. 2012; 63(4):147-167. | Excluded | Based on title and abstract |
| 1001 | Veselis RA, 2007 | Memory: a guide for anaesthetists | Best Practice and Research in Clinical Anaesthesiology. 2007; 21(3):297-312. | Excluded | Based on title and abstract |
| 1002 | Viana A, 2019 | The effect of sedating agents on drug-induced sleep endoscopy findings | Laryngoscope. 2019; 129(2):506-513. | Excluded | Based on title and abstract |
| 1003 | Vincent JL, 2017 | Optimizing sedation in the ICU: The eCASH concept | Signa Vitae. 2017; 13:10-13. | Excluded | Based on title and abstract |
| 1004 | Vitug S, 2020 | Sedation with ketamine and fentanyl combination improves patient outcomes in intensive care units | SN Comprehensive Clinical Medicine. 2020; 2(8):1139-1140. | Excluded | Based on title and abstract |
| 1005 | Vogt KN, 2014 | Maintaining comfort, cognitive function, and mobility in surgical intensive care unit patients | Journal of Trauma and Acute Care Surgery. 2014; 77(2):364-375. | Excluded | Based on title and abstract |
| 1006 | Vroegop AV, 2020 | Drug-induced sleep endoscopy: evaluation of a selection tool for treatment modalities for obstructive sleep apnea | Respiration. 2020; 99(5):451-457. | Excluded | Based on title and abstract |
| 1007 | Vukovic I, 2022 | Midazolam versus dexmedetomidine in patients at risk of obstructive sleep apnea during urology procedures: a randomized controlled trial | Journal of clinical medicine. 2022; 11(19). | Excluded | Based on title and abstract |
| 1008 | Wajekar AS, 2015 | Anaesthetic management for drainage of frontoparietal abscess in a patient of uncorrected tetralogy of fallot | Indian Journal of Anaesthesia. 2015; 59(4):244-246. | Excluded | Based on title and abstract |
| 1009 | Walia H, 2016 | Sevoflurane and bradycardia in infants with trisomy 21: A case report and review of the literature | International Journal of Pediatric Otorhinolaryngology. 2016; 80:5-7. | Excluded | Based on title and abstract |
| 1010 | Walsh J, 2014 | Drug induced natural sleep-dexmedetomidine versus propofol | Sleep and Biological Rhythms. 2014; 12:22. | Excluded | Based on title and abstract |
| 1011 | Walton JR, 2017 | The proceedings of the 15th professional conference on Williams Syndrome | American Journal of Medical Genetics, Part A. 2017; 173(5):1159-1171. | Excluded | Based on title and abstract |
| 1012 | Wampole CR, 2019 | Beyond opioids for pain management in adult critically ill patients | Journal of Pharmacy Practice. 2019; 32(3):256-270. | Excluded | Based on title and abstract |
| 1013 | Wang B, 2017 | Evaluation on postoperative analgesia efficacy of oxycodone combined with dexmedetomidine in patients underwent laparoscopic radical surgery of colon cancer | Journal of jilin university medicine edition. 2017; 43(6):1231‐1236. | Excluded | Based on title and abstract |
| 1014 | Wang D, 2021 | Effect of dexmedetomidine on postoperative delirium in patients undergoing brain tumour resections: study protocol of a randomised controlled trial | BMJ Open. 2021; 11(11):e051584. | Excluded | Based on title and abstract |
| 1015 | Wang D, 2022 | Opioid-free total intravenous anesthesia for thyroid and parathyroid surgery: Protocol for a randomized, double-blind, controlled trial | Frontiers in Medicine. 2022; 9. | Excluded | Based on title and abstract |
| 1016 | Wang DX, 2017 | In reply | Anesthesiology. 2017; 127(2):398-399. | Excluded | Based on title and abstract |
| 1017 | Wang G, 2020 | Dexmedetomidine alleviates sleep-restriction-mediated exaggeration of postoperative immunosuppression via splenic TFF2 in aged mice | Aging (Albany NY). 2020; 12(6):5318-5335. | Excluded | Based on title and abstract |
| 1018 | Wang GR, 2021 | Effect of oxycodone hydrochloride combined with dexmedetomidine on quality of recovery and stress response after general anesthesia in patients who had laparoscopic cholecystectomy | Pakistan Journal of Medical Sciences. 2021; 37(5):1408-1413. | Excluded | Based on title and abstract |
| 1019 | Wang H, 2020 | Perioperative sleep disturbances and postoperative delirium in adult patients: A systematic review and meta-analysis of clinical trials | Frontiers in Psychiatry. 2020; 11. | Excluded | Based on title and abstract |
| 1020 | Wang H, 2021 | Management of anesthesia in a patient with osteogenesis imperfecta and multiple fractures: a case report and review of the literature | Journal of International Medical Research. 2021; 49(6). | Excluded | Based on title and abstract |
| 1021 | Wang HL, 2015 | Doxapram hastens the recovery following total intravenous anesthesia with dexmedetomidine, propofol and remifentanil | Exp Ther Med. 2015; 9(4):1518-1522. | Excluded | Based on title and abstract |
| 1022 | Wang J, 2021 | Clinical effect of different drugs and infusion techniques for patient-controlled analgesia after spinal tumor surgery: A prospective, randomized, controlled clinical trial | Clin Ther. 2021; 43(6):1020-1028. | Excluded | Based on title and abstract |
| 1023 | Wang L, 2021 | A new understanding for preoperative management in a patient with blunt brachiocephalic trunk injury | Heart Surgery Forum. 2021; 24(2):E367-E378. | Excluded | Based on title and abstract |
| 1024 | Wang N, 2017 | The effects and mechanism of substance p2 in sedative and hypnotic of isoflurane mice | International Journal of Clinical and Experimental Medicine. 2017; 10(1):1539-1545. | Excluded | Based on title and abstract |
| 1025 | Wang W, 2022 | Dexmedetomidine infusion for emergence coughing prevention in patients undergoing an endovascular interventional procedure: A randomized dose-finding trial | Eur J Pharm Sci. 2022; 177:106230. | Excluded | Based on title and abstract |
| 1026 | Wang X, 2022 | A Response to: letter to the editor regarding "the effect of ultrasound-guided erector spinae plane block combined with dexmedetomidine on postoperative analgesia in patients undergoing modified radical mastectomy: A randomized controlled trial" | Pain and Therapy. 2022; 11(2):735-738. | Excluded | Based on title and abstract |
| 1027 | Wang XT, 2017 | Delirium in intensive care unit patients: Ten important points of understanding | Chinese Medical Journal. 2017; 130(20):2498-2502. | Excluded | Based on title and abstract |
| 1028 | Wang Y, 2019 | Risk factors and incidence of postoperative delirium in patients undergoing laryngectomy | J Pharm Pharm Sci. 2023; 26:11699. | Excluded | Based on title and abstract |
| 1029 | Wang Y, 2020 | Impact of intraoperative infusion and postoperative PCIA of dexmedetomidine on early breastfeeding after elective cesarean section: a randomized double-blind controlled trial | Otolaryngology - Head and Neck Surgery (United States). 2019; 161(5):807-813. | Excluded | Based on title and abstract |
| 1030 | Wang Y, 2021 | Dexmedetomidine-soaked nasal packing can reduce pain and improve sleep quality after nasal endoscopic surgery: a double-blind, randomized, controlled clinical trial | Drug design, development and therapy. 2020; 14:1083‐1093. | Excluded | Based on title and abstract |
| 1031 | Wang Y, 2021 | Effects of three different doses of dexmedetomidine and ropivacaine on analgesia and the stress response in hypospadias surgery: A randomized trial | Sleep Breath. 2021; 25(4):2045-2052. | Excluded | Based on title and abstract |
| 1032 | Wang Y, 2023 | Clinical observation of dexmedetomidine nasal spray in the treatment of sleep disorders on the first night after undergoing maxillofacial surgery: a single-center double-blind randomized controlled study | Frontiers in Pharmacology. 2021; 12. | Excluded | Based on title and abstract |
| 1033 | Wang YH, 2021 | Application of remifentanil in analgesia and sedation of mechanically ventilated patients in intensive care unit | Indian Journal of Pharmaceutical Sciences. 2021; 83:178-183. | Excluded | Based on title and abstract |
| 1034 | Wang Z, 2021 | Anesthesia, sedation, and unplanned extubation of tracheal intubation in children with severe pneumonia | Evidence-based Complementary and Alternative Medicine. 2021; 2021. | Excluded | Based on title and abstract |
| 1035 | Wang Z, 2021 | Emergence delirium in a 29-year-old man following an uneventful appendectomy | Case Reports in Medicine. 2021; 2021:1338823. | Excluded | Based on title and abstract |
| 1036 | Wang Z, 2021 | The value of combined application of oxycodone hydrochloride injection and dexmedetomidine in anesthesia for LC for patients with gallbladder lesions | Journal of Healthcare Engineering. 2021; 2021:1290650. | Excluded | Based on title and abstract |
| 1037 | Watson PL, 2012 | Delirium: Is sleep important? | Best Practice and Research: Clinical Anaesthesiology. 2012; 26(3):355-366. | Excluded | Based on title and abstract |
| 1038 | Waxman JA, 2020 | Assessment and management of postoperative pain associated with sleep apnea surgery | Otolaryngologic Clinics of North America. 2020; 53(5):765-777. | Excluded | Based on title and abstract |
| 1039 | Weant KA, 2010 | Pharmacologic options for reducing the shivering response to therapeutic hypothermia | Pharmacotherapy. 2010; 30(8):830-841. | Excluded | Based on title and abstract |
| 1040 | Weerink MAS, 2017 | Clinical pharmacokinetics and pharmacodynamics of dexmedetomidine | Clinical Pharmacokinetics. 2017; 56(8):893-913. | Excluded | Based on title and abstract |
| 1041 | Wei W, 2022 | Effects of subanaesthetic S-ketamine on postoperative delirium and cognitive function in elderly patients undergoing non-cardiac thoracic surgery: a protocol for a randomised, double-blinded, placebo-controlled and positive-controlled, non-inferiority trial (SKED trial) | BMJ Open. 2022; 12(8):e061535. | Excluded | Based on title and abstract |
| 1042 | Weinhouse GL, 2006 | Sleep in the critically ill patient | Sleep. 2006; 29(5):707-716. | Excluded | Based on title and abstract |
| 1043 | Weinhouse GL, 2011 | Sedation and sleep disturbances in the ICU | Anesthesiology Clinics. 2011; 29(4):675-685. | Excluded | Based on title and abstract |
| 1044 | Weinhouse GL, 2014 | Delirium and sleep disturbances in the intensive care unit: Can we do better? | Current Opinion in Anaesthesiology. 2014; 27(4):403-408. | Excluded | Based on title and abstract |
| 1045 | Wellisch OM, 2012 | A prospective randomized controlled trial comparing the efficacy and safety of dexmedetomidine versus propofol tiva in conjunction with regional block for shoulder surgery in beach chair sitting position | Regional anesthesia and pain medicine. 2012; 37(6). | Excluded | Based on title and abstract |
| 1046 | Wenjie X, 2020 | A comparative study on clinical effects of dexmedetomidine and midazolam on patients with severe coronavirus disease 2019 on non-invasive ventilation | Zhonghua Wei Zhong Bing Ji Jiu Yi Xue. 2020; 32(6):677-680. | Excluded | Based on title and abstract |
| 1047 | Whalen LD, 2014 | Long-term dexmedetomidine use and safety profile among critically Ill children and neonates | Pediatric Critical Care Medicine. 2014; 15(8):706-714. | Excluded | Based on title and abstract |
| 1048 | Whelan KT, 2015 | Pediatric withdrawal identification and management | Journal of Pediatric Intensive Care. 2015; 4(2):73-78. | Excluded | Based on title and abstract |
| 1049 | White PF, 2005 | Role of non-opioid analgesic techniques in the management of pain after ambulatory surgery | Anesthesia and Analgesia. 2005; 100(3 SUPPL.):105-109. | Excluded | Based on title and abstract |
| 1050 | White PF, 2005 | The changing role of non-opioid analgesic techniques in the management of postoperative pain | Anesthesia and Analgesia. 2005; 101(5 SUPPL.):S5. | Excluded | Based on title and abstract |
| 1051 | Wiegand T, 2013 | Use of buprenorphine and clonidine in treatment of combined heroin and xylazine withdrawal | Clinical Toxicology. 2013; 51(7):614-615. | Excluded | Based on title and abstract |
| 1052 | Wijayaratne S, 2021 | Opioid-free anaesthesia in bariatric surgery | Anaesthesia. 2021; 76(SUPPL 2):105. | Excluded | Based on title and abstract |
| 1053 | Wilcox LJ, 2017 | An updated review of pediatric drug-induced sleep endoscopy | Laryngoscope Investigative Otolaryngology. 2017; 2(6):423-431. | Excluded | Based on title and abstract |
| 1054 | Winch PD, 2016 | Learning from experience: Improving early tracheal extubation success after congenital cardiac surgery | Pediatric Critical Care Medicine. 2016; 17(7):630-637. | Excluded | Based on title and abstract |
| 1055 | Wiseman LK, 2022 | The effect of preoperative cannabis use on postoperative pain following gynaecologic oncology surgery | Journal of Obstetrics and Gynaecology Canada. 2022; 44(7):750-756. | Excluded | Based on title and abstract |
| 1056 | Withington DE, 2016 | Perchance to dream: sleeping peacefully, awaking smoothly | Pediatric Critical Care Medicine. 2016; 17(1):87-88. | Excluded | Based on title and abstract |
| 1057 | Wolfson A, 2007 | Postoperative analgesia for patients with obstructive sleep apnea syndrome | Seminars in Anesthesia, Perioperative Medicine and Pain. 2007; 26(2):103-109. | Excluded | Based on title and abstract |
| 1058 | Wong JK, 2016 | Nocturnal low-dose propofol infusion for the management of ICU delirium: A case series in nonintubated cardiac surgery patients | Journal of Cardiothoracic and Vascular Anesthesia. 2016; 30(5):1340-1343. | Excluded | Based on title and abstract |
| 1059 | Wong JWM, 2017 | High-flow nasal oxygen in patient with obstructive sleep apnea undergoing awake craniotomy: A case report | A and A Case Reports. 2017; 9(12):353-356. | Excluded | Based on title and abstract |
| 1060 | Wong SJ, 2021 | Patterns of obstruction on DISE in adults with obstructive sleep apnea change with BMI | Laryngoscope. 2021; 131(1):224-229. | Excluded | Based on title and abstract |
| 1061 | Wu J, 2019 | Different effects of oxycodone and remifentanil in patients undergoing ultrasound-guided percutaneous radiofrequency ablation of hepatic cancer: A randomized trial | Front Pharmacol. 2023; 14:1223746. | Excluded | Based on title and abstract |
| 1062 | Wu J, 2023 | Intranasal dexmedetomidine improves postoperative sleep quality in older patients with chronic insomnia: a randomized double-blind controlled trial | Drug Design, Development and Therapy. 2019; 13:365-372. | Excluded | Based on title and abstract |
| 1063 | Wu L, 2019 | Effect of ultrasound-guided peripheral nerve blocks of the abdominal wall on pain relief after laparoscopic cholecystectomy | J Pain Res. 2019; 12:1433-1439. | Excluded | Based on title and abstract |
| 1064 | Wu W, 2021 | Dexmedetomidine on postoperative cognitive function in sleep-deprived rats | International Journal of Clinical and Experimental Medicine. 2021; 14(4):1712-1719. | Excluded | Based on title and abstract |
| 1065 | Xiao Q, 2022 | Measuring airflow velocity in the upper airway in pediatric patients with obstructive sleep apnea via phase contrast MRI of hyperpolarized xenon | American Journal of Respiratory and Critical Care Medicine. 2022; 205(1). | Excluded | Based on title and abstract |
| 1066 | Xiao Q, 2022 | Obstructive apnea hypopnea index of children with persistent obstructive sleep apnea correlates significantly with airway neuromuscular activation | American Journal of Respiratory and Critical Care Medicine. 2022; 205(1). | Excluded | Based on title and abstract |
| 1067 | Xing F, 2018 | Intrathecal drug delivery and spinal cord stimulation for the treatment of cancer pain | Current Pain and Headache Reports. 2018; 22(2). | Excluded | Based on title and abstract |
| 1068 | Xing J, 2021 | Comparison of the analgesic effects of intravenous infusion of dexmedetomidine versus bilateral superficial cervical plexus block after thyroidectomy: A randomized controlled trial | Clinical journal of pain. 2021; 37(8):623‐628. | Excluded | Based on title and abstract |
| 1069 | Xu H, 2015 | The preliminary study of the origin characters of snore in simple snorers | Lin Chung Er Bi Yan Hou Tou Jing Wai Ke Za Zhi. 2015; 29(11):977-979, 983. | Excluded | Based on title and abstract |
| 1070 | Xu HJ, 2015 | Investigation of the source of snoring sound by drug-induced sleep nasendoscopy | ORL. 2015; 77(6):359-365. | Excluded | Based on title and abstract |
| 1071 | Xu J, 2015 | Comparison of dexmedetomidine versus propofol for sedation after uvulopalatopharyngoplasty | Medical science monitor. 2015; 21:2125‐2133. | Excluded | Based on title and abstract |
| 1072 | Xu JK, 2019 | Polysomnographic comparation between dexmedetomidine-induced sleep and natural sleep | Chinese Journal of Otorhinolaryngology Head and Neck Surgery. 2019; 54(6):405-409. | Excluded | Based on title and abstract |
| 1073 | Xu L, 2018 | Efficacy of US-guided transversus abdominis plane block and rectus sheath block with ropivacaine and dexmedetomidine in elderly high-risk patients | Minerva anestesiologica. 2018; 84(1):18‐24. | Excluded | Based on title and abstract |
| 1074 | Xu WY, 2021 | Half effective bolus dose of dexmedetomidine of children derived from the up-and-down experimental data based on the analysis of nonlinear mixed effects model with allometric equation | Journal of Shanghai Jiaotong University (Medical Science). 2021; 41(10):1313-1317. | Excluded | Based on title and abstract |
| 1075 | Yagar S, 2011 | The role of the ADRA2A C1291G genetic polymorphism in response to dexmedetomidine on patients undergoing coronary artery surgery | Molecular Biology Reports. 2011; 38(5):3383-3389. | Excluded | Based on title and abstract |
| 1076 | Yanan J, 2022 | A comparative study on sedation efficacy between general and regional anesthesia with dexmedetomidine in patients under maxillofacial surgery | Curr Drug Metab. 2022; 23(11):920-927. | Excluded | Based on title and abstract |
| 1077 | Yang H, 2022 | Preventing nausea and vomiting after gynecological laparoscopic surgery by patient-controlled intravenous analgesia with a naloxone admixture: A randomized controlled trial | Medicine (United States). 2022; 101(29):E29584. | Excluded | Based on title and abstract |
| 1078 | Yang HC, 2018 | The efficacy of drug induced sleep endoscopy using multimodality monitoring system | PLoS One. 2018; 13(12):e0209775. | Excluded | Based on title and abstract |
| 1079 | Yang J, 2022 | A Feasibility study on caffeine citrate to facilitate intraoperative brain mapping and cognitive testing during awake craniotomies | Journal of Neurosurgical Anesthesiology. 2022; 34(4):452-453. | Excluded | Based on title and abstract |
| 1080 | Yang X, 2022 | Impacts of ultrasound-guided nerve block combined with general anesthesia with laryngeal mask on the patients with lower extremity fractures | J Environ Public Health. 2022; 2022:3603949. | Excluded | Based on title and abstract |
| 1081 | Ye J, 2014 | Effects of obstruction length and height on predicting velopharyngeal surgery outcome in obstructive sleep apnea | Otolaryngology - Head and Neck Surgery (United States). 2014; 151(1):P133. | Excluded | Based on title and abstract |
| 1082 | Ye J, 2014 | Two new parameters: Obstruction length and height in predicting outcome of upper airway surgery in obstructive sleep apnea patients | Journal of Sleep Research. 2014; 23:117-118. | Excluded | Based on title and abstract |
| 1083 | Yi HW, 2022 | Dexmedetomidine combined with ropivacaine for erector spinae plane block after posterior lumbar spine surgery: a randomized controlled trial | BMC Musculoskeletal Disorders. 2022; 23(1). | Excluded | Based on title and abstract |
| 1084 | Yli HA, 2012 | Monitoring sedation during regional anaesthesia | Regional Anesthesia and Pain Medicine. 2012; 37(5):E171-E172. | Excluded | Based on title and abstract |
| 1085 | Yoon BW, 2016 | A comparison of dexmedetomidine versus propofol during drug-induced sleep endoscopy in sleep apnea patients | Laryngoscope. 2016; 126(3):763-767. | Excluded | Based on title and abstract |
| 1086 | Young ET, 2005 | Dexmedetomidine sedation in a pediatric cardiac patient scheduled for MRI | Can J Anaesth. 2005; 52(7):730-732. | Excluded | Based on title and abstract |
| 1087 | Yu JM, 2016 | The analgesic effect of ropivacaine combined with dexmedetomidine for incision infiltration after laparoscopic cholecystectomy | J Pain Res. 2023; 16:3625-3632. | Excluded | Based on title and abstract |
| 1088 | Yu JM, 2023 | Opioid-free anesthesia for pain relief after laparoscopic cholecystectomy: A prospective randomized controlled trial | Surg Laparosc Endosc Percutan Tech. 2016; 26(6):449-454. | Excluded | Based on title and abstract |
| 1089 | Yu PK, 2022 | Evaluation of upper airway stimulation for adolescents with down syndrome and obstructive sleep apnea | JAMA Otolaryngology - Head and Neck Surgery. 2022; 148(6):522-528. | Excluded | Based on title and abstract |
| 1090 | Yu SB, 2012 | Dexmedetomidine sedation in ICU | Korean Journal of Anesthesiology. 2012; 62(5):405-411. | Excluded | Based on title and abstract |
| 1091 | Yu X, 2018 | Sleep and sedative states induced by targeting the histamine and noradrenergic systems | Frontiers in Neural Circuits. 2018; 12. | Excluded | Based on title and abstract |
| 1092 | Yun D, 2022 | Fatal rhabdomyolysis and disseminated intravascular coagulation after total knee arthroplasty under spinal anesthesia: A case report | World Journal of Clinical Cases. 2022; 10(4):1349-1356. | Excluded | Based on title and abstract |
| 1093 | Zahr N, 2016 | Striatal BOLD responses to VTA optogenetic stimulation during inhibition of locus coeruleus noradrenergic neurons | Neuropsychopharmacology. 2016; 41:S616-S617. | Excluded | Based on title and abstract |
| 1094 | Zakaria S, 2018 | Editor's choice-The cardiovascular implications of sedatives in the cardiac intensive care unit | European Heart Journal: Acute Cardiovascular Care. 2018; 7(7):671-683. | Excluded | Based on title and abstract |
| 1095 | Zecharia AY, 2009 | General anesthesia and ascending arousal pathways | Anesthesiology. 2009; 111(4):695-696. | Excluded | Based on title and abstract |
| 1096 | Zen L, 2020 | Home intranasal dexmedetomidine for refractory dystonia in pediatric palliative care | Journal of Pain and Symptom Management. 2020; 59(6):e3-e5. | Excluded | Based on title and abstract |
| 1097 | Zen L, 2020 | Home use of intranasal dexmedetomidine in a child with an intractable sleep disorder | Journal of Pediatric Pharmacology and Therapeutics. 2020; 25(4):332-335. | Excluded | Based on title and abstract |
| 1098 | Zeng XZ, 2014 | Low-dose epidural dexmedetomidine improves thoracic epidural anaesthesia for nephrectomy | Anaesthesia and intensive care. 2014; 42(2):185‐190. | Excluded | Based on title and abstract |
| 1099 | Zhan J, 2023 | Effect of transversus abdominis plane block combined with low-dose dexmedetomidine on elderly patients undergoing laparoscopic colectomy | Wideochir Inne Tech Maloinwazyjne. 2023; 18(3):524-532. | Excluded | Based on title and abstract |
| 1100 | Zhan W, 2020 | Analgesic effect and safety of postoperative low-dose ketamine/midazolam combination vis-a-vis dexmedetomidine in non-cardiac surgery | Tropical Journal of Pharmaceutical Research. 2020; 19(11):2453-2460. | Excluded | Based on title and abstract |
| 1101 | Zhang B, 2018 | The opioid-sparing effect of perioperative dexmedetomidine combined with Oxycodone infusion during open hepatectomy: a randomized controlled trial | Front pharmacol. 2018; 8:940. | Excluded | Based on title and abstract |
| 1102 | Zhang JR, 2023 | Effect of ropivacaine combined with dexmedetomidine for serratus anterior plane block plus patient-controlled intravenous analgesia on postoperative recovery quality of patients undergoing thoracoscopic radical resection of lung cancer | J Sichuan Uni(Med Sci Edit) 2023; 54(1):155-160. | Excluded | Based on title and abstract |
| 1103 | Zhang P, 2015 | The Role of obstruction length and height in predicting outcome of velopharyngeal surgery | Otolaryngol Head Neck Surg. 2015; 153(1):144-149. | Excluded | Based on title and abstract |
| 1104 | Zhang SJ, 2021 | α2-adrenergic receptor agonist, an attractive but underused ERAS component in improving fast-track recovery and surgical outcomes | AANA Journal. 2021; 89(6):529-537. | Excluded | Based on title and abstract |
| 1105 | Zhang X, 2019 | The value of drug-induced sleep computed tomography in diagnosis of obstructive sleep apnea syndrome: a pilot study | Acta Otolaryngol. 2019; 139(10):895-901. | Excluded | Based on title and abstract |
| 1106 | Zhang X, 2022 | Dexmedetomidine improves non-rapid eye movement stage 2 sleep in children in the intensive care unit on the first night after laparoscopic surgery | Front Pediatr. 2022; 10:871809. | Excluded | Based on title and abstract |
| 1107 | Zhang XH, 2021 | Combination of dexmedetomidine and butorphanol optimized sedation in drug-induced sleep endoscopy: A randomized, double-blind trial | Curr Med Sci. 2021; 41(6):1247-1251. | Excluded | Based on title and abstract |
| 1108 | Zhang XK, 2016 | Evaluation of dexmedetomidine in combination with sufentanil or butorphanol for postoperative analgesia in patients undergoing laparoscopic resection of gastrointestinal tumors: A quasi-experimental trial | Medicine (United States). 2016; 95(50):e5604. | Excluded | Based on title and abstract |
| 1109 | Zhang Y, 2023 | Mini-dose esketamine-dexmedetomidine combination to supplement analgesia for patients after scoliosis correction surgery: a double-blind randomised trial | Br J Anaesth. 2023; 131(2):385-396. | Excluded | Based on title and abstract |
| 1110 | Zhang Z, 2015 | Neuronal ensembles sufficient for recovery sleep and the sedative actions of α 2 adrenergic agonists | Nature Neuroscience. 2015; 18(4):553-561. | Excluded | Based on title and abstract |
| 1111 | Zhao C, 2020 | Insights into friedman stage II and III OSA patients through drug-induced sleep endoscopy | Journal of Thoracic Disease. 2020; 12(7):3663-3672. | Excluded | Based on title and abstract |
| 1112 | Zhao C, 2022 | Upper airway collapse characteristics in adult patients with OSA and previous tonsillectomy | Sleep and Breathing. 2022; 26(2):717-723. | Excluded | Based on title and abstract |
| 1113 | Zhao LL, 2018 | A comparative study on efficacy and safety of propofol versus dexmedetomidine in sleep apnea patients undergoing drug-induced sleep endoscopy: A CONSORT-prospective, randomized, controlled clinical trial | Biomed Res Int. 2018; 2018:8696510. | Excluded | Based on title and abstract |
| 1114 | Zhao W, 2020 | The effect and optimal dosage of dexmedetomidine plus sufentanil for postoperative analgesia in elderly patients with postoperative delirium and early postoperative cognitive dysfunction: A single-center, prospective, randomized, double-blind, controlled trial | Frontiers in Neuroscience. 2020; 14. | Excluded | Based on title and abstract |
| 1115 | Zhaoyan Y, 2019 | A retrospective analysis of 124 patients with obstructive sleep apnea by drug-induced sleep computed tomography | Sleep. 2019; 42:A181. | Excluded | Based on title and abstract |
| 1116 | Zhengling T, 2019 | Analgesia efficacy of dexmedetomidine combined with ropivacaine for adductor canal block after total knee arthroplasty | Chinese journal of tissue engineering research. 2019; 23(24):3798‐3804. | Excluded | Based on title and abstract |
| 1117 | Zhou J, 2021 | Effect of patient-controlled intravenous analgesia combined with flurbiprofen axetil and dezocine on postoperative analgesia for lobectomy (EPIC-FAD): a trial protocol | Trials. 2021; 22(1). | Excluded | Based on title and abstract |
| 1118 | Zhou P, 2014 | Research on the patterns of upper airway obstructive levels by drug-induced sleep endoscopy | Zhonghua Er Bi Yan Hou Tou Jing Wai Ke Za Zhi. 2014; 49(1):58-61. | Excluded | Based on title and abstract |
| 1119 | Zhou Y, 2022 | Anesthesia management of morbid obesity and ankylosing spondylitis with a difficult airway: a case report | American Journal of Translational Research. 2022; 14(7):4860-4863. | Excluded | Based on title and abstract |
| 1120 | Zhuang PJ, 2011 | Postoperative respiratory and analgesic effects of dexmedetomidine or morphine for adenotonsillectomy in children with obstructive sleep apnoea | Anaesthesia. 2011; 66(11):989-993. | Excluded | Based on title and abstract |
| 1121 | Zoremba N, 2019 | Acute confusional states in hospital | Dtsch Arztebl Int. 2019; 116(7):101-106. | Excluded | Based on title and abstract |
| 1122 | Zuppa AF, 2017 | Sedation analgesia and neuromuscular blockade in pediatric critical care: overview and current landscape | Pediatric Clinics of North America. 2017; 64(5):1103-1116. | Excluded | Based on title and abstract |
| 1123 | Zutler M, 2011 | Opioids, sleep, and sleep-disordered breathing | Current Pharmaceutical Design. 2011; 17(15):1443-1449. | Excluded | Based on title and abstract |
