# Supplementary material for: Effect of perioperative dexmedetomidine on sleep quality in adult patients after noncardiac surgery: A systematic review and meta-analysis of randomized trials
Source: PLoS One. 2024 Dec 5;19(12):e0314814. doi: 10.1371/journal.pone.0314814 (PMC11620464; doi:10.1371/journal.pone.0314814)
Supplement: S2 Table — (DOCX) [file pone.0314814.s005.docx]

**S2 Table.** Summary of details of the included studies.

| Author, year | Data extractors | Date of extraction | Included | Subjective sleep quality (D/C) | PSG (D/C) | NRS of pain at 24 h (D/C) | Opioid consumption (D/C) | Delirium (D/C) | Bradycardia (D/C) | Hypotension (D/C) |
| --- | --- | --- | --- | --- | --- | --- | --- | --- | --- | --- |
| Chen C, 2016^[23]^ | Wang L, Liang XQ | Dec 12, 2022 | Yes | D/C=30/30; NRS (0-10; higher score worse)  POD1: 3.5±0.3/5.8±0.4 | --- | 2.8±0.3/3.2±0.3 | suf (μg): 103±5/122±9 | --- | --- | --- |
| Chen Z, 2017^[24]^ | Wang L, Liang XQ | Dec 25, 2022 | Yes | D/C=30/29; NRS (0-10; higher score better)  POD1: 7 (6-8)/3 (2-5)  POD2: 8 (7-8)/5 (4-6) | D/C=30/29  SEI (%): 65±5/42±5  N1 (%): 16±3/39±4  N2 (%): 68±6/47±5  N3 (%): 10±2/9±2  REM (%): 5±2/5±2  AI (times/h): 7±2/12±2 | 1 (0-2)/3 (2-5) | suf (μg):  68±7/88±8 | --- | --- | --- |
| Dong YS, 2024^[25]^ | Wang L, Liang XQ | Mar 16, 2024 | Yes | S/P/DS/DP=47/47/47/47; AIS (0-24; higher score worse)  POD1: 5 (3-6)/5 (4-6)/6 (3-7)/5 (4-6)  POD3: 4 (3-6)/5 (3-7)/5 (4-6)/4 (3-6)  POD7: 5 (4-6)/5 (5-7)/6 (5-6)/5 (4-6)  POD30: 5 (3-6)/5 (3-6)/5 (3-6)/2 (3-5) | --- | --- | --- | --- | --- | --- |
| Huang J，2023^[26]^ | Wang L, Liang XQ | Mar 16, 2024 | Yes | D/C=20/20; NRS (0-100; higher score better)  POD1: 73.5±4.2/56.8±4.2 | D/C=17/18  SEI (%): 78±11/78±14  N1 (%): 4 (2-7)/5 (3-10)  N2 (%): 77±8/75±7  N3 (%): 0 (0-4)/0 (0-0)  REM (%): 16±6/17±7  AI (times/h): 10±7/15±10 | 1 (1-1)/2 (2-2) | suf (μg):  37±1/42±1 | 0 (0%)/0 (0%) | --- | --- |
| Jiang Z, 2018^[28]^ | Wang L, Liang XQ | Dec 21, 2022 | Yes | D1/D2/C=33/33/32; NRS (0-10; higher score better)  POD1: 6 (5-7)/7 (6-7)/4 (3-4)  POD2: 7 (7-8)/8 (7-8)/5 (4-5) | D1/D2/C=33/33/32  SEI (%): 63 (55-70)/68 (60-72)/42 (35-42)  N1 (%): 19 (16-20)/18 (17-21)/33 (31-37)  N2 (%): 57 (57-60)/63 (60-66)/35 (35-40)  N3 (%): 11 (9-13)/9 (6-11)/12 (10-16)  REM (%): 12 (10-13)/9 (7-12)/15 (12-17)  AI (times/h): 7 (6-8)/6 (6-7)/11 (9-12) | --- | --- | --- | 9 (14%)/4 (13%) | 10 (15%)/2 (6%) |
| Kang RA, 2019^[29]^ | Wang L, Liang XQ | Dec 25, 2022 | Yes | D/C=22/22; Likert scale (1-5; higher score better)  POD1: 5 (3-5)/3 (1-3) | --- | --- | mor (mg):  19 (15-28)/27 (20-37) | --- | --- | --- |
| Li HJ, 2018^[30]^ | Wang L, Liang XQ | Dec 12, 2022 | Yes | D/C=28/29; NRS (0-10; higher score better)  POD1: 7 (4-9)/5 (3-6)  POD2: 8 (7-9)/7 (6-7)  POD3: 8 (8-9)/8 (7-9) | --- | 2 (1-4)/4 (3-5) | mor (mg):  39 (37- 41)/49 (46-50) | 0 (0%)/2 (7%) | 0 (0%)/0 (0%) | 1 (4%)/2 (7%) |
| Li S, 2023^[31]^ | Wang L, Liang XQ | Mar 16, 2024 | Yes | D/C=130/130; RCSQ (0-100; higher score better)  POD1: 72 (60-80)/60 (50-70)  POD2: 78 (70-84)/70 (62-78)  POD3: 82 (74-88)/76 (70-82) | --- | 2 (2-3)/3 (2-4) | --- | 28 (22%)/60 (46%) | 5 (4%)/3 (2%) | 12 (9%)/9 (7%) |
| Liu T, 2022^[32]^ | Wang L, Liang XQ | Dec 22, 2022 | Yes | D/C=60/60; RCSQ (0-100; higher score better)  POD1: 61.8±6.1/54.3±6.9 | --- | 1.2±0.4/1.5±0.5 | --- | --- | 8 (13%)/4 (7%) | 3 (5%)/1 (2%) |
| Liu X, 2020^[33]^ | Wang L, Liang XQ | Dec 11, 2022 | Yes | D/C=38/37; PSQI (0-21; higher score worse)  1 month after surgery:  6.0±1.5/7.8±1.5 | --- | 5.1±0.1/6.3±0.3 | --- | --- | --- | --- |
| Lu Y, 2021^[34]^ | Wang L, Liang XQ | Dec 21, 2022 | Yes | D/C=344/331; NRS (0-10; higher score worse)  POD1: 2 (1-4)/2 (1-4)  POD2: 1 (0-3)/1 (1-2)  POD3: 1 (1-3)/1 (1-2)  POD7: 0 (0-1)/0 (0-1) | --- | 3 (2-3)/3 (2-3) | --- | 41 (12%)/43 (13%) | --- | --- |
| Mao Y, 2020^[35]^ | Wang L, Liang XQ | Dec 26, 2022 | Yes | D/C=29/29; St.Mary’s Hospital Sleep Questionire (1-6; higher score better)  POD2: 5 (4-5)/4 (4-4) | --- | 2 (1-3)/2 (2-3) | suf (μg):  1±4/1±3 | 0 (0)/1 (3%) | --- | --- |
| Qin M, 2017^[36]^ | Wang L, Liang XQ | Dec 13, 2022 | Yes | --- | --- | 1 (1-1)/ 2 (2-3) | suf (μg):  38±2/48±5 | --- | --- | --- |
| Shi H, 2020^[37]^ | Wang L, Liang XQ | Dec 12, 2022 | Yes | D/C=53/53; NRS (0-10; higher score worse)  POD1: 2.0±0.6/2.6±0.7 | --- | 0±0.1/0.1±0.3 | --- | 4 (8%)/6 (11%) | 26 (49%)/13 (25%) | 6 (11%)/4 (8%) |
| Shi J, 2022^[38]^ | Wang L, Liang XQ | Dec 25, 2022 | Yes | D/C=142/143; PSQI (0-21; higher score worse)  POD1: 12.4±2.7/13.0±2.9  POD2: 8.6±2.0/9.7±2.1  POD7: 4.7±1.3/6.4±1.6 | --- | --- | --- | --- | 5 (4%)/9 (6%) | 2 (1%)/11 (8%) |
| Sui X, 2022^[39]^ | Wang L, Liang XQ | Dec 28, 2022 | Yes | D1/D2/C=70/70/70; AIS (0-24; higher score worse)  POD1: 4.0±2.7/3.2±2.7/6.5±4.6  POD2: 3.3±2.2/3.0±2.5/4.3±2.9  POD3: 3.3±1.9/3.3±3.2/5.2±3.7  POD7: 2.2±1.6/1.7±2.1/3.5±2.9 | --- | 1 (0-3)/1 (0-4)/1 (0-4) | suf (μg): 93±17/96±16/100±20 | --- | --- | --- |
| Sun Y, 2019^[40]^ | Wang L, Liang XQ | Dec 25, 2022 | Yes | D/C=281/276; RCSQ (0-100; higher score better)  POD1: 64.6±16.5/53.5±17.0  POD2: 63.9±19.0/54.8±18.2  POD3: 68.4±16.3/58.5±17.9 | --- | 1 (1-2)/2 (1-3) | suf (μg):  40 (30-50)/40 (30-50) | 33 (12%)/38 (14%) | 26 (9%)/14 (5%) | 4 (1%)/2 (1%) |
| Sun YM, 2022^[41]^ | Wang L, Liang XQ | Dec 22, 2022 | Yes | D/C=33/35; RCSQ (0-100; higher score better)  POD1: 61 (27-79)/52 (20-66);  PSQI (0-21; higher score worse)  POD30: 8 (5-12)/7 (4-11) | D/C=33/35  SEI (%): 48 (29-64)/34 (23-58)  N1 (%): 6 (1-12)/12 (2-27)  N2 (%): 90 (71-95)/85 (68-96)  N3 (%): 0 (0-7)/0 (0-0)  REM: 0 (0-0)/0 (0-0)  AI (times/h): 3 (2-5)/4 (3-7) | --- | --- | 6 (15%)/5 (13%) | 4 (10%)/0 (0%) | 1 (3%)/2 (5%) |
| Su X, 2016^[14]^ | Wang L, Liang XQ | Dec 11, 2022 | Yes | D/C=350/350; NRS (0-10; higher score worse)  POD1: 2 (0-4)/4 (2-6)  POD2: 2 (1-5)/4 (2-6)  POD3: 2 (0-4)/4 (2-5) | --- | 1 (0-2)/1 (0-3) | mor (mg):  4 (2-7)/4 (2-6) | 32 (9%)/79 (23%) | 59 (17%)/46 (13%) | 114 (33%)/92 (26%) |
| Tan W, 2016^[17]^ | Wang L, Liang XQ | Dec 10, 2022 | Yes | --- | D/C=32/32  Bis SEI (%): 15±2/29±3 | --- | --- | --- | --- | --- |
| Tan W,  2016 (2)^[42]^ | Wang L, Liang XQ | Dec 10, 2022 | Yes | --- | D/C=55/53  Bis SEI (%):  14±11/14±10 | 4 (2-5)/5 (4-5) | suf (μg): 38±11/45±7 | --- | --- | --- |
| Ting H, 2019^[27]^ | Wang L, Liang XQ | Dec 26, 2022 | Yes | D/C=173/173; PSQI (0-21; higher score worse)  POD1: 2 (1-4)/5 (4-6)  POD2: 2 (0-3)/4 (3-5)  POD3: 3 (2-4)/3 (2-4) | --- | 3 (1-4)/4 (2-5) | --- | --- | --- | --- |
| Wu XH, 2016^[15]^ | Wang L, Liang XQ | Dec 26, 2022 | Yes | D/C=38/38; NRS (0-10; higher score worse)  POD1: 2 (1-4)/4 (2-8)  POD2: 3 (2-8)/4 (1-6)  POD3: 3 (2-6)/4 (2-6) | D/C=31/30  N1 (%): 56 (13-83)/84 (28-99)  N2 (%): 44 (17-80)/16 (1-63)  N3 (%): 0 (0-1)/0 (0-0)  REM (%): 0 (0-0)/0 (0-0) | 0 (0-0)/0 (0-1) | mor (mg):  0 (0-0)/0 (0-2) | --- | 12 (32%)/6 (16%) | 15 (40%)/5 (13%) |
| Wu Y, 2022^[43]^ | Wang L, Liang XQ | Dec 09, 2022 | Yes | --- | D/C=48/48  SEI (%): 67±3/65±4  AI (times/h): 7±1/8±1  REM (%): 13±1/12±2 | 2.6±0.2/2.8±0.2 | --- | --- | --- | --- |
| Xu S，2023^[44]^ | Wang L, Liang XQ | Mar 16, 2024 | Yes | C/L/D/LD=40/40/40/40; NRS (0-10; higher score better)  POD1: 3 (2-3)/3 (2-4)/3 (3-4)/5 (4-5) | --- | 2 (1-2)/1 (1-2)/1 (1-2)/1 (1-2) | --- | --- | 8 (20%)/10 (25%)/28 (70%)/30 (75%) | 6 (15%)/7 (18%)/9 (23%)/10 (25%) |
| Yang X, 2015^[45]^ | Wang L, Liang XQ | Dec 20, 2022 | Yes | D/C=39/40; NRS (0-10; higher score better)  POD1: 5 (3-7)/5 (3-8) | --- | 4 (2-5)/4 (3-5) | suf (μg):  5±6/7±7 | --- | 1 (3%)/0 (0%) | 0 (0%)/1 (3%) |
| Yu HY, 2019^[46]^ | Wang L, Liang XQ | Dec 11, 2022 | Yes | D/C=281/276; ISI (0-28; higher score worse)  POD2: 5.5±3.2/7.7±2.8  POD7: 4.1±2.5/3.8±2.0  POD42: 4.2±2.6/3.9±2.2 | --- | 1.8±0.8/2.3±0.6 | --- | --- | --- | --- |
| Yu Y, 2023^[47]^ | Wang L, Liang XQ | Mar 16, 2024 | Yes | D/C=156/154; NRS (0-10; higher score worse)  POD1 2 (1-3)/3 (2-5)  POD2 2 (1-3)/2 (2-4)  POD3 2 (1-2)/2 (2-4) | --- | 2.9±1.4/3.2±1.3 | --- | 20 (13%)/23 (15%) | --- | --- |
| Zhang ZF, 2022^[48]^ | Wang L, Liang XQ | Mar 21, 2023 | Yes | D/C=58/59; NRS (0-100 higher score worse)  POD1: 45.0±18.1/47.5±19.4  POD2: 33.4±16.0/36.5±19.3  POD3: 28.0±16.0/28.6±16.4 | D/C=44/41  SEI (%): 53 (43-64)/37 (12-57)  N1 (%): 24 (13-35)/33 (17-58)  N2 (%): 74 (64-86)/66 (39-81)  N3 (%): 0 (0-0)/0 (0-0)  REM (%): 0 (0-0)/0 (0-0)  AI (times/h): 5 (3-9)/6 (4-13) | 2 (1-3)/1 (1-3) | mor (mg):  37 (34-41)/37 (34-41) | --- | 0 (0%)/0 (0%) | 2 (3%)/1 (2%) |

Data are e mean ± SD, median (interquartile range), or n (%), as appropriate.

D/C, dexmedetomidine/control; PSG, polysomnography; NRS, numerical rating scale; NRS of pain, a 0-10 scale where 0=no pain and 10=the worst pain; POD, postoperative day; suf, sufentanil; SEI, sleep efficiency index; N1/N2/N3, stage 1/2/3 of non-rapid eye movement sleep; REM, rapid eye movement sleep; AI, arousal index; S/P/DS/DP, sevoflurane/propofol/dexmedetomidine and sevoflurane/dexmedetomidine and propofol; D1/D2/C, dexmedetomidine group 1/dexmedetomidine group 2/control group; AIS, Athens insomnia scale; mor, morphine; RCSQ, Richards-Campbell sleep questionnaire; PSQI, Pittsburgh sleep quality index; BIS, bispectral index; C/L/D/LD, control/lidocaine/dexmedetomidine/lidocaine and dexmedetomidine; ISI, Insomnia severity index.

“---” indicates no-related data.
