# Supplementary material for: Effect of perioperative dexmedetomidine on sleep quality in adult patients after noncardiac surgery: A systematic review and meta-analysis of randomized trials
Source: PLoS One. 2024 Dec 5;19(12):e0314814. doi: 10.1371/journal.pone.0314814 (PMC11620464; doi:10.1371/journal.pone.0314814)
Supplement: S3 Table — (DOCX) [file pone.0314814.s006.docx]

**S3 Table.** Risk of bias of the included studies.

| Author, year | Randomization process | Deviations from intended interventions | Missing outcome data | Measurement of the outcome | Selection of the reported result | Overall |
| --- | --- | --- | --- | --- | --- | --- |
| Chen C, 2016^[23]^ | Some concerns | Low risk | Low risk | Low risk | Low risk | Some concerns |
| Chen Z, 2017^[24]^ | Some concerns | Low risk | Low risk | High risk | Some concerns | High risk |
| Dong YS, 2024^[25]^ | Low risk | Low risk | Low risk | Low risk | Some concerns | Some concerns |
| Huang J, 2023^[26]^ | Some concerns | Low risk | Low risk | Low risk | Low risk | Some concerns |
| Jiang Z, 2018^[28]^ | Some concerns | Low risk | Low risk | Low risk | Some concerns | Some concerns |
| Kang RA, 2019^[29]^ | Low risk | Low risk | Low risk | Some concerns | Some concerns | Some concerns |
| Li HJ, 2018^[30]^ | Low risk | Low risk | Low risk | Low risk | Some concerns | Some concerns |
| Li S, 2023^[31]^ | Low risk | Low risk | Low risk | Low risk | Low risk | Low risk |
| Liu T, 2022^[32]^ | Low risk | Low risk | Low risk | Low risk | Some concerns | Some concerns |
| Liu X, 2020^[33]^ | Some concerns | High risk | Low risk | Some concerns | Some concerns | High risk |
| Lu Y, 2021^[34]^ | Low risk | Some concerns | Low risk | Low risk | Some concerns | Some concerns |
| Mao Y, 2020^[35]^ | Low risk | Low risk | Low risk | Low risk | Some concerns | Some concerns |
| Qin M, 2017^[36]^ | Some concerns | Low risk | Low risk | Low risk | Low risk | Some concerns |
| Shi H, 2020^[37]^ | Some concerns | Low risk | Low risk | Low risk | Some concerns | Some concerns |
| Shi J, 2022^[38]^ | Some concerns | Low risk | Low risk | Some concerns | Some concerns | Some concerns |
| Sui X, 2022^[39]^ | Some concerns | Low risk | Low risk | Low risk | Some concerns | Some concerns |
| Sun Y, 2019^[40]^ | Low risk | Low risk | Low risk | Low risk | Low risk | Low risk |
| Sun YM, 2022^[41]^ | Low risk | Some concerns | Low risk | Low risk | Low risk | Some concerns |
| Su X, 2016^[14]^ | Low risk | Low risk | Low risk | Low risk | Low risk | Low risk |
| Tan W, 2016^[17]^ | Low risk | Some concerns | High risk | Low risk | Low risk | High risk |
| Tan W, 2016 (2)^[42]^ | Low risk | High risk | Low risk | Low risk | Low risk | High risk |
| Ting H, 2019^[27]^ | Some concerns | Some concerns | Low risk | Some concerns | Some concerns | Some concerns |
| Wu XH, 2016^[15]^ | Some concerns | Low risk | Low risk | Low risk | Low risk | Some concerns |
| Wu Y, 2022^[43]^ | Low risk | Some concerns | Low risk | Low risk | Some concerns | Some concerns |
| Xu S, 2023^[44]^ | Low risk | Low risk | Low risk | Low risk | Low risk | Low risk |
| Yang X, 2015^[45]^ | Some concerns | Some concerns | Low risk | Low risk | Low risk | Some concerns |
| Yu HY, 2019^[46]^ | High risk | Low risk | Low risk | Low risk | Low risk | High risk |
| Yu Y, 2023^[47]^ | Some concerns | Low risk | Low risk | Low risk | Some concerns | Some concerns |
| Zhang ZF, 2022^[48]^ | Low risk | Low risk | Low risk | Low risk | Low risk | Low risk |
