# Supplementary material for: Effect of perioperative dexmedetomidine on sleep quality in adult patients after noncardiac surgery: A systematic review and meta-analysis of randomized trials
Source: PLoS One. 2024 Dec 5;19(12):e0314814. doi: 10.1371/journal.pone.0314814 (PMC11620464; doi:10.1371/journal.pone.0314814)
Supplement: S4 Table — (DOCX) [file pone.0314814.s007.docx]

**S4 Table.** GRADE quality of evidence assessment for each outcome.

| **Outcome** | **Limitations** | **Inconsistency** | **Indirectness** | **Imprecision** | **Publication bias** | **N of participants** | **Conclusion** | **Quality of evidence** |
| --- | --- | --- | --- | --- | --- | --- | --- | --- |
| **Primary outcome** |  |  |  |  |  |  |  |  |
| Subjective sleep score on the 1^st^ night (point) ^[14,15,21-30,32,35-39,42,43,45,46]^ | Risk of bias ^a^ | Serious ^b^ | No | No | Strongly suspected ^c^ | 4611 | DEX superior to placebo | Very low quality (⊕OOO) |
| **Exploratory outcomes** |  |  |  |  |  |  |  |  |
| Polysomnographic parameters |  |  |  |  |  |  |  |  |
| Sleep efficiency index (SEI; %) ^[15,22,24,26,39,41,46]^ | No | Serious ^b^ | No | No | Strongly suspected ^c^ | 501 | DEX superior to placebo | Low quality (⊕⊕OO) |
| Arousal index (AI; times/h) ^[22,24,26,39,41,46]^ | No | Serious ^b^ | No | No | Strongly suspected ^c^ | 440 | DEX superior to placebo | Low quality (⊕⊕OO) |
| Stage 1 of non-REM sleep (N1; %) ^[15,22,24,26,39,46]^ | No | Serious ^b^ | No | No | Strongly suspected ^c^ | 405 | DEX superior to placebo | Low quality (⊕⊕OO) |
| Stage 2 of non-REM sleep (N2; %) ^[15,22,24,26,39,46]^ | No | Serious ^b^ | No | No | Strongly suspected ^c^ | 405 | DEX superior to placebo | Low quality (⊕⊕OO) |
| Stage 3 of non-REM sleep (N3; %) ^[22,26]^ | Small sample size ^d^ | Serious ^b^ | No | Serious ^e^ | Strongly suspected ^c^ | 156 | No difference | Very low quality (⊕OOO) |
| REM sleep (%) ^[22,24,26,39,41]^ | Small sample size ^d^ | Serious ^b^ | No | Serious ^e^ | Strongly suspected ^c^ | 355 | No difference | Very low quality (⊕OOO) |
| Subjective sleep score on the 2^nd^ night (point) ^[14,15,22,25,26,28,29,32,36-38,44-46]^ | Risk of bias ^a^ | Serious ^b^ | No | No | Strongly suspected ^c^ | 4291 | DEX superior to placebo | Very low quality (⊕OOO) |
| Subjective sleep score on the 3^rd^ night (point) ^[14,15,23,25,29,32,37,38,45,46]^ | Risk of bias ^a^ | Serious ^b^ | No | No | Undetected | 3423 | DEX superior to placebo | Low quality (⊕⊕OO) |
| Subjective sleep score at one week or later (point) ^[23,31,32,36,37,39,44]^ | Risk of bias ^a^ | Serious ^b^ | No | Serious ^e^ | Strongly suspected ^c^ | 2070 | No difference | Very low quality (⊕OOO) |
| Numeric rating scale of pain at 24 h (point) ^[14,21,22,24,25,28-35,37,38,40-46]^ | Risk of bias ^a^ | Serious ^b^ | No | No | Undetected | 4808 | DEX superior to placebo | Low quality (⊕⊕OO) |
| Morphine equivalent within 7 days (mg) ^[14,15,21,22,24,27,28,33,34,37,43,46]^ | No | Serious ^b^ | No | No | Strongly suspected ^c^ | 1558 | DEX superior to placebo | Low quality (⊕⊕OO) |
| Incidence of delirium after surgery (%) ^[14,15,28,29,32,33,35,38,39,41,43,45,46]^ | Risk of bias ^a^ | No | No | No | Undetected | 3171 | DEX superior to placebo | Moderate quality (⊕⊕⊕O) |
| **Safety outcomes** |  |  |  |  |  |  |  |  |
| Incidence of bradycardia (%) ^[14,15,26,29,30,35,36,38,39,42,43,45]^ | No | No | No | No | Strongly suspected ^c^ | 2818 | DEX inferior to placebo | Moderate quality (⊕⊕⊕O) |
| Incidence of hypotension (%) ^[14,15,26,28-30,35,36,38,39,42,43,45,46]^ | No | No | No | No | Strongly suspected ^c^ | 2992 | DEX inferior to placebo | Moderate quality (⊕⊕⊕O) |

N, number; DEX, dexmedetomidine.

^a^ Many trials were judged to have high or unknow risk of bias. Final decision to rate down quality of evidence by one level for serious limitation.

^b^ I^2^ was above 50% with wide variance of point estimates across studies. Final decision to rate down quality of evidence by one level for moderate or serious inconsistency.

^c^ Final decision to rate down quality of evidence by one level for potential publication bias.

^d^ Five or less trials reported these outcomes. Final decision to rate down quality of evidence by two levels for serious limitation.

^e^ Wide confidence interval with potential clinical impact. Final decision to rate down quality of evidence by one level for serious imprecision.
